# Soma-to-germline miRNA inheritance through yolk promotes stress resilience in progeny

**DOI:** 10.1038/s41594-026-01816-5

**Published:** 2026-05-22

**Authors:** Névé Aupérin, Meetali Singh, Loan Bourdon, Almira Chervova, Julie Ovieve, Pierre-Henri Commere, Hélène Lopez-Maestre, Aikaterini Gkaraveli, Florent Dingli, Damarys Loew, Germano Cecere

**Affiliations:** 1 Mechanisms of Epigenetic Inheritance Unit, Department of Developmental and Stem Cell Biology, Institut Pasteur, CNRS UMR3738, Université Paris Cité, Paris, France; 2 Collège Doctoral, Sorbonne Université, Paris, France; 3 Parasite Epigenetics Lab, Developmental Biology and Genetics, Indian Institute of Science, Bangalore, India; 4 Bioinformatics and Biostatistics Hub, Department of Computational Biology, Institut Pasteur, CNRS USR 3756, Paris, France; 5 Cytometry and Biomarkers UTechS, Institut Pasteur, Paris, France; 6 Centre de Recherche, CurieCoreTech Mass Spectrometry Proteomics, Institut Curie, PSL Research University, Paris, France

**Keywords:** Gene silencing, miRNAs, RNAi, Developmental biology

## Abstract

At the onset of reproduction, oviparous animals synthesize large amounts of yolk in somatic tissues to provide lipids and other nutrients to their progeny. However, whether the yolk transports other molecules, such as RNAs with gene-regulatory functions, remains largely unexplored. Here, we biochemically purified the yolk granules in the nematode *Caenorhabditis* *elegans* and show they contain microRNAs (miRNAs). We provide evidence that the yolk transports miRNAs from the intestine of the mother to the embryos by the lipoprotein yolk receptor RME-2. These yolk-enriched miRNAs inherited by the embryos regulate the transcriptomes of developing larvae. Moreover, environmental stresses and maternal age modulate the transfer of yolk-enriched miRNAs, contributing to stress-resilience benefits to progeny. This discovery establishes an alternative paradigm in intergenerational gene regulation, where the gut–germline axis orchestrates the transmission of environmental cues through yolk-enriched miRNAs. Our work, thus, reveals a mechanism underlying the soma-to-germline transfer of epigenetic information in animals.

## Abstract (web summary)

In this study, the authors use the *Caenorhabditis* *elegans* model organism to show that maternal microRNAs are delivered from the intestine to embryos by the yolk, where they regulate gene expression and promote stress resilience in offspring.

## Main

Inheritance across generations is traditionally attributed to genetic mechanisms but there is growing evidence that epigenetic processes, such as those involving heritable small RNAs, can transmit environmentally induced traits through the germline. Environmental stimuli, including diet and stress, can reshape the small RNA content of gametes, potentially altering gene expression programs in progeny^1,2^. In *Caenorhabditis* *elegans*, both somatic and germline small RNA pathways have been implicated in the inheritance of acquired traits^3–5^. A central question emerging from these findings is whether gametes can acquire epigenetic molecules, such as small RNAs, from somatic tissues^6^.

In oviparous animals like *C*. *elegans*, the yolk is synthesized in somatic tissues, specifically the intestine, starting at the L4 larval stage^7^ and is rapidly transported to developing oocytes to supply essential lipids and nutrients for embryogenesis^8^. Yolk proteins, primarily vitellogenins, are synthesized in the endoplasmic reticulum, trafficked by the coat complex II transport vesicle to the Golgi apparatus and then packaged into exocytic yolk granules that fuse with the intestinal plasma membrane for secretion^9^. These granules are released into the pseudocoelom and then internalized in oocytes by endocytosis through the lipoprotein receptor RME-2 (ref. ^10^). The internalized yolk granules are stable throughout embryogenesis, providing nutritional support throughout embryogenesis and early larval development^11^.

During mid-embryogenesis, the yolk is reallocated from nonintestinal to intestinal embryonic cells, where it is stored in new vesicles for subsequent use^12,13^. While the yolk is not strictly essential for embryonic viability in *C*. *elegans*^14–16^, it enhances progeny development under stressful conditions such as starvation^17–20^ and oxidative stress^21^. In other taxa, the yolk supports immune priming in offspring^22,23^ and influences social behavior^24^. However, the molecular cargo responsible for these effects and the mechanisms of its inheritance remain poorly understood.

The possibility that the yolk transports RNA has long been speculated, with early evidence from amphibians suggesting the presence of RNA in yolk granules^25^. In *C*. *elegans*, however, yolk granules have not been biochemically purified and most insights into yolk biology come from imaging studies. Notably, recent work showed that exogenous double-stranded RNAs injected into the pseudocoelom can be cotransported into oocytes by the yolk^26,27^, raising the question of whether endogenous small RNAs might follow the same route.

Motivated by these observations, we developed a biochemical and genetic strategy to test whether the yolk can serve as a physiological conduit for transferring somatic small RNAs to the germline. By purifying the yolk secreted from intestinal cells before oocyte uptake, we identified a specific enrichment of microRNAs (miRNAs). We demonstrate that intestinal miRNAs are transported by yolk granules to oocytes and contribute to the pool of maternally inherited miRNAs in embryos. These yolk-inherited miRNAs promote larval development, particularly under stress, and are modulated by maternal age and environment. Our findings redefine the role of yolk as not merely a nutrient source but also a vehicle for endogenous RNA transport, enabling soma-to-germline communication with gene-regulatory and epigenetic consequences in the next generation.

## Results

### miRNAs are transported by yolk through the lipoprotein receptor into *C*. *elegans* embryos

**Fig. 1:**
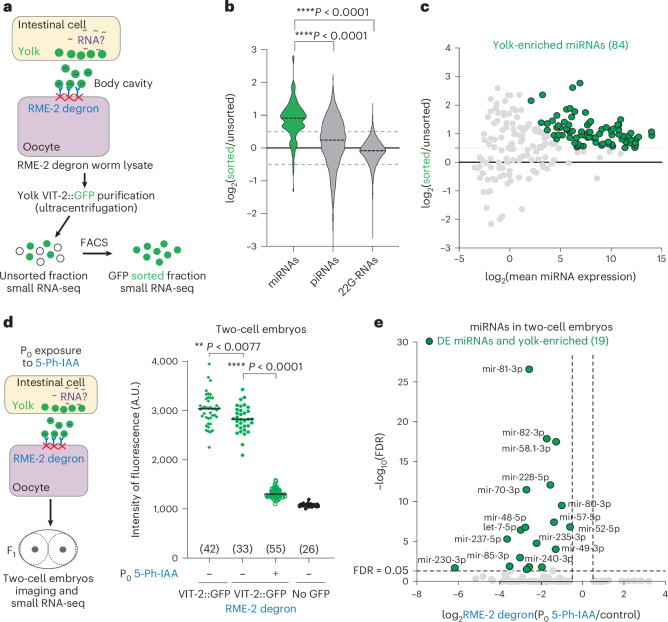
Yolk contributes to the maternal pool of miRNAs in embryos. **a**, Schematic of the VIT-2::GFP yolk purification workflow and small RNA-seq. Genotype of the degron strain used to deplete RME-2 receptor in the germline: gcp88[mex-5p::TIR-1(F79G)::F2A::mTagBFP2::AID*::NLS::tbb-2 3′UTR] (II:0.77); *rme-2*(gcp080[rme-2::AID::2×HA]) IV; vit-2(crg9070[vit-2::gfp]) X. **b**, Violin plot showing the log_2_ fold change of miRNAs, piRNAs and 22G-RNAs between VIT-2::GFP sorted and unsorted small RNA fractions. The dashed line indicates the median. Statistical analysis was performed using two-tailed Mann–Whitney–Wilcoxon tests; ****P < 0.0001 (*n* = 3 biological replicates). **c**, Bland–Altman plot showing log_2_ fold change and mean expression of miRNAs between sorted and unsorted fractions. Green dots indicate yolk-enriched miRNAs (log_2_ fold change ≥ 0.5 and FDR ≤ 0.05; *n* = 3 biological replicates). **d**, Left, schematic of the AID-2 degron system used to deplete the RME-2 yolk receptor with 5-Ph-IAA. Right, VIT-2::GFP fluorescence intensity in two-cell embryos from control (green dots) and wild-type non-GFP (black dots) animals. Statistical analysis was performed using a two-tailed Mann–Whitney–Wilcoxon test; **P = 0.0077 and ****P < 0.0001. Black lines indicate the mean. The sample size (*n*, worms) is indicated in parentheses. **e**, Volcano plot showing log_2_ fold change and significance of miRNA levels in two-cell embryos from RME-2-depleted versus control mothers. Black circles indicate miRNAs with log_2_ fold change ≤ −0.5 or log_2_ fold change ≥ 0.5 and FDR ≤ 0.05; green dots mark yolk-enriched miRNAs from **c** (*n* = 3 biological replicates) (Extended Data Figs. 1 and 2 and Supplementary Tables 2 and 3). Source data

**Extended Data Fig. 1:**
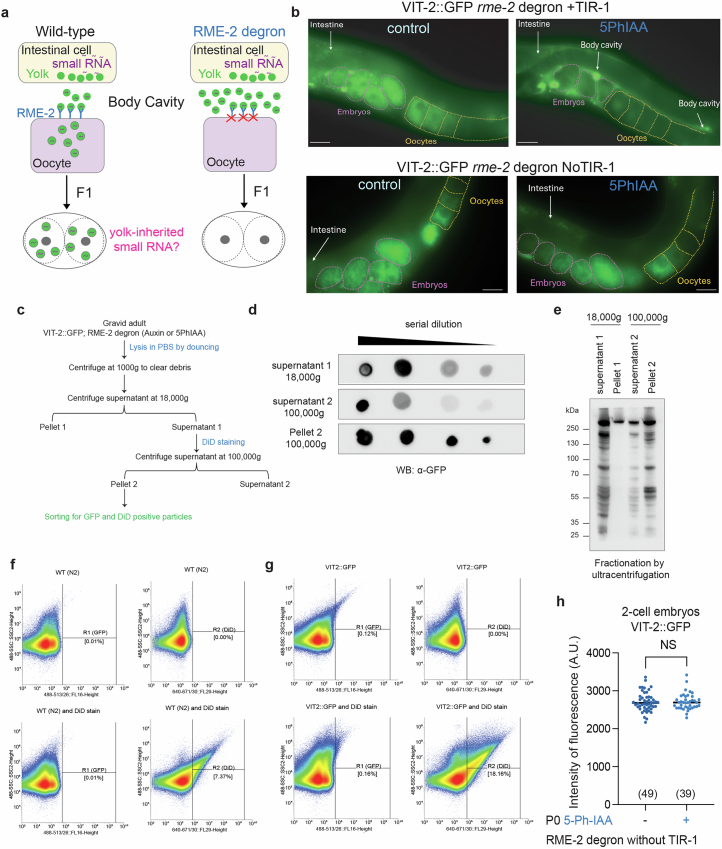
Purification of VIT-2::GFP granules secreted into the body cavity and analysis of associated small RNAs. **a**, Schematic of yolk production and uptake in wild-type and RME-2 degron strains. In wild-type animals, yolk is synthesized in the intestine, secreted into the body cavity, and internalized by oocytes via the RME-2 receptor. In RME-2-depleted animals, yolk granules accumulate in the body cavity due to impaired uptake. **b**, Representative fluorescence images of VIT-2::GFP; RME-2 degron strains treated with or without 5-Ph-IAA in the presence (top) or absence (bottom) of TIR-1. At least 20 worms were analyzed for each condition and showing similar results. Scale bar is 25 μm. Fluorescence microscopy does not resolve the ultrastructural granularity of yolk particles; discrete yolk granules have been resolved previously by immune-electron microscopy^9^. **c**, Schematic of the yolk granule purification protocol: body cavity extracts are subjected to ultracentrifugation and fluorescent particle sorting. **d**, Dot blot analysis showing enrichment of VIT-2::GFP in purification fractions using anti-GFP antibody, corresponding to the scheme in panel **c**. **e**, Representative western blot of VIT-2::GFP using anti-GFP antibody in purification fractions, confirming enrichment as shown in panel **c**. Similar results were obtained in three different experiments. **f**, Sorting profiles of control N2 extracts (top: unstained, bottom: DiD-stained) showing GFP (R1) and DiD (R2) gating. **g**, Sorting profiles of VIT-2::GFP; RME-2 degron extracts (top: unstained, bottom: DiD-stained) showing gating strategy for GFP+ and DiD+ particles. Double-positive particles were sorted. **h**, Quantification of VIT-2::GFP fluorescence intensity in 2-cell embryos from RME-2 degron (no TIR-1) mothers treated with or without 5-Ph-IAA. Statistical analysis was performed using two-tailed Mann–Whitney–Wilcoxon tests. NS, not significant. The number of worms (n) is indicated in parentheses. Source data

To explore whether endogenous small RNAs are transported with yolk into the germline, we first developed a strategy to purify yolk granules before their internalization into oocytes (Fig. 1a). We generated a *C*. *elegans* strain that expresses the yolk protein vitellogenin VIT-2 tagged with GFP^17^ and enables conditional depletion of the RME-2 receptor through auxin-inducible degradation (AID)^28^ in oocytes by expressing TIR-1 under the control of the germline-specific *mex-5* promoter. In the presence of auxin, this RME-2 degron strain accumulated VIT-2::GFP in the body cavity as the yolk cannot be internalized in oocytes and embryos (Extended Data Fig. 1a,b). The RME-2 degron strain allowed us to overcome the fertility and developmental defects observed in the *rme-2* mutant^10,16^.

Using this RME-2 degron strain, we enriched yolk granules by biochemical purification^29^ and purified GFP-positive yolk by fluorescence-activated cell sorting (FACS) (Extended Data Fig. 1c–g). It is important to note that this approach provides a qualitative enrichment rather than a quantitative measurement of yolk content. Consistent with the known physiology of yolk trafficking in *C*. *elegans*, yolk granules are secreted from the intestine in the pseudocoelomic body cavity and are subsequently internalized into developing oocytes and embryos by the yolk receptor RME-2 (ref. ^9^).

To confirm the purity of our yolk granule preparations and exclude contamination from autofluorescent gut granules, we optimized our FACS strategy to exclude particles from wild-type N2 animals lacking GFP::VIT-2 (Extended Data Fig. 1f,g). We also stained with the lipophilic dye DiD to coselect lipid-rich particles positive for both GFP and lipids (Extended Data Fig. 1f,g). Mass spectrometry (MS)-based proteomic analysis of sorted yolk granules identified the six vitellogenin proteins (VIT-1 to VIT-6) and showed the highest peptide counts corresponding to them (Supplementary Table 1). In addition, we identified several RNA-binding proteins previously implicated in miRNA function and stabilization—including VGLN-1, TSN-1, CAR-1, CGH-1, VIG-1, PAB-1 and the chaperones HSP90 and HSP70 (refs. ^30–35^)—suggesting that yolk granules may carry components of RNA–protein complexes relevant to miRNA transport.

**Extended Data Fig. 2:**
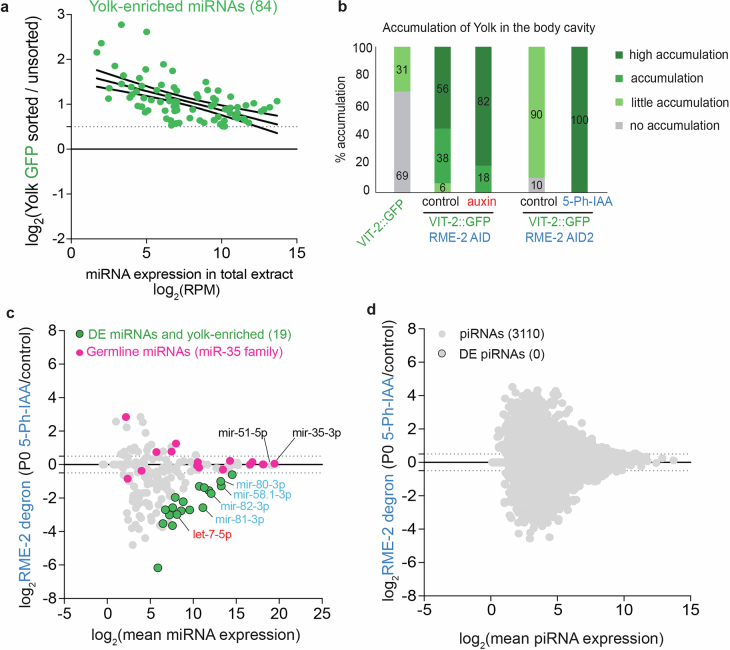
Characterization of RME-2 degron strain and depletion of miRNAs in 2-cell embryos. **a**, MA-plot showing the log2 fold change of yolk-enriched miRNAs and mean expression levels of miRNAs from total worm lysate before yolk purification. *n* = 3 biological replicates. **b**, Bar plot showing VIT-2::GFP accumulation in the body cavity of animals expressing two RME-2 degron versions (AID and AID2). The AID version shows high VIT-2::GFP accumulation even under control conditions, indicating leaky degradation. **c**, MA-plot showing log_2_ fold change and mean expression of miRNAs in 2-cell embryos from control vs. 5-Ph-IAA–treated RME-2 degron mothers. Black circles: differentially expressed miRNAs (log_2_ fold change ≤ -0.5 or ≥ 0.5, FDR ≤ 0.05). Green: yolk-enriched miRNAs; Pink: germline-expressed *miR-35* family; Other colored circles indicate *miR-51* (black), *miR-58* family (blue), and *let-7* family (red). *n* = 3 biological replicates. **d**, MA-plot showing piRNA expression in 2-cell embryos from control vs. 5-Ph-IAA–treated RME-2 degron mothers. Differentially expressed piRNAs (log_2_ fold change ≤ -0.5 or ≥ 0.5, FDR ≤ 0.05) are shown in black circles. *n* = 3 biological replicates. See also Supplementary Table 3. Source data

The sorted GFP fractions and unsorted fractions were subjected to small RNA-seq and abundance ratios were calculated for the three most prominent *C*. *elegans* small RNA families: miRNAs, PIWI-interacting RNAs (piRNAs) and 22G-RNAs antisense to protein-coding genes produced by RNA-dependent RNA polymerases (RdRPs)^36^. We did not observe global enrichment of piRNAs or 22G-RNAs in the sorted GFP-positive yolk fraction (Fig. 1b), despite their high abundance in the germline, suggesting that these small RNAs are not selectively packaged into yolk. In contrast, many miRNAs were enriched in the sorted GFP-positive yolk fraction compared to the unsorted fraction (Fig. 1b,c) and we identified 84 of 452 miRNAs that were enriched in the body cavity with yolk upon loss of RME-2 (log_2_ fold change ≥ 0.5 and *P* ≤ 0.05; Supplementary Table 2). We also compared the level of enrichment of these 84 miRNA with their expression levels in the whole-worm lysates and observed that these enriched miRNAs vary in abundance and are not only the most highly expressed ones (Extended Data Fig. 2a). Therefore, we infer that many miRNAs are selectively enriched with yolk in the body cavity and refer to them as yolk-enriched miRNAs.

To verify that yolk-enriched miRNAs, like yolk, undergo RME-2-mediated endocytosis into oocytes and embryos, we induced the degradation of the receptor by using an improved version of the AID system that implements the auxin derivative 5-phenyl-indole-3-acetic acid (5-Ph-IAA)^37^, which reduces the leaky degradation of the target protein in the absence of auxin. The AID-2 system improved the degradation of RME-2 and avoided the leaky degradation of RME-2 observed in the RME-2 AID strain grown on control plates (Fig. 1d and Extended Data Figs. 1b and 2b). To rule out potential off-target effects of 5-Ph-IAA treatment in our degron experiments, we used RME-2 degron strains that do not express the *TIR1* transgene. In these strains, auxin treatment did not induce VIT-2::GFP accumulation in the pseudocoelom (Extended Data Fig. 1b) or impair yolk inheritance in embryos (Extended Data Fig. 1h), confirming the specificity of our degron-mediated RME-2 depletion.

We depleted the RME-2 receptor starting from the L3 larval stage until gravid adult and collected two-cell embryos before zygotic genome activation and sequenced the maternally inherited miRNAs (Fig. 1d and Methods). Notably, the depletion of RME-2 decreased the levels of 19 miRNAs in two-cell embryos, including many abundant miRNAs, such as the *miR-58* and *miR-51* families (Fig. 1e, Extended Data Fig. 2c and Supplementary Table 3). Moreover, all of these differentially inherited miRNAs were yolk-enriched miRNAs. Additionally, we confirmed that depletion of RME-2 selectively reduced the abundance of yolk-enriched miRNAs in embryos but not inherited germline-expressed miR-35 family or piRNAs, suggesting that yolk depletion does not broadly affect germline function or RNA biogenesis (Extended Data Fig. 2c,d).

Together, these data indicate that miRNAs travel with yolk into oocytes and are inherited in two-cell embryos.

### Yolk-enriched miRNAs originate from the mother’s intestine

**Fig. 2:**
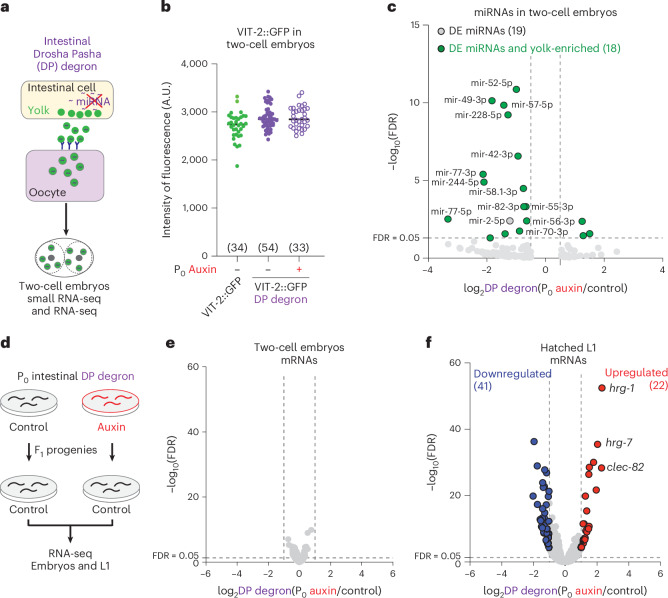
The intestine contributes to yolk-inherited miRNAs and regulates gene expression in larvae. **a**, Schematic of tissue-specific depletion of DP in the intestine using the AID system. Genotype of the degron strain used to deplete Drosha and Pasha specifically in the worms’ intestine: *drsh-1*(luc82[myc::AID::3×FLAG::4×GGSG::drsh-1::4×GGSG::3×FLAG::AID::myc]) *pash-1*(luc71[pash-1::2×GGSG::3×FLAG::AID::myc]) I; reSi12 [ges-1p::TIR-1::F2A::mTagBFP2::AID*::NLS::tbb-2 3′UTR] (II:0.77). **b**, Fluorescence intensity of VIT-2::GFP in two-cell embryos from wild-type (green), DP degron control (filled purple) and DP degron auxin-treated (empty purple) animals. Black lines indicate the mean. The sample size (*n*, worms) is indicated in parentheses. **c**, Volcano plot showing the log_2_ fold change (≤−0.5 or ≥0.5) and significance of miRNA levels in two-cell embryos from DP degron mothers with or without auxin treatment. Black circles indicate miRNAs with log_2_ fold change ≤ −0.5 or log_2_ fold change ≥ 0.5 and FDR ≤ 0.05. Green dots highlight yolk-enriched miRNAs defined in Fig. 1c (*n* = 3 biological replicates). **d**, Schematic of the experimental design used to assess gene expression changes in two-cell embryos and L1 larvae derived from intestinal DP-depleted or control mothers. **e**,**f**, Volcano plots showing log_2_ fold change and significance of mRNA levels in two-cell embryos (**e**) and L1 larvae (**f**) from DP-depleted versus control mothers. Red and blue dots represent significantly upregulated and downregulated genes (log_2_ fold change ≤ −1 or log_2_ fold change ≥ 1, FDR ≤ 0.05; *n* = 2 biological replicates) (Extended Data Figs. 3 and 4 and Supplementary Tables 3 and 4). Source data

**Extended Data Fig. 3:**
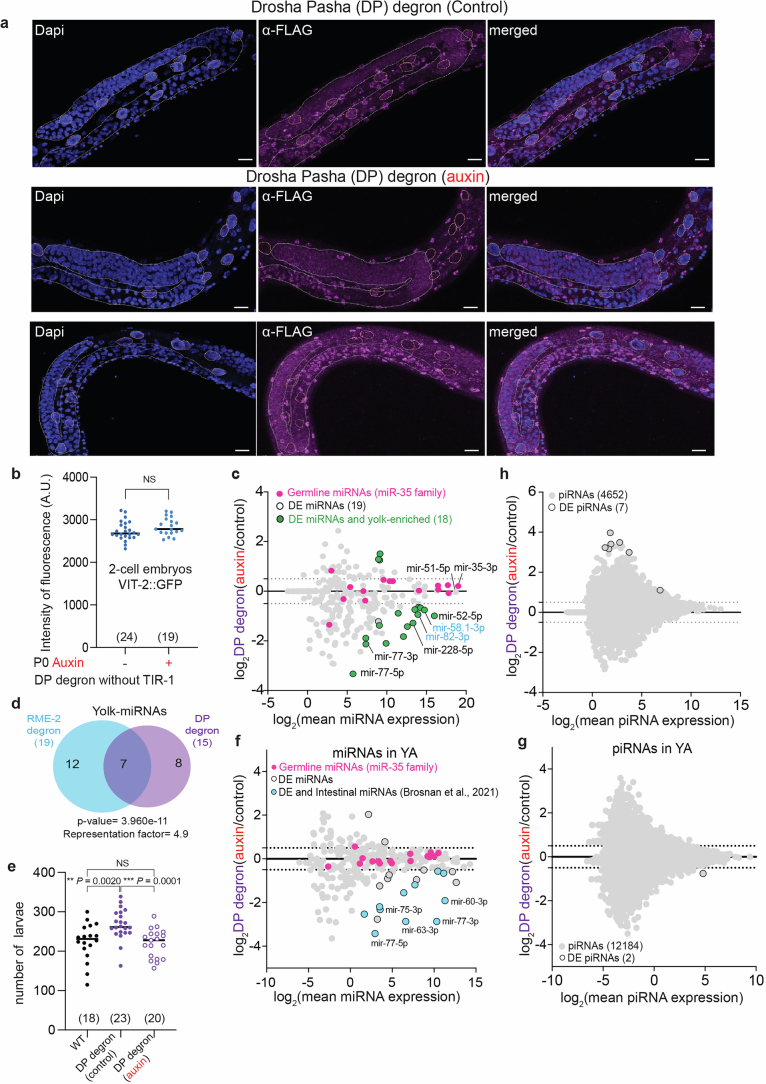
Characterization of 2-cell embryo miRNA pools from Drosha Pasha (DP) degron strain. **a**, α-FLAG immunostaining showing DP localization in the intestine and germline of animals grown on control plates. Auxin treatment induces intestine-specific DP depletion, with germline expression unaffected. Nuclei are counterstained with DAPI (blue). Scale bar, 15 μm. **b**, VIT-2::GFP fluorescence in 2-cell embryos from DP degron animals lacking TIR1 grown with or without auxin. Black lines indicate means. Two-tailed Mann–Whitney–Wilcoxon test; NS, not significant. Sample sizes (*n*) are indicated in parentheses. **c**, MA plot of miRNA expression in 2-cell embryos from control versus auxin-treated DP degron mothers. Black circles indicate differentially expressed miRNAs (log_2_ fold change ≤ −0.5 or ≥ 0.5, FDR ≤ 0.05). Green, yolk-enriched miRNAs; pink, germline-expressed miR-35 family. *n* = 3 biological replicates. **d**, Venn diagram showing overlap of yolk-enriched miRNAs depleted in RME-2 (blue) and DP degron (purple) embryos. P values were calculated using one-sided Fisher’s exact test. **e**, Brood size of WT (N2), DP degron control, and auxin-treated mothers. Black lines indicate means. Two-tailed Mann–Whitney–Wilcoxon test. **P = 0.0020, ***P = 0.0001; NS, not significant. **f**, MA plot of miRNA expression in whole DP degron young adults with or without auxin. Black circles indicate differentially expressed miRNAs (log_2_ fold change ≤ −0.5 or ≥ 0.5, FDR ≤ 0.05). Blue, intestinal miRNAs; pink, miR-35 family. *n* = 3 biological replicates. **g**, MA plot of piRNA expression in DP degron young adults ± auxin. Differentially expressed piRNAs are shown in black (log_2_ fold change ≤ −0.5 or ≥ 0.5, FDR ≤ 0.05). *n* = 3 biological replicates. **h**, MA plot of piRNA expression in 2-cell embryos from DP degron mothers ± auxin. Differentially expressed piRNAs are shown in black. *n* = 3 biological replicates. See also Supplementary Table 3. Source data

To determine whether the maternally inherited miRNAs, like maternally inherited yolk proteins, originate from the mother’s intestine, we conditionally inactivated the crucial miRNA biogenesis enzymes Drosha and Pasha^38^ in intestinal cells. Briefly, we generated an intestine-specific AID strain in which Drosha and Pasha are depleted by expressing TIR-1 under the control of the intestinal-specific *ges-1* promoter. Mothers were exposed to auxin starting from the L3 larval stage (Extended Data Figs. 3a) and two-cell embryos were collected for miRNA-seq (Fig. 2a). We refer to this intestinal Drosha/Pasha depletion strain as the DP degron throughout the paper. VIT-2::GFP levels were similar in two-cell embryos derived from DP degron mothers treated with auxin and from control mothers (Fig. 2b), as well as DP degron strains that do not express the *TIR1* transgene (Extended Data Fig. 3b), suggesting that yolk synthesis and transmission were not compromised by intestine-specific disruption of miRNA biogenesis. Importantly, DP degron mothers lacking intestinal miRNA biogenesis gave rise to two-cell embryos with reduced levels of 16 miRNAs (Fig. 2c, Extended Data Fig. 3c and Supplementary Table 3). Moreover, 15 of these depleted miRNAs were yolk-enriched miRNAs and seven of these 15 were also depleted in embryos upon loss of RME-2 (Extended Data Fig. 3d). We also checked that the tissue-specific degradation of Drosha/Pasha in the mothers’ intestine did not result in severe adverse effects on fertility (Extended Data Fig. 3e) and specifically depleted known intestinal-enriched miRNAs and not piRNAs or germline miRNAs (Extended Data Fig. 3f,g). Moreover, no significant depletion of maternally inherited piRNAs or germline-expressed and inherited miR-35 family was observed in the two-cell embryos (Extended Data Fig. 3c,h).

Overall, these results suggest that miRNAs synthesized in the mother’s intestine are imported into the embryo with the yolk.

### Gene expression changes in embryos derived from mothers depleted of intestinal miRNAs

As post-transcriptional regulators, miRNAs bind to the 3′ untranslated region (3′UTR) of specific mRNAs and trigger their degradation or inhibit translation. We hypothesized that yolk-enriched miRNAs inherited from the intestine regulate mRNA levels in the receiving progeny. To test this, we isolated progeny from DP degron mothers grown on control or auxin plates and performed RNA-seq (Fig. 2d). We isolated progeny at two developmental stages: two-cell embryos and L1 larvae. The two-cell embryos from control-treated and auxin-treated DP degron mothers did not differ significantly (Fig. 2e), suggesting that yolk-enriched miRNAs do not affect the initial maternal mRNA contribution.

**Extended Data Fig. 4:**
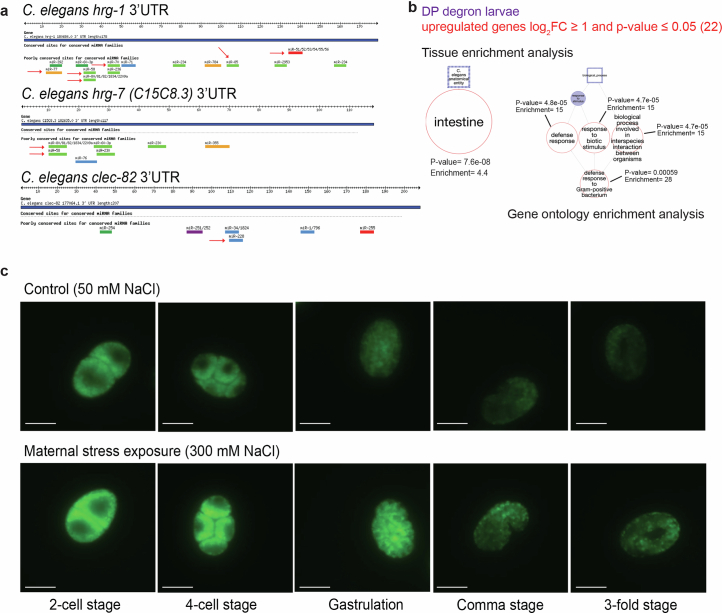
Characterization of inherited miRNA targets in larvae. **a**, Genome browser views showing predicted miRNA binding sites on the 3′UTRs of the three most upregulated genes in DP degron larvae (log_2_ fold change ≥ 2; FDR ≤ 0.05): *hrg-1*, *hrg-7*, and *clec-82*. Target predictions were generated using TargetScanWorm 6.2. Red arrows indicate predicted sites for yolk-enriched miRNAs. **b**, Tissue enrichment and Gene Ontology analyses of upregulated genes in DP degron larvae. P value is calculated using the hypergeometric probability test and corrected using the Benjamini-Hochberg step-up algorithm. **c**, Representative fluorescent images displaying yolk persistence upon development of VIT-2::GFP progeny derived from mothers exposed to osmotic stress (300 mM NaCl, bottom) for 24 h or control (50 mM NaCl, top). Progenies at different embryonic and larval stages are displayed. Scale bar is 25 μm. Similar results were observed in at least three independent experiments. See also Supplementary Tables 3 and 4.

In contrast, L1 larvae from control and auxin-treated DP degron mothers displayed some significant gene expression changes (Fig. 2f and Supplementary Table 4). The most elevated transcripts (log_2_ fold changes > 2), *hrg-1*, *hrg-7* and *clec-82*, are predicted to have miRNA binding sites in their 3′UTR^39^ corresponding to the yolk-enriched *miR-58*, *miR-51* families, *miR-228, miR-85* and *miR-77* (Extended Data Fig. 4a). Intriguingly, some of these miRNAs are yolk-enriched miRNAs that were substantially depleted in embryos lacking yolk or maternal-depleted intestinal miRNAs (Figs. 1e and 2c). These data are consistent with targeted mRNA degradation by yolk-enriched miRNAs in wild-type larvae. Moreover, tissue enrichment analysis^40^ revealed that the elevated mRNAs in L1 larvae correspond to mRNAs that are enriched in the intestine (Extended Data Fig. 4b), where yolk proteins are known to persist in late embryogenesis and freshly hatched larvae^41^ (Extended Data Fig. 4c). Together, these data suggest that yolk-enriched miRNAs might regulate transcript levels in the developing embryos and larvae.

### Maternal age promotes the inheritance of yolk-enriched miRNAs

**Fig. 3:**
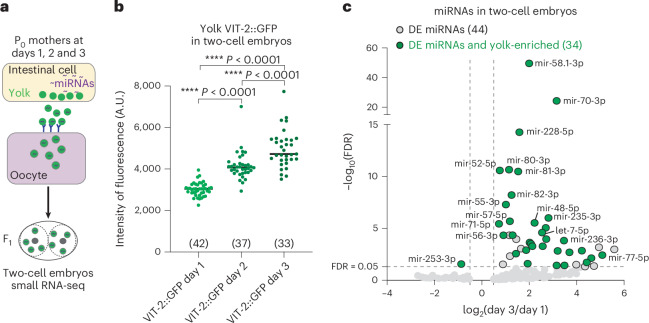
Maternal age promotes the inheritance of yolk-enriched miRNAs. **a**, Schematic of the two-cell embryo collection strategy for small RNA-seq from mothers on day 1, day 2 and day 3. **b**, Fluorescence intensity of VIT-2::GFP in two-cell embryos from mothers of different ages. Statistical analysis was performed using two-tailed Mann–Whitney–Wilcoxon tests; ****P < 0.0001. Black lines represent the mean. The sample size (*n*, worms) is indicated in parentheses. **c**, Volcano plot showing the log_2_ fold change (≤−0.5 or ≥0.5) and corresponding significance of miRNA levels in two-cell embryos from mothers on day 3 versus day 1. Black circles indicate miRNAs with log_2_ fold change ≤ −0.5 or log_2_ fold change ≥ 0.5 and FDR ≤ 0.05. Green dots highlight yolk-enriched miRNAs defined in Fig. 1c (*n* = 3 biological replicates) (Extended Data Fig. 5 and Supplementary Table 3). Source data

**Extended Data Fig. 5:**
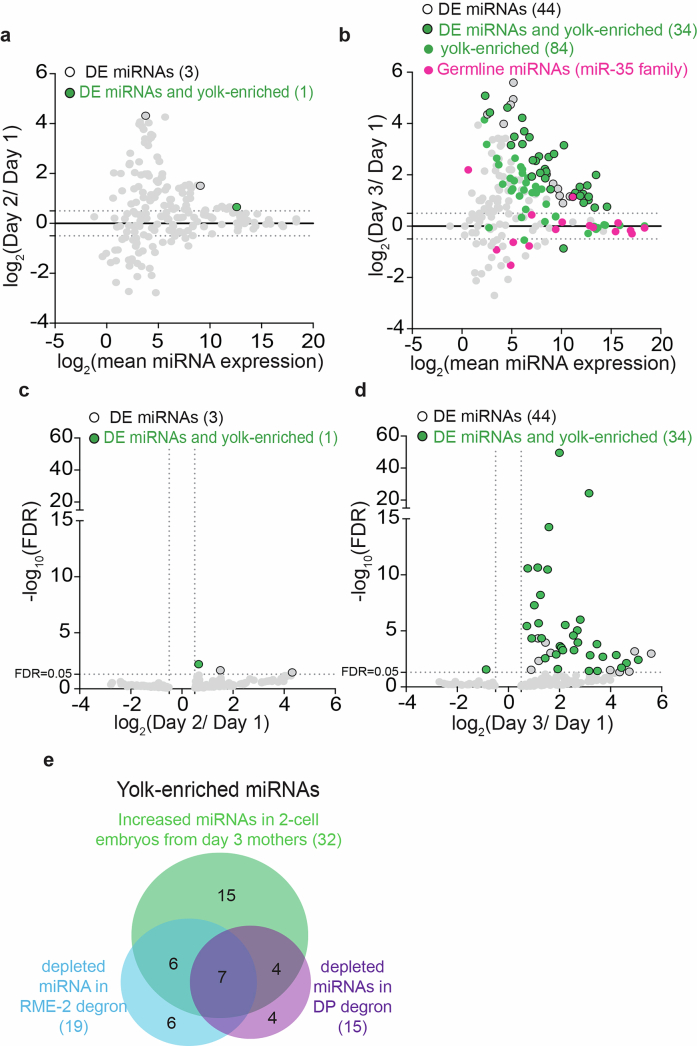
Differential expression of miRNAs in 2-cell embryos derived from Day 1, Day 2, and Day 3 mothers. **a**, **b**, MA plots showing log_2_ fold change versus mean expression of miRNAs in 2-cell embryos collected from Day 2 (**a**) or Day 3 (**b**) mothers compared to Day 1 mothers. Black circles indicate significantly different miRNAs (log_2_ fold change ≤ –0.5 or ≥ 0.5; FDR ≤ 0.05). Green dots highlight yolk-enriched miRNAs; pink dots mark the germline-expressed *miR-35* family. **c**, **d**, Volcano plots showing log_2_ fold change and significance of miRNA expression differences in embryos from Day 2 (**c**) or Day 3 (**d**) mothers versus Day 1. Colour coding is the same as in **a**, **b**. The data in **d** are also shown in Fig. 3c. **e**, Venn diagram showing the overlap between yolk-enriched miRNAs depleted in RME-2 degron (blue) and DP degron (purple) 2-cell embryos and yolk-enriched miRNAs in 2-cell embryos collected from day 3 mother (green). *n* = 3 biological replicates for all panels. See also Supplementary Table 3. Source data

Wild-type worm populations exhibit elevated yolk inheritance with maternal age^17^, which underlies phenotypic differences in genetically identical progeny from young versus old mothers^17,42^. To determine whether maternal age regulates miRNA inheritance in a wild-type population of worms, we collected two-cell embryos from 1-day-old, 2-day-old and 3-day-old mothers. We found that embryos from older mothers displayed elevated VIT-2::GFP levels (Fig. 3a,b), consistent with the increased yolk accumulation in embryos from older mothers^17^. Importantly, the levels of 34 of 84 yolk-enriched miRNAs were significantly elevated in two-cell embryos collected from older versus younger mothers (Fig. 3c, Extended Data Fig. 5a–d and Supplementary Table 3). The miRNAs that increase in embryos from older mothers also largely overlap with the miRNAs that are transmitted from the maternal intestine to the embryo by RME-2 (Extended Data Fig. 5e). We infer that maternal age governs yolk-enriched miRNA inheritance in embryos, impacting the maternal pool of inherited miRNAs. Specifically, embryos from older mothers receive higher levels of miRNAs.

### Yolk-enriched miRNAs confer stress resilience in progeny

Larvae from older mothers inherit more yolk and are more resistant to starvation^17^. We noted that the mRNAs that were elevated in larvae derived from DP degron mothers were enriched for genes involved in biotic and abiotic stresses (Extended Data Fig. 4b). Similarly, some of the yolk-enriched miRNAs that we discovered here are involved in stress responses in *C*. *elegans*^43–48^. These observations suggest that yolk-enriched miRNAs may contribute to the increased stress resilience observed in progeny from older mothers.

**Fig. 4:**
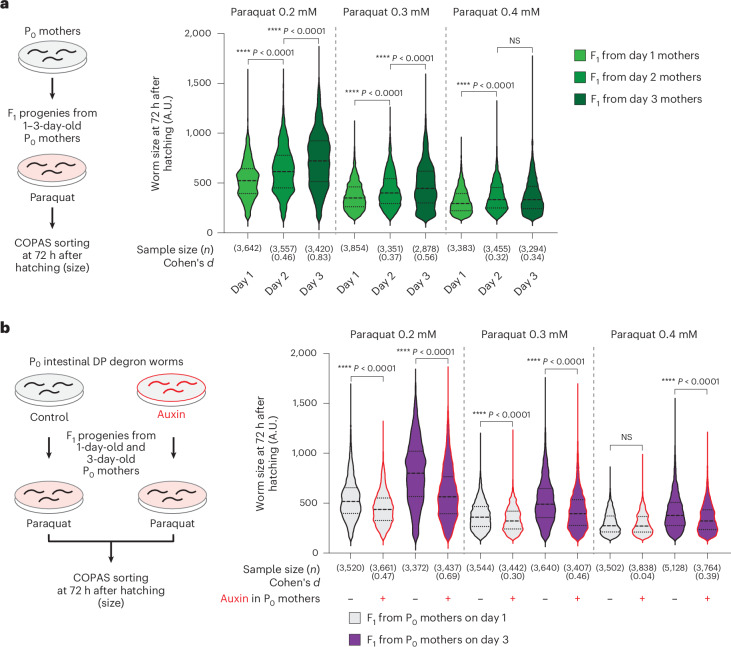
Yolk-enriched miRNAs confer stress resilience in progeny. **a**, Left, schematic of oxidative stress assay in L1 progeny derived from mothers on day 1, day 2, and day 3. Right, violin plots showing the developmental progression of L1 larvae exposed to paraquat (0.2, 0.3 or 0.4 mM) for 72 h after hatching. Development was assessed using COPAS worm sorting. Dashed and dotted lines represent the median and quartiles, respectively. Statistical analysis was performed using a two-tailed Mann–Whitney–Wilcoxon test; ****P < 0.0001 (NS, not significant). The sample size (*n*, worms) is shown in parentheses. **b**, Schematic and results of experiments similar to **a** using the DP degron strain. Mothers were exposed to auxin either from the L3 stage until day 1 or from day 1 to day 3 of egg laying. L1 progeny from auxin-treated mothers displayed reduced growth under oxidative stress compared to controls. Statistical analysis was performed using a two-tailed Mann–Whitney–Wilcoxon test; ****P < 0.0001. Cohen’s *d* effect size was calculated (0.2 = small, 0.5 = medium, 0.8 = large). The sample size (*n*, worms) is shown in parentheses (Extended Data Fig. 6). Source data

**Extended Data Fig. 6:**
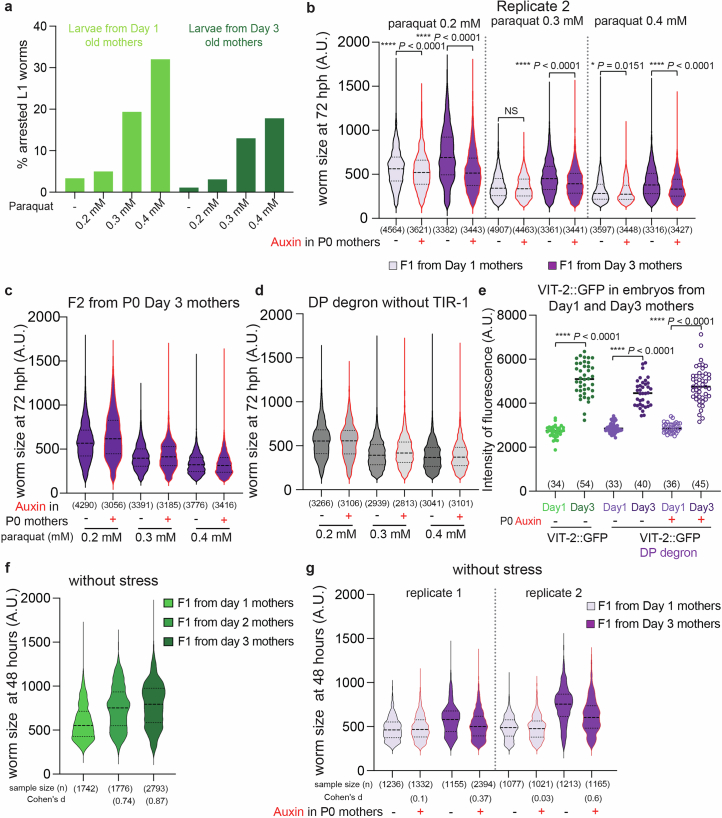
Yolk-enriched miRNAs contribute to oxidative stress resilience and growth advantages associated with maternal age. **a**, Percentage of arrested L1 larvae after 72 h on paraquat (0.2, 0.3, or 0.4 mM). Progeny were derived from wild-type (N2) day 1 or day 3 mothers; larvae remaining at the L1 stage were scored as arrested. **b**, Larval size after 72 h on paraquat for progeny from DP degron mothers collected at day 1 or day 3 of egg laying. Mothers were grown with or without auxin from the L3 stage to induce intestine-specific depletion of miRNA biogenesis. Larval size was measured using a COPAS worm sorter. Statistical analysis was performed using two-tailed Mann–Whitney–Wilcoxon tests. *P = 0.0151; ****P < 0.0001; NS, not significant. **c**, Same assay as in b, using F2 progeny from day 3 DP degron mothers, showing that increased sensitivity to oxidative stress does not persist across generations. **d**, Same assay as in **b**, using progeny from DP degron mothers lacking TIR1, confirming that the effect requires auxin-dependent degron activation. **e**, Quantification of VIT-2::GFP fluorescence in 2-cell embryos from wild-type or DP degron mothers collected at day 1 or day 3 and grown with or without auxin, showing that age-dependent yolk accumulation is preserved upon intestinal DP depletion. Statistical analysis was performed using two-tailed Mann–Whitney–Wilcoxon tests. ****P < 0.0001. Sample sizes are indicated in parentheses. **f**, Violin plots showing larval size after 48 h under standard conditions (no stress) for progeny from day 1–3 wild-type mothers. In the absence of paraquat, development is faster; measurements were therefore performed at 48 h to compare equivalent developmental stages, as animals reach adulthood by 72 h. **g**, Same assay as in f, showing two independent biological replicates for progeny from DP degron mothers grown with or without auxin; effect sizes (Cohen’s *d*) are indicated. For **b–g**, dashed and dotted lines indicate medians and quartiles; sample sizes (worms or embryos) are shown in parentheses. Source data

To investigate this, we examined the relationship between maternal age and oxidative stress. L1 larvae from mothers on day 1, day 2 and day 3 were exposed to increasing concentrations of paraquat for 72 h (Fig. 4a). We measured larval growth quantitatively using the COPAS worm sorter (Methods), which allowed us to measure the length of thousands of larvae. As expected, higher paraquat concentrations impaired development and reduced larval length (Fig. 4a and Extended Data Fig. 6a). Notably, larvae from younger mothers, which inherit lower levels of yolk and yolk-enriched miRNAs, were shorter and more sensitive to oxidative stress than larvae from older mothers (Fig. 4a).

Larvae derived from DP degron mothers with intestine-specific disruption of miRNA biogenesis were significantly more sensitive to paraquat-induced oxidative stress than control larvae and this increased sensitivity was restricted to the immediate progeny (Fig. 4b and Extended Data Fig. 6b–d), consistent with an intergenerational effect of the yolk-associated miRNAs. Importantly, age-dependent yolk accumulation was preserved in DP degron mothers treated with auxin, with embryos from mothers on day 3 inheriting significantly more yolk than those from mothers on day 1 (Extended Data Fig. 6e), indicating that maternal intestinal miRNA depletion does not impair yolk production or age-dependent yolk loading.

To determine whether loss of intestinal miRNAs affects larval growth independently of paraquat exposure, we assessed progeny development under standard laboratory conditions. Because larval development is delayed by paraquat exposure compared to standard laboratory conditions, growth was evaluated at 48 h in the absence of stress and at 72 h under oxidative stress to allow comparison of developmentally matched populations. Under these conditions, larvae derived from young mothers (day 1) developed normally regardless of maternal DP depletion (Extended Data Fig. 6f,g). In contrast, while control larvae derived from older mothers (day 3) displayed accelerated growth relative to progeny from young mothers, this maternal-age-dependent growth advantage was reduced upon intestinal DP depletion (Extended Data Fig. 6f,g).

Together, these results indicate that intestinal miRNAs are not required for baseline larval development but contribute to the enhanced growth and oxidative stress resilience associated with advanced maternal age. Maternal-age-dependent increases in yolk transfer, therefore, support progeny fitness through multiple components, among which yolk-enriched miRNAs have a functional but nonexclusive role.

Because the response to starvation is known to be regulated by maternal age and yolk inheritance^17^, we also examined the potential role of yolk-enriched miRNAs in the resilience to starvation. We starved L1 larvae for 3–14 days and found that L1 larvae from DP degron mothers lacking miRNA biogenesis in the intestine were more sensitive to prolonged starvation than those from control mothers (Supplemental Fig. 1). Overall, these results suggest that the inherited yolk-enriched miRNAs promote progeny development under stressful growth conditions and contribute to the stress resilience of progeny from older mothers.

### Maternal stress influences the inheritance of stress-related yolk-enriched miRNAs

**Fig. 5:**
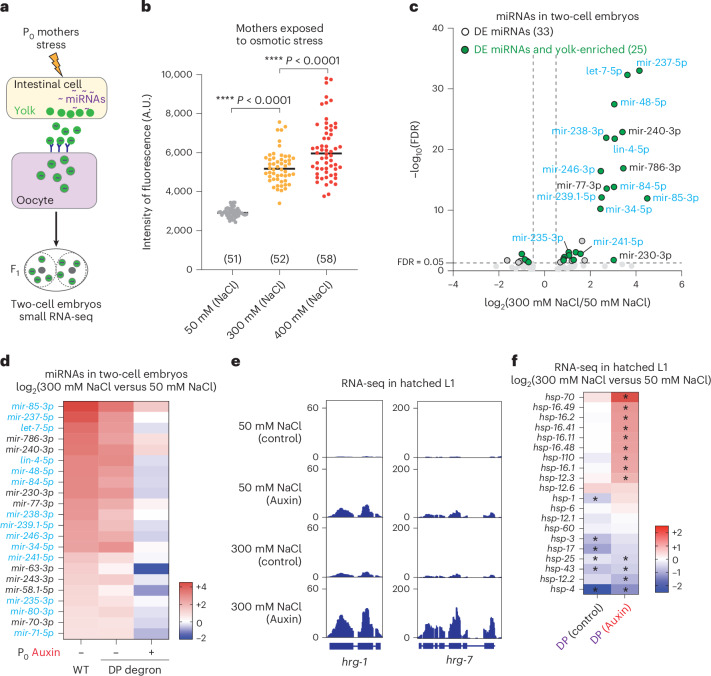
Maternal exposure to stress alters yolk miRNA inheritance and stress adaptation. **a**, Schematic of two-cell embryos collected from mothers exposed to various stress conditions. **b**, Fluorescence intensity of VIT-2::GFP in two-cell embryos derived from mothers exposed to osmotic stress (300 or 400 mM NaCl) for 24 h versus control (50 mM NaCl). Statistical analysis was performed using a two-tailed Mann–Whitney–Wilcoxon test; ****P < 0.0001. Black lines indicate the mean. The sample size (*n*, worms) is shown in parentheses. **c**, Volcano plot showing differential miRNA expression in two-cell embryos from osmotically stressed (300 mM NaCl) versus control (50 mM NaCl) mothers. Black circles indicate miRNAs with log_2_ fold change ≤ −0.5 or log_2_ fold change ≥ 0.5 and FDR ≤ 0.05. Green dots denote yolk-enriched miRNAs (defined in Fig. 1c) and blue-labeled miRNAs were previously implicated in stress responses^45,49–51,53,54^ (*n* = 2 biological replicates). **d**, Heat map showing log_2_ fold changes (300 mM versus 50 mM NaCl) of significantly upregulated miRNAs (log_2_ fold change ≥ 1, FDR ≤ 0.1) in wild-type two-cell embryos compared to DP degron embryos (control or auxin-treated) (*n* = 3 biological replicates). **e**, Genome browser views of *hrg-1* and *hrg-7* mRNAs in hatched L1 larvae from DP degron mothers (control or auxin-treated), exposed to 300 or 50 mM NaCl. The average normalized read counts from three biological replicates are shown. **f**, Heat map showing log_2_ fold changes (300 mM versus 50 mM NaCl) of *hsp* mRNAs in L1 larvae from DP degron mothers (control or auxin-treated; *n* = 3 biological replicates) (Extended Data Fig. 7, Supplementary Table 5 and Supplementary Fig. 1). Source data

**Extended Data Fig. 7:**
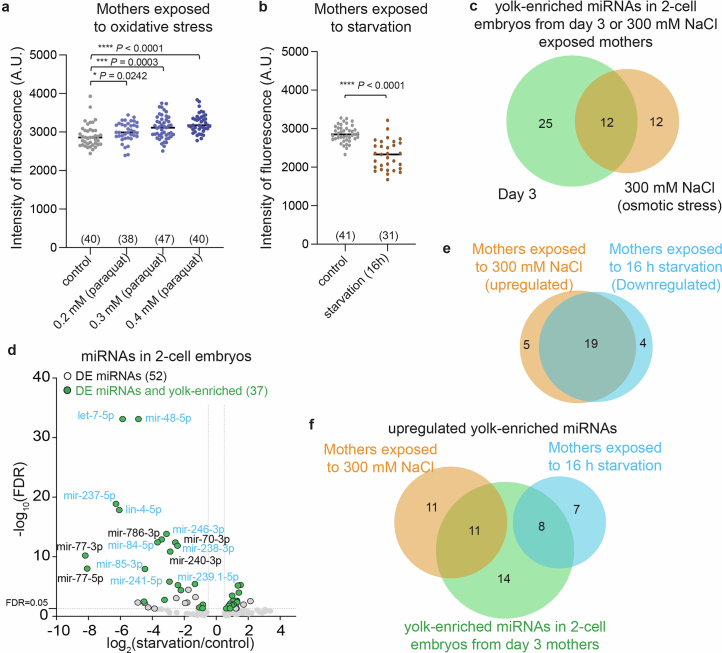
Maternal stress conditions alter yolk content and support miRNA inheritance. **a**, VIT-2::GFP fluorescence intensity in 2-cell embryos from mothers exposed to paraquat-induced oxidative stress (0.2 mM, 0.3 mM, or 0.4 mM) for 24 h compared to controls. Statistical analysis was performed using two-tailed Mann–Whitney–Wilcoxon tests. *P = 0.0242; ***P = 0.0003; ****P < 0.0001. Sample sizes (worms) are shown in parentheses. **b**, VIT-2::GFP fluorescence intensity in 2-cell embryos from mothers starved for 24 h versus controls. Statistical analysis was performed using two-tailed Mann–Whitney–Wilcoxon tests. ****P < 0.0001. Sample sizes (worms) are shown in parentheses. **c**, Venn diagram showing overlap of yolk-enriched miRNAs in 2-cell embryos from day 3 adult mothers and mothers exposed to osmotic stress (300 mM NaCl). **d**, Volcano plot showing differential miRNA expression (black circles, log_2_ fold change ≤ –0.5 or ≥ 0.5; FDR ≤ 0.1) between 2-cell embryos from mothers starved for 16 h versus controls. Green dots indicate yolk-enriched miRNAs identified in Fig. 1c. *n* = 3 biological replicates. **e**, Venn diagram showing overlap of yolk-enriched miRNAs in embryos from mothers exposed to osmotic stress or starvation. **f**, Venn diagram showing overlap of yolk-enriched miRNAs in embryos from day 3 adult mothers and from mothers exposed to osmotic stress or starvation. Source data

To investigate how diverse stresses impact the inheritance of yolk and yolk-enriched miRNAs, we monitored the levels of VIT-2::GFP in two-cell embryos from mothers exposed to osmotic stress, oxidative stress and starvation (Fig. 5a). We observed the largest increase in VIT-2::GFP levels in embryos from mothers exposed to 300 mM NaCl (osmotic stress) compared to 50 mM NaCl (control treatment, regular NGM plates) (Fig. 5b and Extended Data Figs. 4c and 7a). In contrast, two-cell embryos from mothers exposed to 16 h of starvation displayed decreased levels of inherited yolk (Extended Data Fig. 7b).

Next, we sequenced miRNAs in two-cell embryos from mothers exposed to osmotic stress and starvation. Interestingly, embryos from mothers exposed to osmotic stress also displayed elevated levels of many yolk-enriched miRNAs known to have a role in stress responses (Fig. 5c and Supplementary Table 5) and half of them are not enriched in embryos derived from aged mothers, suggesting a specific response to osmotic stress (Extended Data Fig. 7c). The most substantially increased yolk-enriched miRNA in these embryos was *miR-85*, which regulates stress responses by inducing *hsp-70* mRNA degradation upon heat stress^49^. In addition, we found increased levels of the *let-7* miRNA family (*let-7*, *miR-48*, *miR-84* and *miR-241*), which regulate the innate immune response during pathogen-induced stresses^50^; *lin-4* and *miR-235*, which are required during oxidative stress^51,52^; *miR-238*, *miR-239*, *miR-246*, *miR-80* and *miR-71*, which are necessary for the response to heat stress^44,45^; *miR-237*, which is involved in the γ-irradiation-induced stress response^53^; and *miR-34*, which is upregulated during L1 starvation^54^. In contrast, embryos from mothers exposed to starvation inherit decreased levels of many yolk-enriched miRNAs that are increased in embryos from mothers exposed to osmotic stress (Extended Data Fig. 7d,e and Supplementary Table 5). Moreover, the increased inheritance of miRNAs in mothers exposed to stress appeared to be condition specific; starvation, osmotic stress and age each resulted in different subsets of miRNAs being more inherited (Extended Data Fig. 7f), indicating adaptive, nonglobal modulation of yolk miRNA composition.

**Extended Data Fig. 8:**
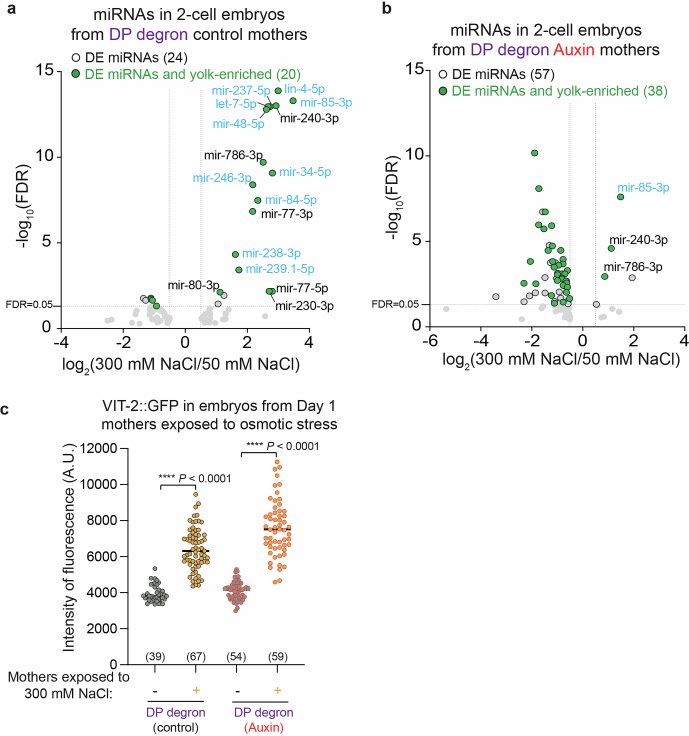
Yolk-enriched miRNA inheritance from the intestine is influenced by osmotic stress. **a**, **b**, Volcano plots showing differential miRNA expression in 2-cell embryos derived from DP degron mothers exposed to osmotic stress (300 mM NaCl) compared to control conditions (50 mM NaCl). Panel **a** shows embryos from DP degron mothers grown without auxin (control), whereas panel **b** shows embryos from DP degron mothers grown with auxin to induce intestine-specific depletion of miRNA biogenesis. Differentially expressed miRNAs (log_2_ fold change ≥ 0.5 or ≤ –0.5; FDR ≤ 0.1) are shown in black; miRNAs identified as yolk-enriched (Fig. 1c) are highlighted in green. *n* = 3 biological replicates. **c**, Quantification of VIT-2::GFP fluorescence intensity in embryos derived from day 1 DP degron mothers exposed to osmotic stress (300 mM NaCl) or control conditions (50 mM NaCl), grown with or without auxin. Statistical analysis was performed using two-tailed Mann–Whitney–Wilcoxon tests. ****P < 0.0001. Sample sizes (embryos) are shown in parentheses. See also Supplementary Table 5. Source data

To verify that these miRNAs are inherited from the intestine, we exposed DP degron mothers to osmotic stress. We observed increased stress-related miRNAs in embryos from control mothers exposed to mild osmotic stress, similar to wild-type mothers (Fig. 5d, Extended Data Fig. 8a and Supplementary Table 5). In contrast, stress-related miRNAs were not highly elevated in embryos derived from auxin-treated DP degron mothers exposed to osmotic stress (Fig. 5d, Extended Data Fig. 8b and Supplementary Table 5), despite inheriting comparable amounts of yolk (Extended Data Fig. 8c). Overall, our data suggest that maternal exposure to osmotic stress or starvation impacts the transmission of yolk and stress-related miRNAs from the intestine to the embryo.

**Extended Data Fig. 9:**
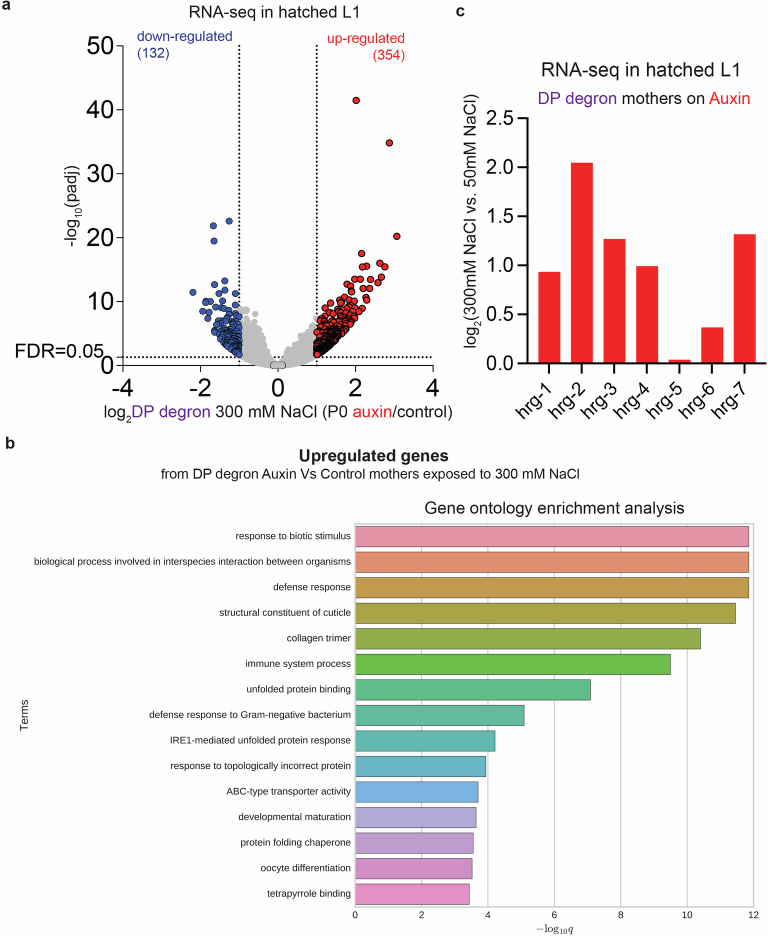
Gene expression changes in hatched L1 larvae derived from osmotically stressed mothers. **a**, Volcano plots showing the log_2_ fold change and corresponding significance levels of the ratio of mRNA levels between DP depleted (auxin) and control L1 larvae from mothers exposed to 300 mM NaCl (osmotic stress). Red and blue dots correspond to differentially expressed genes (considering log_2_ fold change ≤ -1 or ≥ 1 and FDR ≤ 0.05). Differential expression was assessed using DESeq2. *n* = 3 biological replicates. **b**, Tissue enrichment and Gene ontology enrichment analyses of upregulated genes in DP degron larvae derived from mothers exposed to 300 mM NaCl (osmotic stress). **c**, Levels of *hrg* mRNAs in hatched L1 DP degron larvae derived from mothers exposed to 300 mM NaCl (osmotic stress) versus 50 mM NaCl (control). See also Supplementary Table 6. Source data

Next, we tested whether the inheritance of stress-related miRNA from mothers exposed to osmotic stress is required to inhibit stress-related transcripts in developing larvae. We performed RNA-seq on hatched L1 larvae derived from DP degron mothers exposed to osmotic stress or control conditions in the presence or absence of auxin. We found significant gene expression changes in hatched L1 larvae from stressed mothers depleted of stress-related miRNAs (Extended Data Fig. 9a) and the upregulated genes were enriched for genes involved in stress responses and protein folding chaperones (Fig. 5e,f and Extended Data Fig. 9b). Among the upregulated chaperone transcripts, the most upregulated one was *hsp-70* (Fig. 5f), which is directly regulated by *miR-85* (ref. ^49^) and is the highest increased stress-related miRNA in wild-type or control worms (Fig. 5c,d). Interestingly, *hrg* transcripts, which we found among the most upregulated transcripts in larvae depleted of intestinal-inherited miRNAs (Fig. 2f), were further elevated in larvae derived from mothers exposed to osmotic stress (Fig. 5e and Extended Data Fig. 9c).

These results suggest that the inheritance of stress-related miRNAs from mothers exposed to osmotic stress is necessary to repress stress-related transcripts in progeny developed under normal growth conditions after maternal stress.

### Functional role of stress-enriched yolk-inherited *miR-85* in progeny gene regulation

**Extended Data Fig. 10:**
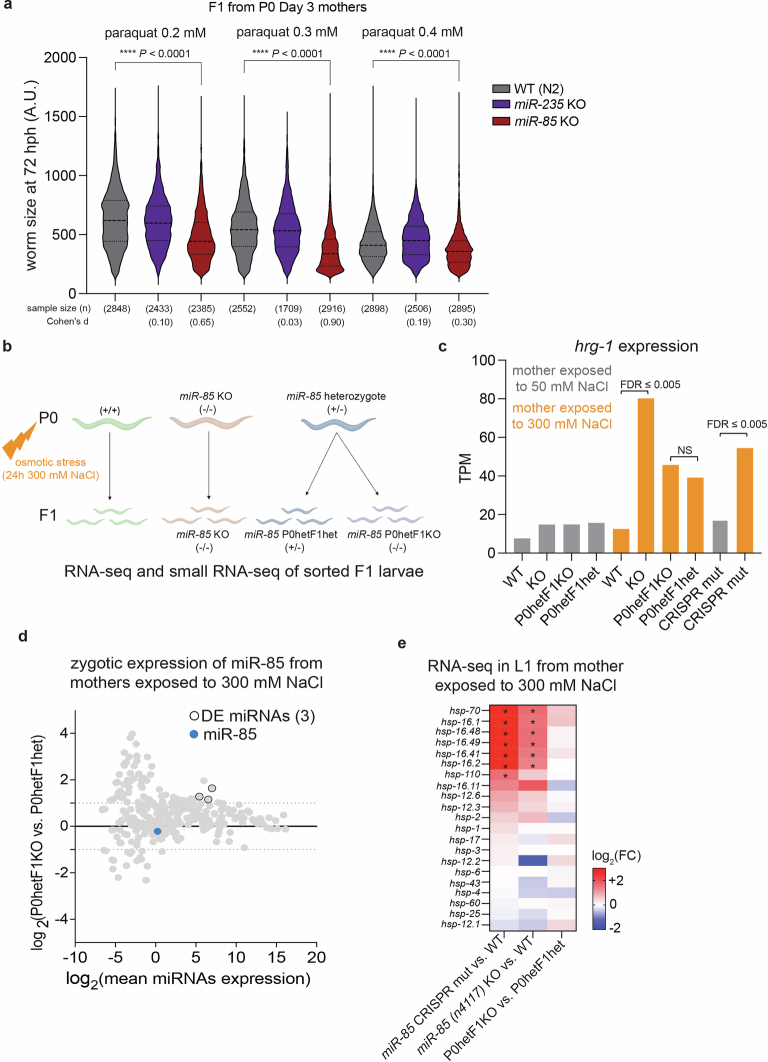
Functional contribution of *miR-85* to progeny stress responses and gene regulation. **a**, Violin plots showing larval size after 72 h on paraquat (0.2, 0.3, or 0.4 mM) for progeny from wild-type (N2), *mir-235* knockout, or *mir-85(n4117)* knockout mothers collected at day 3 of egg laying. Development was quantified by COPAS sorting. Statistical analysis was performed using two-tailed Mann–Whitney–Wilcoxon tests. ****P < 0.0001. Sample sizes (worms) and Cohen’s *d* effect sizes are indicated. **b**, Experimental design for RNA-seq and small RNA-seq analyses. L1 progeny were collected from *mir-85* wild-type, knockout (*n4117*), or heterozygous mothers exposed to control (50 mM NaCl) or osmotic stress (300 mM NaCl). “P0 het F1 KO” denotes homozygous knockout progeny from heterozygous mothers; “P0 het F1 het” denotes heterozygous progeny expressing zygotic *miR-85*. Genotypes were sorted using a fluorescent balancer and COPAS. **c**, Bar plot showing *hrg-1* expression in L1 progeny described in **b**, including wild-type, *mir-85(n4117)* knockout, heterozygous progeny, and an independent CRISPR–Cas9 *mir-85* mutant. Expression is shown relative to the corresponding genetic background and maternal condition, with consistent *hrg-1* upregulation upon loss of *miR-85* across alleles. Differential expression was assessed using DESeq2; FDR ≤ 0.005; NS, not significant. **d**, MA plot of miRNA expression comparing heterozygous and homozygous *mir-85* mutant L1 progeny from heterozygous mothers under osmotic stress. Differentially expressed miRNAs (log_2_ fold change ≥ 1 or ≤ −1; FDR ≤ 0.05) are shown in black; *miR-85* is highlighted in blue. **e**, Heatmap of log_2_ fold changes in selected heat shock protein (*hsp*) mRNAs in hatched L1 larvae from mothers exposed to 300 mM NaCl. Expression changes are shown for the CRISPR–Cas9 *mir-85* mutant, the *mir-85(n4117)* allele, and corresponding controls. Asterisks indicate significance (FDR ≤ 0.05). For **c–e**, RNA-seq analyses were performed using DESeq2; *n* = 3 biological replicates. See also Supplementary Table 6. Source data

To explore whether inherited yolk miRNAs contribute to phenotypic resilience, we examined larval development in progeny derived from mothers lacking specific stress-responsive miRNAs. We focused on *miR-85*, the most increased yolk-inherited miRNA in embryos from osmotically stressed mothers, and on *miR-235*, an miRNA previously implicated in oxidative stress regulation. Larvae lacking *mir-85* exhibited reduced developmental progression under oxidative stress compared to the wild type (Extended Data Fig. 10a). In contrast, *miR-235* mutants showed no developmental defects under same conditions (Extended Data Fig. 10a). These results support a model in which only a subset of stress-induced, yolk-inherited miRNAs, such as *miR-85*, may exert functional heritable effects in progeny, while others may be passively cotransported without measurable impact or to be important for other biological processes not assessed here.

**Fig. 6:**
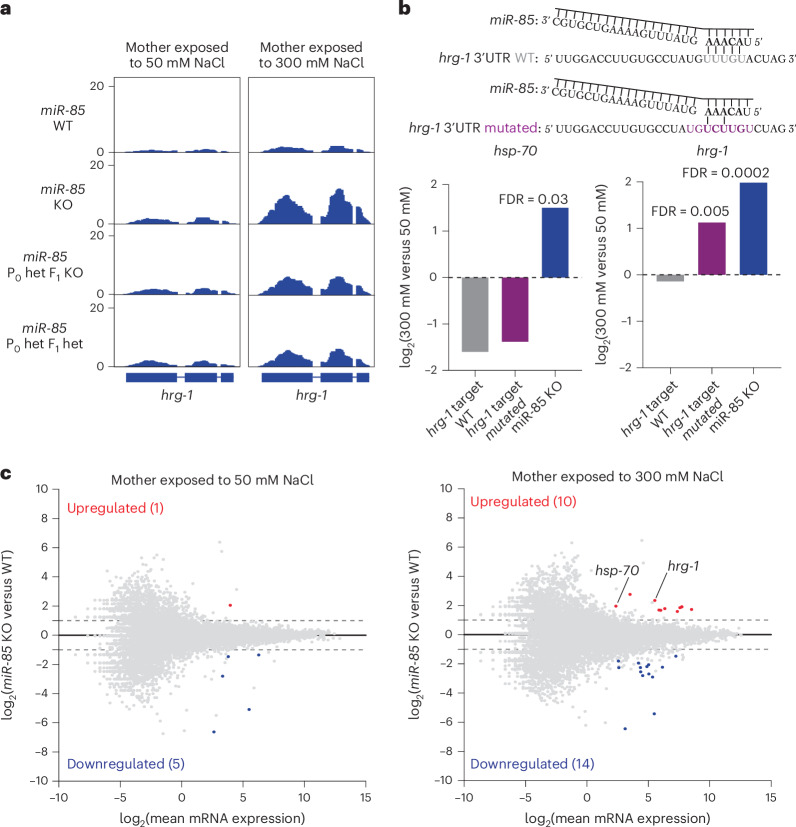
Functional role of yolk-inherited *miR-85* in progeny gene regulation. **a**, Genome browser view of *hrg-1* mRNA in hatched L1 larvae derived from *miR-85* wild-type, KO or heterozygous (P_0_ het) mothers exposed to 300 mM NaCl (osmotic stress) or 50 mM NaCl (control). ‘P_0_ het F_1_ KO’ refers to progeny from heterozygous mothers that are homozygous KO for *miR-85*. ‘P_0_ het F_1_ het’ indicates heterozygous progeny expressing one zygotic copy of *miR-85*. A fluorescence balancer enabled sorting of KO, wild-type and heterozygous mother and progeny using COPAS (Methods and Extended Data Fig. 8b). Normalized read averages from three biological replicates are shown. WT, wild type. **b**, Top, schematic of the predicted interaction between *miR-85* and the wild-type *hrg-1* 3′UTR or the CRISPR–Cas9-engineered seed sequence nucleotide substitution disrupting the *miR-85*-binding site. Bottom, RNA-seq-based quantification of *hrg-1* and *hsp-70* expression in L1 larvae carrying either a wild-type *hrg-1* 3′UTR (target) or the *miR-85*-binding-site mutation (mutant target), derived from mothers exposed to 300 mM NaCl (osmotic stress) or 50 mM NaCl (control). Disruption of the *miR-85*-binding site selectively increased *hrg-1* expression under stress, phenocopying *miR-85* loss, whereas *hsp-70* upregulation was observed only in *miR-85* mutant animals, supporting locus-specific *cis*-regulation at *hrg-1*. Differential expression was assessed using DESeq2 (*n* = 5 biological replicates for the *hrg-1*-binding-site mutant and control, *n* = 2 biological replicates for the *miR-85* KO). FDRs are indicated. **c**, Bland–Altman plots showing gene expression differences between L1 larvae from *miR-85*-KO versus wild-type mothers: left, under control conditions (50 mM NaCl); right, under osmotic stress (300 mM NaCl). Red and blue dots denote significantly upregulated and downregulated genes, respectively (log_2_ fold change ≥ 1, FDR ≤ 0.05; *n* = 2 biological replicates) (Extended Data Figs. 5 and 6 and Supplementary Table 6). Source data

To determine whether *miR-85* regulates gene expression in progeny derived from osmotically stressed mothers, we performed RNA-seq on L1 larvae from *miR-85(n4117)* mutant and wild-type animals (Extended Data Fig. 10b). We first assessed whether *miR-85* affects the expression of *hrg-1*, which contains a predicted *miR-85*-binding site and was among the most upregulated transcripts in the progeny of DP-depleted mothers. RNA-seq analysis revealed that *miR-85* mutant L1 larvae derived from osmotically stressed mothers exhibited significantly elevated levels of *hrg-1* mRNA compared to wild-type progeny (Fig. 6a and Extended Data Fig. 10c). Comparable upregulation was observed using an independently generated CRISPR–Cas9 *miR-85* mutant allele, indicating that *hrg-1* derepression is not allele specific (Extended Data Fig. 10c).

To test whether this repression is mediated through direct interaction of *miR-85* with the *hrg-1* 3′UTR, we generated a CRISPR–Cas9 allele mutating the predicted *miR-85*-binding site (Fig. 6b). Animals harboring this mutation showed significantly increased *hrg-1* expression in L1 larvae derived from osmotically stressed mothers, phenocopying *miR-85* loss at the level of gene expression (Fig. 6b). In contrast, *hsp-70*, which was previously validated as a *miR-85* target in an independent study^49^, was upregulated in *miR-85*-mutant animals but not in the *hrg-1* binding-site mutant, supporting the specificity of the *cis*-regulatory effect at the *hrg-1* locus (Fig. 6b).

To determine whether this regulation was because of maternal or zygotic *miR-85*, we compared *hrg-1* expression in heterozygous and homozygous *miR-85* mutant L1 larvae derived from stressed *miR-85* heterozygous mothers (Extended Data Fig. 10b). We found no significant differences in *hrg-1* expression between these two genotypes (Fig. 6a and Extended Data Fig. 10c) and *miR-85* levels remained low in both, suggesting a lack of zygotic expression at this stage (Extended Data Fig. 10d). Interestingly, we observed a modest increase in *hrg-1* mRNA in heterozygous progeny compared to the wild type, indicating dose sensitivity to inherited *miR-85* (Fig. 6a and Extended Data Fig. 10c).

Together, these results demonstrate that yolk-inherited *miR-85* can directly modulate *hrg-1* expression in progeny in a sequence-specific and inheritance-dependent manner.

Next, we compared the global transcriptional effects of *miR-85* mutation to those observed upon DP depletion. Under control conditions, *miR-85* mutant progeny exhibited only one upregulated gene compared to the wild type (Fig. 6c and Supplementary Table 6) and only ten genes were upregulated under osmotic stress (Fig. 6c), including several *hsp* mRNAs (Extended Data Fig. 10e). RNA-seq analysis further revealed that an independent CRISPR–Cas9 *miR-85* mutant recapitulates the stress-induced *hsp* gene expression signature observed in the original *miR-85(n4117)* allele, supporting a specific role for *miR-85* in regulating a restricted set of stress-responsive targets (Extended Data Fig. 10e). In contrast, hundreds of genes were differentially expressed in the progeny of DP-depleted mothers (Extended Data Fig. 9a), consistent with the broader impact of disrupting yolk-inherited miRNAs. These findings indicate that while the maternal intestine produces multiple yolk-enriched miRNAs with cumulative effects, individual miRNAs such as *miR-85* regulate a narrow and specific set of targets.

### Yolk-enriched miRNA inheritance participates in heritable stress responses

**Fig. 7:**
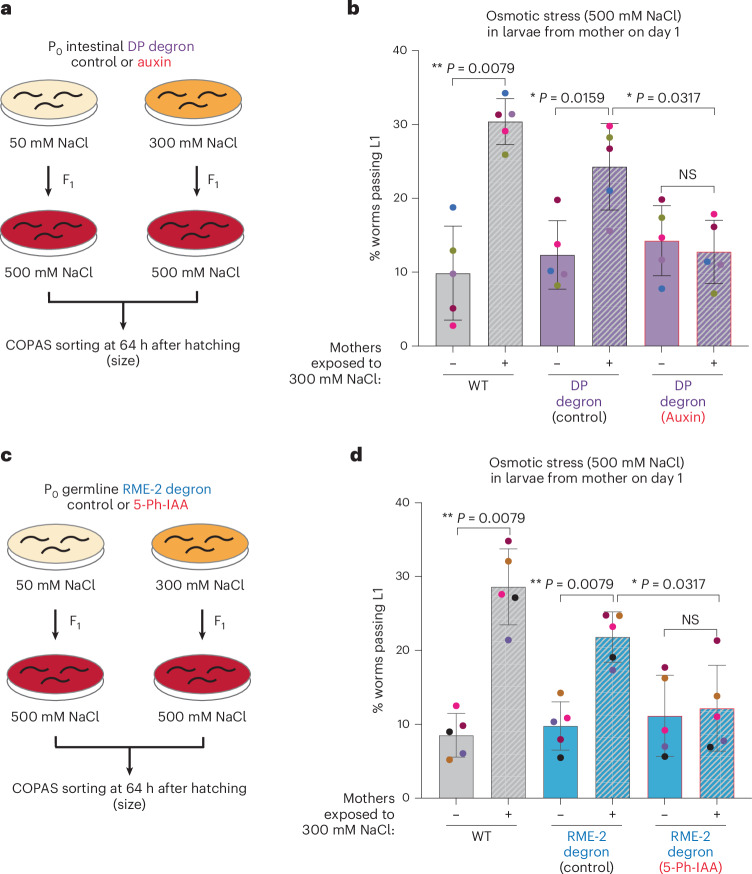
Yolk-enriched miRNA inheritance contributes to heritable stress responses. **a**, Schematic of the assay to assess progeny development under osmotic stress (500 mM NaCl), using the DP degron strain. Mothers were preexposed to mild osmotic stress (300 mM NaCl for 24 h) or control conditions (50 mM NaCl). **b**, Developmental outcomes of progeny from the experiment in **a**, measured at 64 h after hatching using the COPAS worm sorter. Worms passing L1 are those that successfully develop beyond the L1 larval stage despite exposure to 500 mM osmotic stress. The wild-type N2 strain (gray bars) served as a positive control for osmotic stress response^55,56^, while DP degron animals (purple bars) were used to assess yolk miRNA involvement. Data represent the mean ± s.d. Each dot corresponds to an independent biological replicate (*n* = 5), with at least 2,000 worms analyzed per replicate. Statistical analysis was performed using two-tailed Mann–Whitney–Wilcoxon tests; *P = 0.0159–0.0317 and **P = 0.0079. **c**, Schematic of a similar assay using the RME-2 AID-2 degron strain to evaluate the role of yolk uptake in miRNA-mediated inheritance. **d**, Developmental outcomes of progeny from the experiment in **c**, measured at 64 h after hatching using the COPAS worm sorter. The wild-type N2 strain (gray bars) was used as a positive control and RME-2 AID-2 degron animals (blue bars) were tested for yolk uptake dependency. Data represent the mean ± s.d. Each dot corresponds to an independent biological replicate (*n* = 5), with at least 2,000 worms analyzed per replicate. Statistical analysis was performed using two-tailed Mann–Whitney–Wilcoxon tests. Exact *P* values are indicated. Source data

Notably, parental exposure to mild osmotic stress improves the development of progeny exposed to even higher osmotic stress^55–57^. However, yolk inheritance and miRNAs were not implicated as a potential mechanism. To determine whether yolk or yolk-enriched miRNAs function in this heritable adaptation to stress, we used RME-2 degron and DP degron mothers, respectively. The parental degron strains and wild-type controls were treated for 24 h with 50 mM NaCl (control) or 300 mM NaCl (mild osmotic stress) in the presence or absence of auxin (Fig. 7a,c). The larvae were then exposed to 500 mM NaCl (high osmotic stress) for 64 h after hatching and their development was assessed by COPAS sorting (Fig. 7a,c). Larvae from mothers exposed to mild osmotic stress and grown in high-osmotic-stress conditions showed improved escape and progression from L1 larval arrest, as expected (Fig. 7b,d, gray bars). Importantly, this inherited stress resilience was abolished not only by blocking the inheritance of yolk (Fig. 7d) but also by blocking intestinal miRNA biogenesis in the mother (Fig. 7b). Therefore, yolk transmission is necessary but not sufficient for the increased stress resilience in larvae derived from mothers exposed to mild osmotic stress and the inheritance of this stress response requires yolk-enriched miRNAs from the maternal intestine. We also tested the contribution of *miR-85* to osmotic stress resilience. Loss of *miR-85* partially reduced but did not abolish the inherited stress resilience conferred by maternal exposure to osmotic stress, indicating that miR-85 acts together with additional yolk-inherited miRNAs to promote progeny adaptation (Supplementary Fig. 2).

We conclude that miRNAs transmitted from the mother’s intestine through the yolk participate in the heritable adaptation to osmotic stress responses in progeny.

## Discussion

In this study, we demonstrate that miRNAs produced in the maternal intestine are transferred to the germline by yolk granules in *C*. *elegans*. These yolk-enriched miRNAs are internalized into oocytes and embryos through the yolk receptor RME-2, revealing a physiological route for the somatic transmission of small RNAs across generations. We provide compelling evidence that many miRNAs inherited by embryos originate from the mother’s intestine and contribute to gene regulation in early embryos and larvae, thereby extending the functional relevance of yolk beyond its traditional role as a nutrient reservoir.

We further show that maternal age and environmental stress modulate the abundance of yolk-associated miRNAs transmitted to progeny. Older and stressed mothers transmit higher levels of miRNAs, which in turn contribute to larval resilience to oxidative stress and nutrient deprivation. This establishes a direct molecular link between maternal experience and offspring phenotypic plasticity. The selective packaging and delivery of stress-responsive miRNAs by yolk granules represents a previously unrecognized mechanism by which environmental information is conveyed across generations, modulating gene expression and development in the progeny.

Our findings reveal that yolk-mediated delivery of maternal miRNAs critically shapes gene expression programs in the progeny. Notably, members of the *hrg* family, such as *hrg-1*, emerge as key targets whose expression is finely tuned by maternally inherited miRNAs. We demonstrate that *miR-85*, specifically transmitted by the yolk from mothers subjected to osmotic stress, exerts direct post-transcriptional repression on *hrg-1* through a sequence-specific interaction. Mutation of the predicted *miR-85*-binding site within the *hrg-1* 3′UTR abolishes this repression, which occurs independently of zygotic *miR-85* expression, providing clear evidence of sequence-specific regulatory activity of inherited *miR-85*. Given the role of *hrg* genes in modulating intracellular heme homeostasis, their dysregulation may profoundly influence the physiology and developmental trajectories of progeny. Such targeted miRNA delivery may help restrain inappropriate activation of stress-responsive pathways during early development, thereby priming progeny for enhanced resilience to subsequent environmental challenges. Furthermore, tissue-specific enrichment analyses of upregulated mRNAs suggest that these yolk-inherited miRNAs predominantly operate within the larval intestine, aligning with the persistence of yolk aggregates in this tissue during early development^41^ (Extended Data Fig. 4c).

Among the three major small RNA families, only miRNAs were selectively enriched in yolk and transported into embryos, revealing a targeted transport mechanism. One possibility is that piRNAs and 22G-RNAs, while abundant in the germline, are predominantly retained within germline-associated Argonaute complexes and are, therefore, less available for incorporation into yolk particles produced in the maternal intestine, whereas miRNAs produced in somatic tissues may be more readily packaged. Consistent with this idea, biochemical purification of yolk from the body cavity revealed mature, single-stranded miRNAs. Given the lipoprotein-rich nature of yolk, it is plausible that these miRNAs are stabilized through association with lipid particles, reminiscent of extracellular miRNA transport by lipoproteins in mammals^58,59^.

While we did not detect Argonaute proteins in our proteomic analysis of sorted yolk granules, this may reflect technical limitations in sensitivity or the low abundance of purified material. Nonetheless, along with the six vitellogenin proteins, we identified several RNA-binding proteins known to participate in miRNA regulation and silencing^30–35^, including TSN-1, VGLN-1, CAR-1, VIG-1, PAB-1, CEY-2 and the HSP90–HSP70 chaperone complex (Supplementary Table 1). These may help stabilize miRNAs during transport and facilitate their loading into Argonautes in the embryo.

Supporting this model, a previous proteomic analysis of AIN-2 (a GW182 homolog and miRNA effector complex component) identified VIT-6 along with RNA-binding proteins such as TSN-1, CAR-1 and PAB-1 in immunoprecipitates from *C*. *elegans* lysates^30^. Intriguingly, all of these proteins were also detected in our proteomic analysis of purified yolk granules. These overlaps suggest that miRNAs might be transported in a preloading, possibly single-stranded, RNP-bound state and that yolk-associated components of the miRNA silencing complex could facilitate their eventual incorporation into functional silencing complexes in the embryo.

Upon endocytic uptake of yolk granules into late-stage oocytes, miRNAs are likely released into the cytosol during endosome maturation, where pH-dependent membrane remodeling facilitates the escape of RNA cargo. Single-stranded small RNAs can be efficiently loaded into Argonaute proteins and mediate target repression^60^. Additionally, miRNA silencing complexes, including Argonautes, are enriched in late endosomes and multivesicular bodies^61,62^, with miRNA loading shown to be pH dependent during endosomal maturation^63^.

Recent studies have also shown that Argonaute proteins are generally absent from exosomes^64^, suggesting that miRNAs delivered by the yolk are likely imported in a free or RNP-associated form and subsequently reloaded into endogenous Argonautes within the embryonic cytosol. This model aligns with our observation of sequence-specific gene regulation by inherited miRNAs in embryos, underscoring a broader role for endosomal trafficking in miRNA-mediated intergenerational communication.

On the basis of these findings, we propose a model whereby maternal somatic miRNAs, stabilized within yolk granules, are released into the embryonic cytoplasm, loaded into Argonaute complexes and integrated into gene-regulatory networks. This defines a soma-to-germline signaling pathway with important implications for heritable stress adaptation.

Parallels can be drawn with emerging models of small RNA-based inheritance in mammals. Environmental exposures in mice have been shown to alter sperm miRNA content, which may influence offspring development^65–67^. These sperm miRNAs can originate from somatic epididymal cells^68^ or cytoplasmic droplets^69^; however, whether they contribute directly to the zygotic miRNA pool remains unclear. A similar transmission of miRNAs can occur during lactation^70^. While mammals have evolved postnatal lactation to deliver nutrients and immune factors, nonmammalian species such as *C*. *elegans* retain yolk-based provisioning^71^.

Remarkably, *C*. *elegans* exhibits a primitive form of lactation in which aged mothers vent yolk through the vulva after reproduction has ceased^72^. This secreted yolk nourishes developing larvae, analogous to milk in mammals. Notably, proteomic analysis of vented yolk has revealed the presence of several RNA-binding proteins and chaperones of miRNA regulation and silencing^30–35^, including CAR-1, VIG-1, PAB-1, CEY-2 and the HSP90–HSP70 complex, which we also detected in our proteomic analysis of internal yolk granules (Supplementary Table 1). We speculate that this secreted yolk may similarly carry miRNAs and associated RNPs, potentially influencing postembryonic development and physiology. This would represent a second window of maternal RNA-based regulation beyond embryogenesis. These findings raise intriguing questions about the evolutionary conservation of maternal RNA-based communication strategies, from invertebrate yolk secretion to mammalian milk^70,73^.

In conclusion, our study reveals a previously unrecognized route of intergenerational communication in which maternal intestinal miRNAs are packaged into the yolk, transported to the germline and functionally inherited by the embryo. These findings broaden our understanding of epigenetic inheritance, highlighting the yolk as a nexus for integrating environmental experience, maternal physiology and offspring development. This soma-to-germline communication through small RNAs opens up new avenues for exploring how organisms adapt across generations and how maternal environments influence the phenotype of their progeny.

## Methods

### *C*. *elegans* strains and maintenance

Strains were maintained at 20 °C using standard methods^74^. Bristol N2 was used as the wild-type reference strain. A complete list of strains is provided in Supplementary Table 7.

### Generation of CRISPR–Cas9 lines

Cas9–gRNA ribonucleoprotein complexes were microinjected into the hermaphrodite syncytial gonad^75^. gRNA design and synthesis were performed as described previously^76^. AID CRISPR–Cas9 tags were introduced using PCR-amplified double-stranded DNA (dsDNA) repair templates containing 30-bp homology arms and an AID::2×HA gBlock (Integrated DNA Technologies) sequence. Silent mutations were included to prevent recutting. Injected 10-µl mixes were prepared as described^77^ using the following final concentrations: 0.1 µg µl^−1^ Cas9–NLS protein (TrueCut V2, Invitrogen), 100 ng µl^−1^ in vitro transcribed target-gene gRNA, 80 ng µl^−1^ target-gene single-stranded DNA repair template or 300 ng μl^−1^ target-gene dsDNA repair template and 80 ng µl^−1^ pRF4 (roller marker). Cas9 and the target-gene gRNA were preincubated for 10–15 min at 37 °C before adding the other components to the mixture. dsDNA repair templates were subjected to a melting and annealing step^77^ before addition to the final mix. A complete list of gRNAs, repair templates and genotyping primers is provided in Supplementary Table 8.

### Depletion treatment for AID strains

Auxin (250 mM stock in ethanol) was added to NGM plates at a final concentration of 500 µM (0.2% ethanol control). Plates were seeded with *Escherichia* *coli* (strain OP50). Synchronized worms were transferred to auxin or control plates from the L3 stage unless otherwise indicated. For embryo collections on day 3, auxin treatment was initiated at day 1 of adulthood. For AID-2 experiments, 5-Ph-IAA (10 mM stock in ethanol) was used at 1 µM.

### Yolk purification and VIT-2::GFP sorting

Yolk granules marked by VIT-2::GFP were purified from RME-2-depleted worms (MHE210) (Extended Data Fig. 1c). Approximately 40,000 gravid adults were lysed in 1 ml of PBS supplemented with protease inhibitors and RNase inhibitor by douncing (20–25 strokes). Lysates were cleared by centrifugation at 1,000*g* to clear out the debris, followed by 18,000*g*. To enrich for yolk particles, which are lipoprotein complexes, the supernatant samples (supernatant 1) were labeled with the lipophilic dye DiD (5 µl ml^−1^, 37 °C for 5 min; Vybrant DiD). Labeled supernatants 1 were subjected to ultracentrifugation at 100,000*g* for 1 h. Pellets were washed twice in PBS (100,000*g* each) and resuspended in PBS (pellet 2). Supernatant samples after ultracentrifugation (supernatant 2) were diluted to about 10 ml, passed through a 23-gauge needle to disrupt aggregates and subjected to FACS sorting. Sorting was performed on a MoFlo Astrios (Beckman Coulter) using GFP and DiD signals to isolate yolk particles with a 70-μm nozzle at a pressure of 60 psi and a differential pressure with the sample of 0.3–0.4 psi. Wild-type N2 samples were used as negative controls, DiD-only samples as lipid-positive controls and VIT-2::GFP samples without DiD as GFP-positive controls to define sorting gates.

### Dot blot and western blot of VIT-2::GFP

VIT-2::GFP enrichment was assessed by dot blot and western blot. For dot blot analysis, equal volumes of input and fractionated samples were spotted onto nitrocellulose membranes, air-dried, blocked in 5% milk and probed with anti-GFP antibody. For western blotting, equal volumes of input and fractionated samples (supernatant 1, pellet 1, supernatant 2 and pellet 2; Extended Data Fig. 1c–e) were resolved on 4–12% Bis–Tris gels, transferred to membranes and immunoblotted using anti-GFP antibody (Chromotek). VIT-2::GFP was enriched in the pellet 2 fraction following ultracentrifugation.

### Quantification of VIT-2::GFP in two-cell embryos

VIT-2::GFP levels in two-cell embryos were quantified by fluorescence microscopy as a proxy for yolk loading. Embryos were obtained by alkaline hypochlorite treatment of synchronized gravid adults and kept on ice before imaging. Autofluorescence from wild-type N2 embryos was used as a control. Images were acquired on a Zeiss Axio Imager M2 microscope equipped with a PIXIS 1024 camera (Princeton Instruments) using a Plan-Apochromat ×40/1.40 objective.

### MS-based proteomic analysis of purified yolk

#### Sample preparation

Samples were dried with a speed vacuum device and resuspended in 50 µl of urea buffer (8 M urea and 200 mM ammonium bicarbonate) and reduced with 50 µl of 10 mM DTT, followed by incubation at 57 °C for 30 min. Samples were alkylated with the addition of 10 µl of 55 mM iodoacetamide, followed by incubation in the dark for 30 min. Proteins were digested with 0.4 µg of trypsin at 37 °C for 2 h and then another 0.4 µg of trypsin was added before incubating peptides overnight. Peptides were then dried, resuspended in 200 µl of 0.3% trifluoroacetic acid and purified using C18 StageTips before liquid chromatography (LC)–MS/MS analysis.

#### LC–MS/MS and data analysis

Peptides were analyzed using an RSLCnano system (Ultimate 3000, Thermo Scientific) coupled to an Orbitrap Exploris 480 MS instrument. Peptides were trapped on a C18 column (inner diameter: 75 μm, length: 2 cm; nanoViper Acclaim PepMap 100; Thermo Scientific) with buffer A (2% acetonitrile in H_2_O with 0.1% formic acid) at a flow rate of 3 µl min^−1^ over 4 min. Separation was performed on a 50 cm × 75 μm C18 column (nanoViper Acclaim PepMap RSLC, 2 μm, 100 Å; Thermo Scientific) regulated to a temperature of 50 °C with a linear gradient of 2% to 30% buffer B (100% acetonitrile in 0.1% formic acid) at a flow rate of 300 nl min^−1^ over 91 min. MS full scans were performed in the ultrahigh-field Orbitrap mass analyzer in range *m*/*z* 375–1,500 with a resolution of 120,000 at *m*/*z* 200. The top 20 most intense ions were subjected to Orbitrap for further fragmentation through high-energy collision dissociation activation and a resolution of 15,000 with the automatic gain control target set to 100%. We selected ions with charge state from 2+ to 6+ for screening. Normalized collision energy was set at 30 with dynamic exclusion of 40 s.

For identification, the data were searched against the *C*. *elegans* UP000001940 database (UniProt; downloaded November 2018) using Sequest HT through Proteome Discoverer (version 2.4). Enzyme specificity was set to trypsin and a maximum of two missed cleavage sites were allowed. Oxidized methionine, N-terminal acetylation, methionine loss and methionine acetylation loss were set as variable modifications. Maximum allowed mass deviation was set to 10 ppm for monoisotopic precursor ions and 0.02 Da for MS/MS peaks. The resulting files were further processed using myProMS (version 3.10)^78^. The false discovery rate (FDR) was calculated using Percolator^79^ and was set to 1% at the peptide level for the whole study.

### Stress assays

#### Oxidative stress assay

Embryos were collected by hypochlorite treatment from gravid adults (mothers on days 1–3, DP degron, RME-2 degron, *miR-235* knockout (KO) and *miR-85* KO) and ~30,000 embryos were plated on NGM plates containing paraquat (0.2, 0.3 or 0.4 mM; Sigma, 856177) seeded with *E*. *coli* OP50. After 72 h, worm development was quantified using a COPAS Biosorter. Gates were defined to exclude debris and dead eggs and to identify arrested L1 larvae on the basis of size. The percentage of worms progressing beyond L1 was calculated as described for the osmotic stress assay.

#### L1 starvation assay

Embryos from DP degron mothers were collected by hypochlorite treatment and maintained in M9 at ~7,000 worms per ml on a nutator for up to 14 days. At indicated time points (3, 8, 12 or 14 days), L1 larvae were plated (15,000 worms per plate) and grown for 48 h before analysis by COPAS Biosorter using the same gating strategy as above.

For developmental assays, stress exposure was performed as described above. For small RNA-seq and yolk imaging in two-cell embryos, as well as analysis of yolk persistence in embryos, hermaphrodite mothers were exposed to stress for 24 h from early L4 and embryos were collected by hypochlorite treatment.

#### Osmotic stress assay

Synchronized L1 larvae were grown for 48 h on control plates or auxin (DP degron) or 5-Ph-IAA (RME-2 degron). Worms were then transferred to plates containing 50 mM NaCl (control) or 300 mM NaCl (mild stress) with the corresponding treatment. After 24 h, adults were collected by sedimentation to avoid contamination from newly laid embryos and embryos were obtained by hypochlorite treatment. Embryos were plated on NGM plates containing 500 mM NaCl (high osmotic stress) and grown for 64 h.

Development was assessed using a COPAS Biosorter. Gate 1 included all worms (excluding debris and dead eggs) and gate 2 identified worms progressing beyond the L1 stage, as defined previously^55^. The percentage of worms passing L1 was calculated as (gate 2/gate 1) × 100. At least 2,000 worms were analyzed per condition.

### Larval development analysis with COPAS Biosorter

Larval development was quantified using a COPAS Biosorter (Union Biometrica) on the basis of optical density and time of flight. Gates were defined for appropriate developmental stages and validated by differential interference contrast microscopy of sorted samples.

### Brood size assays

Synchronized L1 larvae were grown to the L3 stage and individually transferred to ethanol or auxin plates (DP degron) or control plates (wild type). Worms were transferred to fresh plates three times and brood size was calculated as the total number of embryos and larvae produced over 4 days.

### Small RNA extraction and library preparation from sorted and unsorted yolk granules

Unsorted or FACS-sorted VIT-2::GFP fractions were frozen in dry ice with TRIzol Reagent (Invitrogen) for small RNA extraction. After five repetitions of freeze and thaw, RNA was isolated according to the manufacturer’s instructions. The small RNA library preparation was performed essentially as described previously^76^. Amplified libraries were multiplexed to purify further using PippinPrep DNA size selection with 3% gel cassettes and the following parameters for size selection: BP start (133) and BP end (155). The purified libraries were quantified using the Qubit fluorometer high-sensitivity dsDNA assay kit (Thermo Fisher Scientific, Q32851) and sequenced on Illumina NextSeq 2000 platform.

### Collection and RNA extraction of L1 worms

Synchronized nonstarved L1 larvae were obtained using a NemaSync *C*. *elegans* synchronizer (model 5000). Larvae were flash-frozen in TRIzol (Invitrogen) and, after six repetitions of freeze and thaw, the RNA was extracted using the Direct-zol RNA Microprep kit (ZymoResearch, R2062). Small RNAs (17–200 nt) and large RNAs (>200 nt) were separated using the Quick-RNA MicroPrep kit (ZymoResearch, R1051) to sequence both the small RNA and the mRNA from the same samples. The large RNA fractions were treated with DNase. RNA integrity was assessed using an Agilent TapeStation and samples with RNA integrity number (RIN) > 8 were used for RNA-seq.

### Strand-specific RNA-seq library preparation

DNase-treated total RNA with RIN > 8 was used to prepare strand-specific RNA libraries. Ribosomal and mitochondrial ribosomal RNAs (rRNAs) were depleted using a custom RNAse-H-based method to degrade rRNAs using complementary oligos as described previously^76^. Strand-specific RNA libraries were prepared using at least 100 ng of rRNA-depleted RNAs using the NEBNext Ultra II directional RNA library prep kit for Illumina (E7760S). RNA libraries were analyzed on the Agilent 2200 TapeStation system using high-sensitivity D1000 screentapes and quantified using the Qubit fluorometer high-sensitivity dsDNA assay kit (Thermo Fisher Scientific, Q32851). Multiplexed libraries were sequenced on a NextSeq 2000 Illumina platform.

### Small RNA-seq library preparation from manually picked two-cell embryos

Precisely staged two-cell embryos were obtained by alkaline hypochlorite treatment of synchronized gravid adult hermaphrodites and 50 two-cell embryos were collected using a mouth pipette. Embryos were lysed for 10 min at 65 °C and 1 min at 85 °C in a lysis solution containing Tris-Cl pH 8, 5 mM final, EDTA 0.25 mM final, Triton X-100 0.5%, Tween-20 0.5% and proteinase K 0.5%. The lysates were subjected to RNA purification using the Quick-RNA MicroPrep kit (ZymoResearch, R1051) and small RNAs (RNA fraction: 17–200 nt) and mRNAs (RNA fraction > 200 nt) were collected separately. A small RNA spike-in was added before embryo lysis. For this, we used four synthetic miRNAs at four different concentrations:

Spike 1: GAGAGCAGUGGCUGGUUGAGAU at 100 μM

Spike 2: GUUGAUAGAAGCUAUAGCAUGC at 10 μM

Spike 3: GUGCUGAGAUCGUUAGACUAAC at 1 μM

Spike 4: CUCAUCGAUACCAUUGCUAGAG at 0.1 μM

A final 0.33 nM spike-in miRNA pool was used for each embryo collection. Next, the library preparation was performed essentially as described previously^76^.

To prevent the cloning of six highly abundant rRNAs, which otherwise dominate the sequenced library, we preannealed six locked LNA oligos complementary to these rRNAs before 5′ adaptor ligation and reverse transcription. This approach prevents the ligation of the 5′ adaptor to these rRNAs as described previously^80^. The sequences of the oligonucleotide used were as follows:

GACTGAGTTCAGGTTGAGA/3SpC3/

GCTGACAGAATCAATCAGGTA/3SpC3/

CGATGATCCAGCTGCAGGTTCA/3SpC3/

AGCTTACAACATCCAGGATTCCCA/3SpC3/ AACCGATCCATCGCTGAAGCTA/3SpC3/

AAACAACCCTGAACCAGACGT/3SpC3/

Amplified libraries were multiplexed to purify further using PippinPrep DNA size selection with 3% gel cassettes. Two rounds of size selection were performed using the following parameters: first selection, BP start (127) and BP end (156); second selection, BP start (133) and BP end (153). The purified libraries were analyzed and quantified on the Agilent 2200 TapeStation system using high-sensitivity D1000 screentapes and sequenced on a NextSeq 2000 Illumina platform.

### Small RNA-seq library preparation from young adult worms

Young adult worms were collected at 48 h after hatching and thoroughly washed; then, TRIzol reagent (Invitrogen) was added to the worm pellet for small RNA extraction. After five repetitions of freeze and thaw, RNA was isolated according to the manufacturer’s instructions. Small RNAs (RNA fraction: 17–200 nt) were separated from the mRNAs (RNA fraction > 200 nt) fraction using the Quick-RNA MicroPrep kit (ZymoResearch, R1051). The small RNA library preparation was performed essentially as described previously^76^. Amplified libraries were multiplexed and further purified using PippinPrep DNA size selection with 3% gel cassettes. The following parameters were used for size selection: BP start (133) and BP end (153). The purified libraries were quantified using the Qubit fluorometer high-sensitivity dsDNA assay kit (Thermo Fisher Scientific, Q32851) and sequenced on an Illumina NextSeq 2000 platform.

### Immunostaining

Immunostaining was performed as described previously^76^. The worms were permeabilized on slides coated with 0.1% poly(L-lysine) solution (Sigma, P4832-50mL) by freeze-cracking as described previously^81^. The primary antibody, anti-Flag (Sigma, F1804), at a dilution of 1:500, was incubated overnight at 4 °C in PBS, 0.1% Tween-20 and 5% BSA. The secondary antibody, anti-mouse (Invitrogen, Cy3), was incubated at a dilution of 1:500 for 4 h at room temperature. DNA was stained with DAPI (antifade mounting medium with DAPI, Vectashield, H-1200).

### Progeny collection from the heterozygote *miR-85* mutant

To distinguish progeny that inherit maternal *miR-85* without zygotic expression, we used a GFP balancer strategy^82^. *miR-85(n4117)* mutants were crossed to mIn1 [mIs14 [myo-2::gfp; pes-10::gfp]; dpy-10(e128)] II, which provides dose-dependent GFP expression. L1 progeny from heterozygous mothers were sorted by COPAS based on fluorescence intensity to isolate homozygous mutants (no GFP) and heterozygotes (intermediate GFP). Sorted worms were grown to L4 and exposed to control (50 mM NaCl) or osmotic stress (300 mM NaCl). Progeny were resorted to obtain (1) homozygous mutants from heterozygous mothers (maternal inheritance only, P_0_hetG_0_KO); (2) heterozygotes (maternal + zygotic expression, P_0_hetG_0_het); and (3) homozygous mutants from homozygous mothers (no inheritance). Sorted L1 larvae were collected in TRIzol for RNA and small RNA-seq.

### Sequencing data analysis

All the sequencing data were demultiplexed with Illumina bcl2fastq converter (version 2.17.1.14) and quality control was performed with fastQC (version 0.11.5).

#### RNA-seq analysis

HISAT2 (version 2.0.4)^83^ was used for mapping RNA-seq reads aligned to the *C*. *elegans* genome sequence (ce11, *C*. *elegans* Sequencing Consortium WBcel235). After alignment, reads mapping to annotated protein-coding genes were counted using featureCounts (version 2.0.1)^84^. Counted reads for protein-coding genes were used for differential expression analysis using the R/Bioconductor package DESeq2 (version 1.26.0)^85^.

#### Small RNA-seq analysis

The analysis of small RNA-seq on RNA extracted from the purified yolk, unsorted fraction and total RNAs was performed as previously described^76^. Yolk-enriched miRNAs were identified using Limma^86^ with the limma-voom approach^87^. We selected miRNAs having log_2_ fold change ≥ 0.5 and *P* value ≤ 0.05.

#### Spike-in miRNA-seq analysis from two-cell embryos

After demultiplexing, the 3′ adaptor was trimmed from raw reads using Cutadapt (version 1.15)^88^ with the parameter ‘-O 5’ and the adaptor (TGGAATTCTCGGGTGCCAAGG) given with option -a. Four randomized nucleotides were trimmed at both ends (option: --cut 4 --cut −4). The selected 18–26-nt reads were aligned to the *C*. *elegans* genome sequence (ce11, *C*. *elegans* Sequencing Consortium WBcel235) using Bowtie2 (version 2.3.4.1)^89^ with the following parameters:‘ -L 6 -N 0 -i S,1,0.8 --no-1mm-upfront --score-min L,0,0 --ignore-quals --no-unal’. Aligned reads mapping to annotated genomic features (miRNAs and piRNAs) were then counted using featureCounts (version 2.0.1)^84^ with the stranded option (-s 1). Counts were normalized in each sample using spike-in counts (scaling factor per sample = 10^3^ × total_nb_spikes_counted/total_nb_reads_aligned) in a dataset. Scaling factors were then generally ‘minimized’; all scaling factors of the dataset were divided by the minor scaling factor in the dataset. Differential expression analysis of miRNAs was performed using Limma^86^.

#### Tissue enrichment and Gene Ontology enrichment analyses

Upregulated and downregulated genes identified in RNA-seq experiments using embryos and larvae from DP degron were analyzed for their tissue and Gene Ontology enrichments as described previously^40^.

### Statistics and reproducibility

Almost all the experiments shown in this study were performed independently at least twice and no inconsistent results were observed. Most of the graphs were generated using GraphPad Prism 10. The log fold changes for all the plots were calculated using the mean of biologically independent replicates. Details of the statistical analyses used, precise *P* values, statistical significance and sample sizes for all graphs are provided in the figure legends.

### Reporting summary

Further information on research design is available in the Nature Portfolio Reporting Summary linked to this article.

## Online content

Any methods, additional references, Nature Portfolio reporting summaries, source data, extended data, supplementary information, acknowledgements, peer review information; details of author contributions and competing interests; and statements of data and code availability are available at 10.1038/s41594-026-01816-5.

## Supplementary information

Supplementary Information Supplementary Figs. 1 and 2.

Reporting Summary

Peer Review File

Supplementary Table Supplementary Table 1: Identified proteins from purified yolk granules by MS-based proteomics. Supplementary Table 2: miRNAs enriched in yolk. Supplementary Table 4: Differentially expressed protein-coding genes in L1 larvae RNA-seq experiments. Supplementary Table 7: Strain list generated or used in this study. Supplementary Table 8: CRISPR–Cas9 guide RNA lists used in this study.

Supplementary Table 3 Differentially expressed miRNAs in two-cell embryos from yolk-depleted mothers, intestinal DP-depleted young adults and two-cell embryos and two-cell embryos from wild-type mothers on day 1 to day 3.

Supplementary Table 5 Differentially expressed miRNAs in two-cell embryos from wild-type and DP degron strain mothers exposed to osmotic stress or starvation.

Supplementary Table 6 Differentially expressed mRNAs in L1 larvae from DP degron strain mothers exposed to osmotic stress and *miR-85* mutants mRNAs and miRNAs.

Supplementary Data 1 Source data for Supplementary Fig. 1.

Supplementary Data 2 Source data for Supplementary Fig. 2.

## Source data

Source Data Fig. 1 Statistical source data.

Source Data Fig. 2 Statistical source data.

Source Data Fig. 3 Statistical source data.

Source Data Fig. 4 Statistical source data.

Source Data Fig. 5 Statistical source data.

Source Data Fig. 6 Statistical source data.

Source Data Fig. 7 Statistical source data.

Source Data Extended Data Fig.1 Statistical source data.

Source Data Extended Data Fig.2 Statistical source data.

Source Data Extended Data Fig.3 Statistical source data.

Source Data Extended Data Fig.5 Statistical source data.

Source Data Extended Data Fig.6 Statistical source data.

Source Data Extended Data Fig.7 Statistical source data.

Source Data Extended Data Fig.8 Statistical source data.

Source Data Extended Data Fig.9 Statistical source data.

Source Data Extended Data Fig.10 Statistical source data.

Source Data Extended Data Fig.1 Unprocessed western blots.

## Data Availability

All the sequencing data are available at the following accession numbers GSE261340 and GSE261341. The MS proteomics data were deposited to the ProteomeXchange Consortium through the PRIDE partner repository with the dataset identifier PXD064947. Source data are provided with this paper.

## Code availability

Custom code and data analysis workflows are available from GitLab (https://gitlab.pasteur.fr/bli/bioinfo_utils). The spike-in miRNA sequencing pipeline is available from GitHub (https://github.com/achervov/C.elegans_miRNAseq_pipeline).
