# Supplementary material for: Soma-to-germline miRNA inheritance through yolk promotes stress resilience in progeny
Source: Nat Struct Mol Biol. 2026 May 22;33(6):985–97. doi: 10.1038/s41594-026-01816-5 (PMC13275319; doi:10.1038/s41594-026-01816-5)
Supplement: Supplementary file 1 — Supplementary Figs. 1 and 2. [file 41594_2026_1816_MOESM1_ESM.pdf]

nature structural & molecular biology

Supplementary information https://doi.org/10.1038/s41594-026-01816-5

Soma-to-germline miRNA inheritance through yolk promotes stress resilience in progeny

In the format provided by the authors and unedited

Supplemental Figure 1

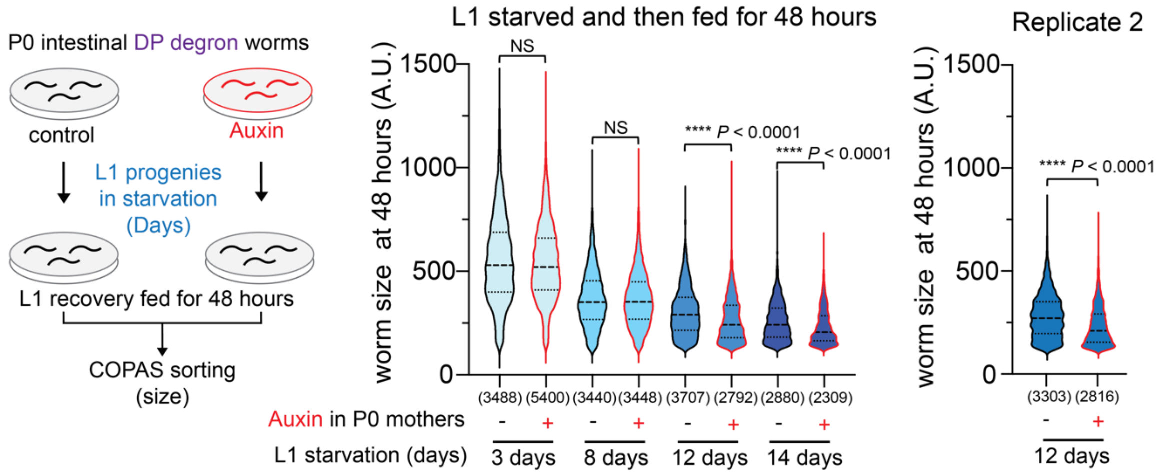

Supplementary Figure 1: Yolk-enriched miRNAs contribute to progeny recovery after prolonged L1 starvation. a, Left: Schematic of the experimental design. L1 progeny derived from DP degron mothers grown with or without auxin from the L3 stage were subjected to prolonged L1 starvation for the indicated durations (3, 8, 12, or 14 days), followed by recovery on food for 48 h. Larval size was then quantified by COPAS sorting. Right: Violin plots showing larval size after 48 h of recovery feeding for progeny derived from control (– auxin) or intestinal DP-depleted (+ auxin) mothers, following different durations of L1 starvation. A second independent biological replicate is shown on the right for the 12-day starvation condition. Dashed and dotted lines indicate median and quartiles, respectively. Statistical analysis was performed using two-tailed Mann–Whitney–Wilcoxon tests. ****$P < 0.0001$; NS, not significant. Sample sizes (worms) are shown in parentheses.

Supplemental Figure 2

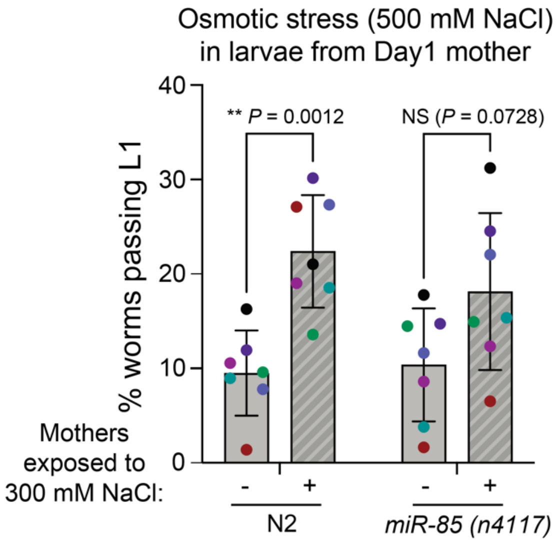

Supplementary Figure 2: Contribution of *miR-85* to inherited osmotic stress resilience. Percentage of L1 larvae progressing beyond the L1 stage after exposure to high osmotic stress (500 mM NaCl), derived from Day 1 wild-type (N2) or *mir-85* (*n4117*) knockout mothers. P0 mothers were either maintained under control conditions (50 mM NaCl; solid bars) or exposed to mild osmotic stress (300 mM NaCl; striped bars) prior to egg laying. Each dot represents an independent biological replicate (n = 7). Bars indicate mean ± SD. Statistical analysis was performed using two-tailed Mann–Whitney–Wilcoxon tests. **$P = 0.0012$; NS, not significant.
