# Supplementary material for: Soma-to-germline miRNA inheritance through yolk promotes stress resilience in progeny
Source: Nat Struct Mol Biol. 2026 May 22;33(6):985–97. doi: 10.1038/s41594-026-01816-5 (PMC13275319; doi:10.1038/s41594-026-01816-5)
Supplement: Supplementary file 2 — Reporting Summary [file 41594_2026_1816_MOESM2_ESM.pdf]

nature portfolio

Corresponding author(s): Germano Cecere
Last updated by author(s): Apr 9, 2026

Reporting Summary

Nature Portfolio wishes to improve the reproducibility of the work that we publish. This form provides structure for consistency and transparency in reporting. For further information on Nature Portfolio policies, see our Editorial Policies and the Editorial Policy Checklist.

Statistics

For all statistical analyses, confirm that the following items are present in the figure legend, table legend, main text, or Methods section.

n/a	Confirmed	
☐	☒	The exact sample size (*n*) for each experimental group/condition, given as a discrete number and unit of measurement
☐	☒	A statement on whether measurements were taken from distinct samples or whether the same sample was measured repeatedly
☐	☒	The statistical test(s) used AND whether they are one- or two-sided<br>*Only common tests should be described solely by name; describe more complex techniques in the Methods section.*
☒	☐	A description of all covariates tested
☒	☐	A description of any assumptions or corrections, such as tests of normality and adjustment for multiple comparisons
☐	☒	A full description of the statistical parameters including central tendency (e.g. means) or other basic estimates (e.g. regression coefficient) AND variation (e.g. standard deviation) or associated estimates of uncertainty (e.g. confidence intervals)
☐	☒	For null hypothesis testing, the test statistic (e.g. *F*, *t*, *r*) with confidence intervals, effect sizes, degrees of freedom and *P* value noted<br>*Give P values as exact values whenever suitable.*
☒	☐	For Bayesian analysis, information on the choice of priors and Markov chain Monte Carlo settings
☒	☐	For hierarchical and complex designs, identification of the appropriate level for tests and full reporting of outcomes
☐	☒	Estimates of effect sizes (e.g. Cohen's *d*, Pearson's *r*), indicating how they were calculated

Our web collection on statistics for biologists contains articles on many of the points above.

Software and code

Policy information about availability of computer code

Data collection

Illumina NextSeq 2000 platform was used for sequencing
Fluorescnece images were taken on Zeiss Axio Imager M2 and acquired using Meta Morph

Data analysis

RNA-seq analysis:
- Demultiplexing with Illumina bcl2fastq converter version v2.17.1.14
- Quality control with fastQC version v0.11.5

Allignment with HISAT2 version 2.0.4 (RNAseq)
Bowtie2 v.2.3.4.1 (sRNA-seq)
- Genomic features annotation with featureCounts version 1.5.2
- Differential gene expression analysis with DESeq2 version 1.20.0, Limma,

Small RNA-seq, RNA-seq analyses:
- Demultiplexing with Illumina bcl2fastq converter version v2.17.1.14
- Quality control with fastQC version v0.11.5
- 3' adaptor trimming with Cutadapt version 1.15
- Allignment with Bowtie2 version 2.3.4.1
- Metaprofile with deeptools package version 3.1.2
- gffutils
- Python 3.9
- R/Bioconductor package DESeq2 (version 1.26.0)

April 2023

Distance mapping
-Scipy library (version 1.3.2)
- Matplotlib version 3.1.1

Custom code and data analysis workflows are available at https://gitlab.pasteur.fr/bli/bioinfo_utils. The spike-in miRNA sequencing pipeline is available at https://github.com/achervov/C.elegans_miRNAseq_pipeline. Statistical analysis for most plots was done using GraphPad Prism 10.

MS/MS data analysis
- Sequest HT through Proteome Discoverer (v.2.2)
- myProMS v.3.9
- MassChroQ v.2.2.1
- Database C.elegans (CAEEL) UP000001940 database

Images were processed using ImageJ software V2.0.0.

For manuscripts utilizing custom algorithms or software that are central to the research but not yet described in published literature, software must be made available to editors and reviewers. We strongly encourage code deposition in a community repository (e.g. GitHub). See the Nature Portfolio guidelines for submitting code & software for further information.

Data

Policy information about availability of data

All manuscripts must include a data availability statement. This statement should provide the following information, where applicable:

- Accession codes, unique identifiers, or web links for publicly available datasets
- A description of any restrictions on data availability
- For clinical datasets or third party data, please ensure that the statement adheres to our policy

Data availability
All the sequencing data are available at the following accession numbers GSE261340 and GSE261341. The MS proteomics data have been deposited to the ProteomeXchange Consortium via the PRIDE partner repository with the dataset identifier PXD064947.

Code availability
Custom code and data analysis workflows are available at https://gitlab.pasteur.fr/bli/bioinfo_utils. The spike-in miRNA sequencing pipeline is available at https://github.com/achervov/C.elegans_miRNAseq_pipeline

Research involving human participants, their data, or biological material

Policy information about studies with human participants or human data. See also policy information about sex, gender (identity/presentation), and sexual orientation and race, ethnicity and racism.

Reporting on sex and gender	not applicable
Reporting on race, ethnicity, or other socially relevant groupings	not applicable
Population characteristics	not applicable
Recruitment	not applicable
Ethics oversight	not applicable

Note that full information on the approval of the study protocol must also be provided in the manuscript.

Field-specific reporting

Please select the one below that is the best fit for your research. If you are not sure, read the appropriate sections before making your selection.

☒ Life sciences ☐ Behavioural & social sciences ☐ Ecological, evolutionary & environmental sciences

For a reference copy of the document with all sections, see nature.com/documents/nr-reporting-summary-flat.pdf

Life sciences study design

All studies must disclose on these points even when the disclosure is negative.

Sample size	50 2-cell embryos or thousands L1 worms were used for RNA-seq and total sRNA-seq. This sample size has been empirically evaluated to be sufficient.<br>For yolk purification 40,000 gravid adults were taken. Multiple replicate (greater than 3 were) were performed for sorting.

Data exclusions	no data exclusion has been applied.
Replication	All the experiments shown in this study were performed independently at least two times and no inconsistent results were observed. All attempts at replication were successful.
Randomization	none
Blinding	none

Reporting for specific materials, systems and methods

We require information from authors about some types of materials, experimental systems and methods used in many studies. Here, indicate whether each material, system or method listed is relevant to your study. If you are not sure if a list item applies to your research, read the appropriate section before selecting a response.

Materials & experimental systems

n/a	Involved in the study
☐	☒ Antibodies
☒	☐ Eukaryotic cell lines
☒	☐ Palaeontology and archaeology
☐	☒ Animals and other organisms
☒	☐ Clinical data
☒	☐ Dual use research of concern
☒	☐ Plants

Methods

n/a	Involved in the study
☒	☐ ChIP-seq
☐	☒ Flow cytometry
☒	☐ MRI-based neuroimaging

Antibodies

Antibodies used	α-FLAG antibody (F3165, Sigma) dilution used 1:1000,<br>α-GFP (Chromotek), dilution used 1:1000,<br>HRP conjugated anti-rabbit (31460, Pierce) dilution used 1:10000<br>HRP conjugated anti-mouse (31430, Pierce) dilution used 1:10000
Validation	anti-FLAG antibody (F3165, Sigma) has been validated by vendor (https://www.sigmaaldrich.com/cata1og/product/sigma/f3165).<br>anti-GFP antibody has been validated by vendor (https://www.ptglab.com/products/pictures/pdf/Manual_GFP_PABG1.pdf)<br>HRP conjugated anti-rabbit (31460, Pierce) ,validated by manufacturer<br>HRP conjugated anti-mouse (31430, Pierce), validated by manufacturer

Animals and other research organisms

Policy information about studies involving animals; ARRIVE guidelines recommended for reporting animal research, and Sex and Gender in Research

Laboratory animals	All the data collected for this study derived from hermaphrodite Caenorhabditis elegans nematode culture.
Wild animals	No wild animals has been used in this study
Reporting on sex	none
Field-collected samples	This study did not involve samples collected from the field.
Ethics oversight	No ethical approval was required.

Note that full information on the approval of the study protocol must also be provided in the manuscript.

Plants

Seed stocks	not applicable
Novel plant genotypes	not applicable
Authentication	not applicable

Flow Cytometry

Plots

Confirm that:

☒ The axis labels state the marker and fluorochrome used (e.g. CD4-FITC).

☒ The axis scales are clearly visible. Include numbers along axes only for bottom left plot of group (a 'group' is an analysis of identical markers).

☒ All plots are contour plots with outliers or pseudocolor plots.

☒ A numerical value for number of cells or percentage (with statistics) is provided.

Methodology

Sample preparation	We purified yolk granules marked by VIT-2::GFP from RME-2 depleted worms (MHE210). Briefly, 40,000 gravid adults were lysed in 1 mL 1x PBS supplemented with 1x Halt protease inhibitor and 40 U/mL Ribolock by douncing in a metal douncer by 20-25 strokes. Lysate was centrifuged at 1000g to clear out the debris. The supernatant was further centrifuged at 18000g. Considering the yolk is a lipo-protein complex, therefore, in addition to sorting for GFP-tagged VIT-2 protein, we also used the parameter of positive staining for 1,1'-Dioctadecyl-3,3,3',3'-tetramethylindocarbo-cyanine (DiD). DiD is a highly lipophilic dye that we used to stain the lipid component of yolk particles. Post centrifugation, supernatant (supernatant 1) was collected, and DiD labeling to label lipids of yolk particles was performed at 37°C for 5 min using 5 µL/mL Vybrant™ DiD Cell-Labeling Solution. After labeling, supernatant 1 was centrifuged at 100,000g for 1 hour. After ultracentrifugation, the supernatant (supernatant 2) was collected and kept for downstream analysis. The pellet was washed twice with 1x PBS, followed by 100,000g of spin each time. After the final wash, the pellet (Pellet 2) was resuspended in 1 mL of prechilled 1x PBS. The sample was further diluted to about 10 mL. The sample was passed through a 23-gauge needle to break large aggregates.
Instrument	MoFlo Astrios EQ
Software	Software for sorting: Summit V 6.3.1.16845<br>Software to prepare panels for figure 1f: Floreada.io
Cell population abundance	Population of GFP-positive and DiD-positive yolk particles varied between 0.05 % to 1 %. This proportion varied between biological replicates based on lysis efficiency.
Gating strategy	The GFP was excited with the 488 nm laser at 200mW and detected with a 513/26 filter. The DiD was excited with the 640nm laser at 100 mW and detected with a 671/30 filter. Samples prepared from the wild-type N2 strain (negative for GFP) were used as a no–stain control; samples prepared from the wild-type N2 strain and stained only for DiD (negative for GFP) were used as DiD-positive samples. Samples prepared from vit-2:gfp; AID:rme-2 and not stained for DiD were used as GFP-positive samples to design sorting gates. Gating was performed in the following hierarchy FSC/SSC (in log scale) > Single particles (FSC-A/FSC-H) > GFP positive particles (GFP/SSC) > DiD positive particles (DiD/SSC)

☒ Tick this box to confirm that a figure exemplifying the gating strategy is provided in the Supplementary Information.
