# Supplementary figures and images for: Soma-to-germline miRNA inheritance through yolk promotes stress resilience in progeny
Source: Nat Struct Mol Biol. 2026 May 22;33(6):985–97. doi: 10.1038/s41594-026-01816-5 (PMC13275319; doi:10.1038/s41594-026-01816-5)

Uncropped raw blot images

Extended Figure 1d

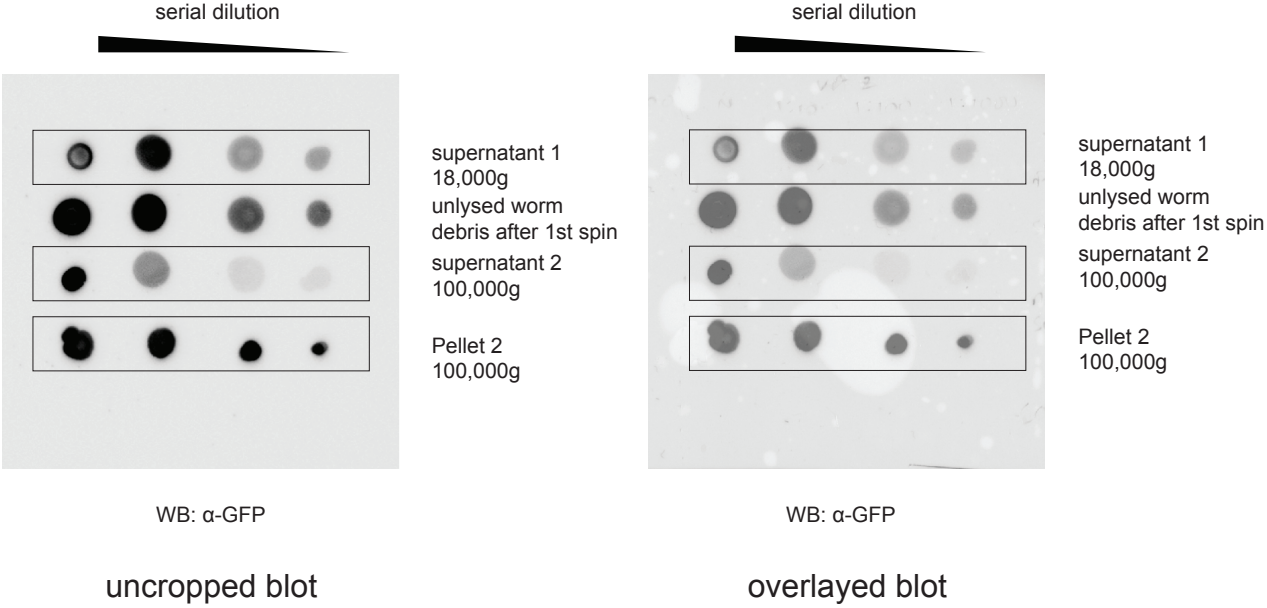

Extended Figure 1e

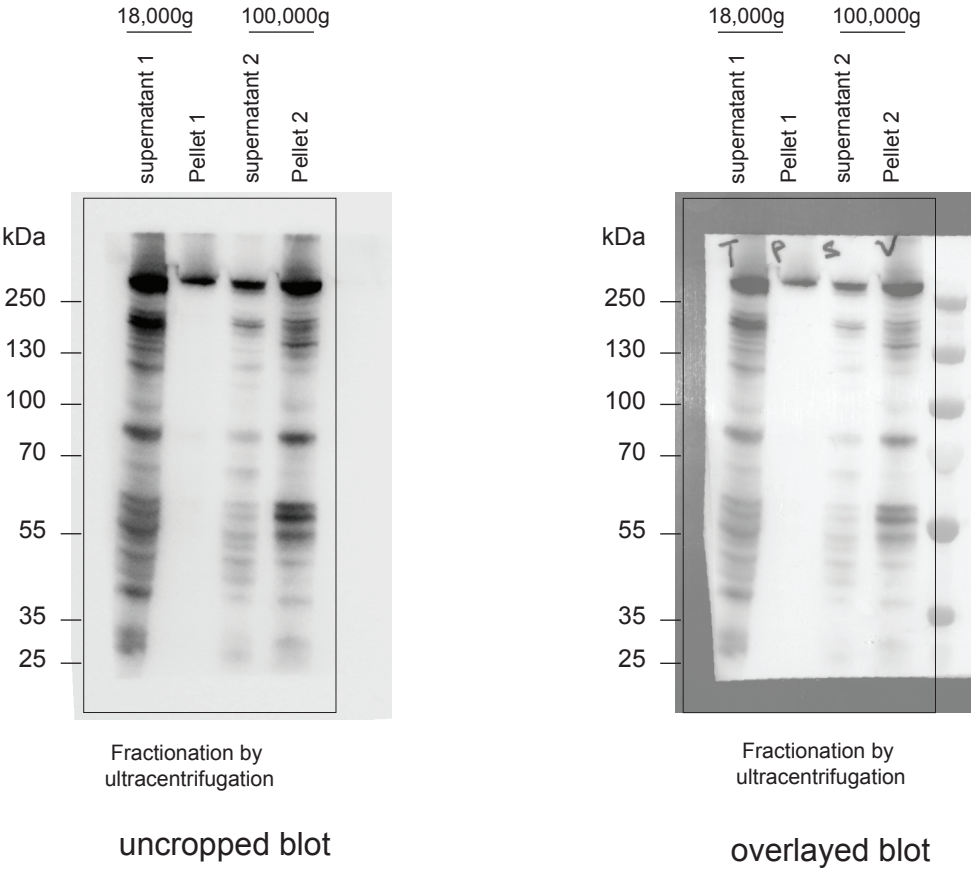

Supplement: Supplementary file 26 — Unprocessed western blots. [file 41594_2026_1816_MOESM26_ESM.pdf]
