# Supplementary material for: Soma-to-germline miRNA inheritance through yolk promotes stress resilience in progeny
Source: Nat Struct Mol Biol. 2026 May 22;33(6):985–97. doi: 10.1038/s41594-026-01816-5 (PMC13275319; doi:10.1038/s41594-026-01816-5)
Supplement: Supplementary file 3 — Peer Review File [file 41594_2026_1816_MOESM3_ESM.pdf]

nature portfolio

Peer Review File

Soma-to-Germline miRNA inheritance via Yolk Promotes Stress Resilience in Progeny

Corresponding Author: Dr Germano Cecere

Version 0:

Decision Letter:

Dear Dr. Cecere,

Thank you for your patience and for submitting your manuscript, "Soma-to-Germline miRNA inheritance via Yolk Promotes Stress Resilience in Progeny". The comments of 5 expert referees are below. Please note that Reviewers #3-4 co-reviewed the manuscript, so their comments are identical.

While we recognize that Reviewer #2 finds the work to be of particular interest, the reviewers have collectively raised serious concerns about the functional consequences of parental miRNA transport in progeny (as best outlined by Reviewers #1 and #5). Based on these comments, we cannot offer to publish the study in Nature Structural & Molecular Biology.

I am sorry we could not be more positive on this occasion, though we hope the referees' comments will be useful to you in revising the manuscript for submission elsewhere. We are also willing to consult with other editorial teams within the Nature Portfolio, should you be interested in transferring the manuscript to another title for further consideration.

Best regards,

George Inglis

George Inglis, PhD
Senior Editor
>a href="https://www.nature.com/nsmb/research-cross-journal-editorial-team"<Research Cross-Journal Editorial Team>/a<
>i<Nature Structural & Molecular Biology>/i<

Reviewer Expertise:

Reviewer #1: RNAi in C. elegans, transgenerational inheritance

Reviewer #2: piRNAs in C. elegans

Reviewers #3-4: miRNAs in C. elegans development

Reviewer #5: DNA damage and aging in C. elegans

Reviewer #1 (Remarks to the Author):

The authors present the isolation of yolk from C. elegans and the analysis of the impact that RNAs in yolk can have on the development of progeny. They identify a set of miRNAs that are claimed to be transported along with yolk from the intestine to progeny. They use Auxin-induced degrons for conditional depletion of the yolk receptor in the germline and the miRNA processing enzymes in the intestine. Sequencing of 2-cell embryos is used to select against miRNAs that could be transcribed early in the zygote. They also present assays for stress tolerance in progeny correlated with parental experience.

A major strength of the paper is the biochemical isolation of yolk and its analysis, which is a technical challenge. However, there are weaknesses that make the central claims of the paper ultimately unsupported. The biggest concern is that there is no direct evidence that the micro RNAs from the parental intestine are imported into the cytosol in progeny and that they function specifically to cause the observed changes in gene expression. Experiments addressing what is required in addition to initial uptake via RME-2, particularly for crossing the endomembranes into the cytosol, will strengthen the claims

in the paper. Additionally, making mutations in miRNAs in parents and compensatory mutations in targets that are introduced only in progeny by crossing could provide the compelling evidence needed for the claim of inherited miRNAs functioning in progeny (TargetScan predictions are inadequate for this key claim). Finally, in explaining changes in gene expression, alternative hypotheses are not adequately considered.

In short, there is evidence for presence of parental miRNAs in yolk, but not for their functioning in progeny. Therefore, the title of the paper is unsupported.

Major comments:
1. It appears that the miRNAs are detected in their single-stranded form (e.g., abundances in Fig. 1e). If that is the case, how is their transport across membranes after endocytosis envisioned? Minimally, some discussion of this aspect will be useful.
2. The changes in gene expression caused by the miRNA depletion is relatively modest <~4-fold in all cases for the 63 genes affected (Fig. 2f). Indirect effects that could result in these changes have not been excluded. For example, when miRNA production is disrupted in the parental intestine, what evidence supports the changes in these genes being the result of the import of miRNA into the progeny and not disruption of anything else that is also transported along with yolk. One hypothesis could be that the maternal deposition of heme is impaired, which could explain why heme responsive genes (hrg-1 and hrg-7 highlighted in Fig. 2f are altered). Note that Drosha and Pasha need heme for proper functon(Partin et al., Nature Communications, 2017), which is further suggestive of heme-related feedback.
3. The effect of paraquat on development appears modest (Fig. 4a). Given the spread of the data, what would an effect size calculation reveal? Given the possibility of alternative ways that the disruption of miRNA production in the intestine could impact the next generation, even observing a large effect size does not guarantee the effect is because of miRNAs that are transported from parent to progeny. A similar concern remains when considering the responses to osmotic stress. The <2-fold changes with large errors observed to be dependent on RME-2 and Drosha/Pasha in the parental intestine could be indirect – not dependent on miRNA entry into the cytosol in progeny.

Minor comments:
1. While it is noted that there were no 'global enrichments' of piRNAs or 22G RNAs in the sorted yolk fraction, were there gene-specific enrichments or depletions observed? Knowing this would be important for understanding the relative roles of miRNAs and other small RNAs, if any.
2. The cutoff for defining yolk-enriched miRNAs seems a little low (log2 fold change of 0.5 instead of the more common 1 or 2, as indeed used for RNA-seq later in the paper).

Reviewer #2 (Remarks to the Author):

In this study, Auperin et al. investigate the roles of yolk-inherited miRNAs in the stress resilience of progeny in C. elegans. In my opinion, this is a high-impact paper that will open a new field for further investigation into the roles of yolk-carrying small RNA in intergenerational gene regulation. Many of the experiments, including small RNA/mRNA sequencing and the use of an auxin-induced system, are elegantly designed and carefully executed to demonstrate the roles of yolk-carrying miRNA in stress responses of the progeny. Assuming the authors are able to address some of the critiques listed below, I see this manuscript as one of the breakthrough papers in the field of small RNAs.

Main Critiques:
1. The authors use a combination of centrifugation and sorting to isolate GFP::VIT granules, which was considered as yolk granules. Nonetheless, as C. elegans gut granules are known to exhibit autofluorescent signals, such purification will likely contain contamination from gut granules, which should be acknowledged in the text. They also did not show Western blot data or other data, with both positive and negative controls, to demonstrate the effectiveness of GFP sorting (comparing the sorted vs. unsorted signals).

2. Related to the issue above, there is little description regarding the background of yolk granules. For example, what is known about the stability and function of yolk granules, and how were they purified in previous studies? In addition, if the authors are purifying yolk granules, the description in the abstract should change from "purified the yolk" to "purified the yolk granules."

3. miRNA production is tightly regulated to precisely control the expression of its target gene during development, such as in the case of lin-4 miRNA. Therefore, it is important to describe the abundance, not just the enrichment, of yolk-enriched miRNAs in the embryos. For example, it would be valuable to compare the abundance of yolk-enriched miRNAs to other germline-enriched miRNAs (such as the mir-35 families) that are inherited through the cytoplasm of the oocyte/sperm.

4. Are yolk-enriched miRNAs loaded with Argonautes? It would be beneficial to show if specific miRNA Argonautes, such as ALG-1, are enriched in yolk granules.

Minor Critiques:
1. Describe clearly the timing of auxin treatments in all experiments. Is RME-2 depletion initiated at L3, as the case of Drosha and Parsha depletion? Also, describe whether there is a noticeable difference in brood size following Drosha and Parsha depletion under unstressed conditions.

2. What is DiD staining? The role of DiD staining in the purification of yolk granules should be better described.

3. The authors may want to use a consistent approach to display statistical significance. Currently, some figures use FDR (Figure 1E, 3C), while others use P values (Figure 2C) to consider statistical significance.

4. In Figure 3C, the data show that most (39/48) miRNAs enriched in old embryos are yolk-enriched miRNAs. As these data did not show the levels of all yolk-enriched miRNAs, it is difficult to evaluate if all or most of the yolk-enriched miRNAs are elevated, as described at line 177. Perhaps consider updating the description at line 177.

5. In my opinion, the authors have not yet provided convincing data to conclude that yolk-enriched miRNAs act in the intestine. Therefore, the description "Together, these data suggest that yolk-enriched miRNAs accumulate in the intestine of larvae along with yolk, where they regulate transcript levels." at line 170 is better suited for the Discussion section.

6. In Figure 6, what is the definition of worms passing L1?

Reviewers #3-4 (Remarks to the Author):

In this work, the authors explore the transfer of miRNA from mother C. elegans to its progeny via the yolk. In oviparous animals, the mother deposits a lipoprotein-rich yolk in the egg. This is made by somatic tissues but is passed on to the next generation, providing a possible means for transferring “information”, in addition to the nutritional requirement, to the next generation. Here, the authors present work that supports the idea that miRNAs are transferred from somatic tissue to the next generation together with the yolk. In addition, the authors present some data that may be consistent with the transferred miRNAs playing a role in the physiology of the next generation.

The manuscript can be divided in two parts. In the first part, the authors build a case for miRNAs being present in large yolk “particles” that are made in the intestine and taken up by the developing oocytes. This is supported by four lines of evidence. Formally, none of these pieces of evidence by itself is sufficient to exclude the alternative possibility that miRNAs are made in the germline or in the somatic gonad, as each experiment has possible alternative interpretations/caveats as we detail below. However, we do think that all together they provide enough support to strongly suggest the idea that miRNAs are co-transferred with the yolk. We don’t, however, think that the authors demonstrate this unequivocally, and this should be reflected in the text.

Evidence 1: “Purification” of yolk particles and sequencing of associated small RNAs. The authors use a strain with GFP labeled yolk protein and a degron on the yolk receptor (RME-2) to prevent yolk uptake into the gonad, and then use fractionation via centrifugation and FACS to purify yolk particles. They then sequence small RNAs associated with this fraction as well as with a cruder, non-sorted fraction and ask which small RNAs are enriched. miRNAs are mostly enriched in the yolk fraction while other small RNAs are less so – although a fraction of piRNAs are also enriched.

This biochemical strategy has two caveats: 1) the use of the RME-2 degron could result in non-physiological interactions between VIT-2 and miRNAs within pseudocoelom, and 2) it is unclear how stable is the yolk aggregate during purification process and whether it’s not capturing random small RNA species from the lysate. The MA plot in Fic 1c shows that most “yolk-enriched miRNAs” are also the most abundant ones, could these miRNAs be pulled down after lysis through unspecific interaction? This could also explain why a fraction of piRNAs, which are produced in the germline, is enriched – not all piRNAs are equally abundant and it could be that only the abundant ones are sufficiently captured.

Evidence 2: Depletion of the yolk receptor RME-2 and sequencing of maternally deposited miRNAs in 2-cell embryos. This shows that many of the miRNAs enriched in yolk are depleted in embryos. However, practically all miRNAs are depleted in this scenario (as opposed to the experiment described below, compare Fig 2e to 3c), raising the concern that RME-2 depletion causes a generally “sick germline”. This is experiment is consistent with the RME-2 dependent uptake of miRNAs in the germline, but it is also consistent with a scenario in which a germline without RME-2 does not produce miRNAs.

Evidence 3: Depletion of miRNA biogenesis factors in the maternal intestine and sequencing of maternally deposited miRNAs in 2-cell embryos. This is a very nice experiment in which the authors use a tissue-specific degron approach to deplete drosha and pasha in the intestine of the mothers and then assess the miRNA content of the embryos. They see a specific subset of miRNAs going down in 2-cell embryos (not a complete depletion like they saw in the RME-2 degron). The strength of the argument that these are miRNAs that travel from the intestine to the oocytes relies heavily on the degron being extremely specific to intestine. Could the authors provide better images for Extended Fig 2a. to show the specificity of the outcome of the auxin treatment, and not only for germline but also for the somatic gonad? In addition, despite this being the most convincing experiment, the DP-degron experiment also has the possible alternative explanation that any changes could be due to a generally “sicker intestine” unable to support proper germline function, and not exclusively due to loss of miRNA transfer from the intestine to the germline. We are aware that the authors control for VIT-2::GFP transfer to embryo, yet there are likely other effects from having an intestine that is missing a whole class of post-transcriptional regulators. A brood size count would be a good additional control to have.

Evidence 4: Correlation of amount of maternally deposited miRNAs with the amount of yolk, which is known to vary with maternal age. The authors sequence miRNAs in 2-cell embryos derived from 1-day old or 3-day old mothers and find more miRNAs in the 3-day old-derived embryos, correlating with the known increased yolk deposition. This does not exclude a yolk-unrelated effect of age on production of germline miRNAs.

We think that further support for the idea that miRNAs are transferred with yolk and its possible consequences will eventually have to be grounded on a molecular mechanism that explains how miRNAs end up in yolk particles in the intestine, and only then there may be a more formal exclusion of the alternative hypothesis. While we realize that figuring out such a mechanism could take years and is not necessary for this paper, it would be important to at least provide some speculation for the yolk-miRNA co-transfer mechanism. How is the particle pre-assembled in intestine? How do the authors hypothesize the miRNA get loaded with the yolk protein from the secretory pathway? Are the miRNAs loaded into Argonaute?

The individual lines of argumentation could be strengthened in the following ways:
1. The author's model implies only miRNAs expressed in intestine can be transferred to the oocytes/embryos. The authors could compare the list of "yolk-enriched" miRNA to previously characterized miRNA expression patterns to strengthen their argument.
2. A post-lysis mixing experiment would show how much association of miRNAs with yolk can happen during purification. The authors could take RNA from a different species and add it to their lysate and then subject it to purification and see how much exogenous miRNA can make it into these yolk particles.
3. Brood size analysis of the intestinal DP degron strain and better analysis that degradation only happens in the intestine and not in the germline or somatic gonad.
4. Some miRNAs are presumably transcribed in the germline. These should not be affected in any of the manipulations the authors do.

The second half of the paper focuses on the possible function of the transferred miRNAs from the intestine to the germline. To test this, the authors first look at changes in gene expression in progeny derived from mothers with or without intestinal miRNA biogenesis. There are no differences in the 2-cell embryos, but the authors see some differences in RNAseq at the L1 larval stage. The authors refer to these as "substantial differences" but there are 63 differentially expressed genes (out of thousands). The authors suggest that the upregulated transcripts in animals whose mothers did not produce intestinal miRNAs are increased because of the lack of inheritance of these intestinal, maternal miRNAs. This seems highly unlikely. The miRNAs that the authors propose may regulate these genes are among the most highly transcribed zygotic miRNAs. To make these claims, the authors should sequence miRNAs from L1s derived from the two conditions and assess whether the maternal contribution plays any role at that stage.

The experiments testing for stress resilience upon degradation of the maternal miRNA biogenesis in the intestine are interesting, but the authors cannot disentangle the possibility that all these effects are due to mothers with "sick intestines" producing worse quality embryos for reasons other than inherited miRNAs. Truly testing the functionality of the inherited miRNAs would require mutational analysis of individual miRNAs and their targets, in our opinion.

Altogether, while we think the idea of miRNA inheritance via yolk is sufficiently supported, whether this has any functional consequence is not sufficiently tested.

Reviewer #5 (Remarks to the Author):

Aupérin, Singh et al. report that provisioning yolk from the intestine to the germline regulates stress responses in progeny in C. elegans. This study characterized small RNAs in yolk originating from the intestine and identified miRNA as a key epigenetic source that impacts transcriptome, development, and survival of larvae under harsh environments. The authors also showed that maternal age and environmental stress affect the yolk-mediated inheritance of miRNA, thereby securing progeny protection.
Overall, the manuscript is clear to follow with the help of the diagrams. Adequate experimental approaches convincingly address yolk-enriched miRNA as a novel heritable epigenetic element. Although yolk inheritance was already known to have beneficial effects on offspring phenotypes, this study extends the field of small RNA-mediated regulation of soma to germline communications and inheritance.
Major concerns:
1. Links between yolk-enriched miRNA and stress resistance in progeny are vague. The role of lin-4 and miR-235 in oxidative stress responses has been tested only in adult animals in previous studies (ref. 37 and 38 of this paper). It remains determined whether lin-4 and miR-235 in yolk are responsible for oxidative stress resistance in progeny during development. This type of functional validation would further strengthen the major claim of the paper.
2. The authors did not fully rule out the potential effects of auxin/5-Ph-IAA treatment -independently of degron-mediated degradation of target proteins- on observations made with the degron strains, such as VIT-2::GFP accumulation in oocytes (Fig. 1d) as well as stress resilience phenotypes of progeny (Fig. 4 and Fig. 6). Inclusion of control strains preferably such that express degron-tagged RME-2 or DP but do not carry the TIR1 transgene would clarify this concern.
3. The phenotypic variability without exposing to stressors that can also be affected by loss of yolk-enriched miRNA needs to be considered to assess stress resilience in Fig. 4 and Fig. 6. Additional controls without stress conditions should be included to address this concern.
Additional concerns:
1. In Extended Data Fig. 1b, images of VIT-2::GFP with auxin treatment and VIT-2::GFP; RME-2 degron without auxin treatment should also be included.
2. "Increasing paraquat concentrations were associated with compromised development and decreased larval lengths, as expected (Fig. 4a)"(lines 196-198 in the text). However, compromised development was not proved in Fig. 4a. For instance, a similar experiment but with paraquat as conducted in Fig 6b, d would help address this.
3. The authors should complement the comparison of yolk-enriched miRNA from old mothers and NaCl-treated mothers by

adding that from mothers exposed to starvation (Extended Data Fig. 5c): are there any yolk-enriched miRNA inherited irrespective of maternal conditions tested?
4. For clarity of the control condition for osmotic stress, it would be better to explain that standard NGM plates contain 50 mM NaCl in legends and methods.
5. Providing genetic information on tissue-specific degron strains in the text or legend would help readers understand experiments without access to supplementary tables of a complete strain list.

** As a service to authors, Springer Nature Limited provides authors with the ability to transfer a manuscript that one journal cannot offer to publish to another journal, without the author having to upload the manuscript data again. To transfer your manuscript to another NPG journal using this service, please click on Link Redacted

** For Springer Nature Limited general information and news for authors, see http://npg.nature.com/authors.

Version 1:

Decision Letter:

3rd Jul 2025

Dear Dr. Cecere,

Thank you for your letter concerning your manuscript "Soma-to-Germline miRNA inheritance via Yolk Promotes Stress Resilience in Progeny". I sincerely apologize for the delay in processing your appeal.

We have now had a chance to discuss the points you raised in detail, and we have decided to send your revised paper back out to review. Before we can proceed with peer review, please provide the additional files below, which are needed for re-review, alongside your revised manuscript and full point-by-point (verbatim) responding to all the previous reviews:

-- We want to ensure that the methods and statistics reporting in our papers are of the highest quality. To that end, we ask authors to fill out a Reporting Summary that collects information on experimental design and reagents. This document can be found by following the link below:

Reporting Summary:
https://www.nature.com/documents/nr-reporting-summary.pdf

Please note that the form is dynamic ‘smart pdfs’ and must, therefore, be downloaded and completed in Adobe Reader. We will then flatten the form for ease of use by the reviewers. If you would like to reference the guidance text as you complete the template, please access these flattened versions at http://www.nature.com/authors/policies/availability.html. Note that you are not required to revise your paper to include the information provided in the reporting summary.

-- Please provide a Supplementary Figure including unprocessed images of all gels/blots in the form of a multi-page pdf file. Please ensure that blots/gels are labeled and the sections presented in the figures are clearly indicated.

-- Please provide a Supplementary Table including all numerical source data in Excel format, with data for different figures provided as different sheets within a single Excel file. The file should include source data giving rise to graphical representations and statistical descriptions in the paper and for all instances where the figures present representative experiments of multiple independent repeats, the source data of all repeats should be provided.

You can use the link below to be taken directly to the site and submit your manuscript:

Link Redacted

Thank you again for your patience and we look forward to your resubmission,

Sincerely,

Melina Casadio, PhD
Locum Chief Editor, Nature Structural & Molecular Biology
ORCID ID: https://orcid.org/0000-0003-2389-2243

Version 2:

Decision Letter:

Nature Structural & Molecular Biology NSMB-A49499B

1st Aug 2025

Dear Dr. Cecere,

Thank you for submitting your revised manuscript, "Soma-to-Germline miRNA inheritance via Yolk Promotes Stress Resilience in Progeny" and thank you very much for your patience with the process. The original reviewers provided comments (below) and as before Revs#3-4 co-reviewed. You will see that, while some referees offered positive comments, some persisting concerns about the strength of the conclusions (including the functional studies), in particular from Revs#3-4, remain. Regrettably, based on these comments, we cannot offer to publish the study in Nature Structural & Molecular Biology.

I am very sorry we could not be more positive on this occasion. We are happy to consult with our colleagues to assess the suitability for a transfer to a sister journal, if you'd like. We hope the referees' comments will be useful to you in revising the manuscript for submission elsewhere.

Sincerely,

Melina Casadio, PhD
Locum Chief Editor, Nature Structural & Molecular Biology
ORCID ID: https://orcid.org/0000-0003-2389-2243

Reviewer #1 (Remarks to the Author):

I thank the authors for addressing my concerns and for performing experiments on the regulation of hrg-1 by miR-85. The proteomic analysis presented also add to the evidence suggesting that miRNAs transported along with yolk could function in embryos. Together, these findings strengthen the paper and I congratulate the authors on this important contribution to our understanding.

Reviewer #2 (Remarks to the Author):

The authors have properly addressed my concerns in their revised manuscript. Importantly, the new results further strengthened their conclusion on the direct role of yolk-inherited miRNAs in the stress resilience of C. elegans progeny. Aside from a few very minor questions and suggestions that I believe the authors can clarify in the manuscript; I continue to view this as a breakthrough study that will open a new field for investigating the roles of yolk-carried small RNAs in intergenerational gene regulation. I fully recommend this manuscript for its publication.

Sincerely,
Heng-Chi Lee

Minor comments/suggestions:
1. While the authors show that purification of yolk granules appears to enrich for VIT-GFP in wild-type animals, the extent of this enrichment remains unclear. Additionally, the figures do not show any granularity of VIT-1::GFP. Could the authors clarify whether yolk granules were observed in the body cavity, embryos, or other tissues?

2. Given that many piRNAs and 22G-RNAs are germline-enriched, could the authors speculate why piRNAs and 22G-RNAs are not depleted in the yolk-enriched small RNA pool (Figure 1B)?

3. The nomenclature "DE miRNA" and "DP degron" may be difficult to interpret and potentially confusing to readers. Would the authors consider using more descriptive terms such as "RME-2–dependent miRNAs" or "intestinal miRNA depletion"?

4. In Figure 4A, under non-paraquat conditions, do larvae from day 3 mothers grow faster than those from day 1 mothers at 72 hours? Currently, Extended Data Figure 6E shows results at 44 hours, but not at 72 hours as in the main figure. It would be helpful to include this comparison.

5. The nature of the mir-85 binding site mutation generated in the study should be clearly described in Extended Data Figure 4—whether it involves a specific nucleotide change in the seed sequence or a deletion of the entire site. Additionally, the predicted base-pairing between mir-85 and hrg-1 should be shown.

Reviewer #3 (Remarks to the Author):

In this version of the manuscript, Auperin et al. address multiple concerns from the previous round of review. We commend the authors' efforts and the carefully controlled experiments, such as showcasing the specificity of the tissue-specific degron system, the technical details of the sorting strategy, and highlighting the relatively invariant level of germline miRNAs. Taken together, the evidence convincingly demonstrates yolk-mediated miRNA transfer to the embryo. However, the latter half of the manuscript, which focuses on the potential functional implications of this observed phenomenon, remains incomplete. To maintain the key claim in the title and the overall work, the authors actually need to demonstrate the functionality of these yolk-associated miRNAs.
The claim of functionality is first based on the experiments with the DP-degron and mothers of different ages, using paraquat resistance as a readout (Figure 4). In that experiment, it is clear that maternal age, which correlates with yolk deposition, correlates with paraquat resistance. However, in that context, removing intestinal miRNAs has a small contribution. The conclusion from this experiment should be that other components of yolk, or other aspects of maternal age contribute to that resistance.
In the second set of experiments, the authors use the degron again but now in an osmotic stress paradigm (Figure 7). In that context, it looks like miRNAs play a more prominent role. This experiment is done with mothers of unspecified age. It would be good to know the precise ages and preferably compare Day 1 and Day 3. But most importantly, to interpret this and the previous experiment, we are missing the direct testing of yolk accumulation with maternal age in the DP genetic background under auxin treatment. A well-executed but incomplete control was performed in Extended Data Fig. 3, demonstrating that DP-degron mothers do not have gross yolk transfer defects; however, this analysis was limited to embryos from mothers of unspecified ages. It would be necessary to confirm that, at Day 3 with auxin treatment, yolk still accumulates more than in Day 1 mothers and is not disrupted by intestinal DP-degron treatment. This would allow the effect to be more confidently attributed to miRNAs rather than to other general components of the yolk.
The next experiment the authors use to support the role of intestinal miRNAs in the next generation is the characterization of the miR-85 mutants. The authors test one of the original deletion alleles, which was generated by heavy mutagenesis, in the paraquat resistance assay. Without rescue and/or generation of an independent CRISPR allele, this experiment should not be interpreted as proof that this miRNA is involved in stress. It is also unclear why the authors then switch again the stress context in which they study the effects of miR-85 on gene expression, but they do not show the effects of miR-85 mutation on their osmotic stress paradigm.
Finally, the authors try to connect miR-85 to one of its deregulated targets, hrg-1. The binding site mutation experiment is an elegant way to evaluate the possible direct effect of miR-85 on hrg-1. However, hrg-1 expression levels in the binding site mutant worms show enormous variation. Without the one higher datapoint, the other two datapoints would indicate that the binding site mutant looks just like wild type. The use of a non-parametric test with these three datapoints is not very meaningful. Moreover, there is no direct functional link provided here, as there is no phenotype evaluation for hrg-1 binding site mutants. For instance, if these mutants had any effect on L1 survival similar to Figure 7 could strengthen this argument. The evidence showing derepression of hrg-1 mRNA alone is insufficient to attribute resilience as being mediated by miR-85. A discussion in the text providing a model or speculation about the role of hrg-1 in stress responses should also be included.

Minor points:
Figure 4: in this experiment, the goal was to test whether embryos from mothers of different ages exhibit differential resilience to paraquat-induced stress, using L1 length as a readout of worm growth. However, there is no control condition without paraquat included. Extended Data Fig. 6e provides an incomplete control condition, but the measurement was performed at a different time point (44 hours instead of the 72 hours used in other assays) and includes only progeny from Day 1 mothers. As a result, it is challenging to establish a direct link using this readout. A similar control group setup, such as the one used in Extended Data Fig. 6a, would be helpful to demonstrate whether the effect is paraquat-dependent.

Page 28 Extended Data Fig. 1e:
The legend describes this as a western blot, but no antibody was specified for the blot. Based on the description in the method section (Page 46, Line 916 to Page 47, Line 920), it appears to be an anti-GFP blot. Labeling the band and specifying the antibody would better orient readers.

Minor Formatting:
1. Page 3 Line 80:
C. elegans should be italicized.

Reviewer #4 (Remarks to the Author):

In this version of the revision, Auperin et al. address multiple concerns from the previous round of review. We commend the authors' efforts and the carefully controlled experiments, such as showcasing the specificity of the tissue-specific degron system, the technical details of the sorting strategy, and highlighting the relatively invariant level of germline miRNAs. Taken together, the evidence convincingly demonstrates yolk-mediated miRNA transfer to the embryo. However, the latter half of the manuscript, which focuses on the potential functional implications of this observed phenomenon, remains incomplete. To maintain the key claim in the title and the overall work, the authors actually need to demonstrate the functionality of these yolk-associated miRNAs.
The claim of functionality is first based on the experiments with the DP-degron and mothers of different ages, using paraquat resistance as a readout (Figure 4). In that experiment, it is clear that maternal age, which correlates with yolk deposition, correlates with paraquat resistance. However, in that context, removing intestinal miRNAs has a small contribution. The

conclusion from this experiment should be that other components of yolk, or other aspects of maternal age contribute to that resistance.
In the second set of experiments, the authors use the degron again but now in an osmotic stress paradigm (Figure 7). In that context, it looks like miRNAs play a more prominent role. This experiment is done with mothers of unspecified age. It would be good to know the precise ages and preferably compare Day 1 and Day 3. But most importantly, to interpret this and the previous experiment, we are missing the direct testing of yolk accumulation with maternal age in the DP genetic background under auxin treatment. A well-executed but incomplete control was performed in Extended Data Fig. 3, demonstrating that DP-degron mothers do not have gross yolk transfer defects; however, this analysis was limited to embryos from mothers of unspecified ages. It would be necessary to confirm that, at Day 3 with auxin treatment, yolk still accumulates more than in Day 1 mothers and is not disrupted by intestinal DP-degron treatment. This would allow the effect to be more confidently attributed to miRNAs rather than to other general components of the yolk.
The next experiment the authors use to support the role of intestinal miRNAs in the next generation is the characterization of the miR-85 mutants. The authors test one of the original deletion alleles, which was generated by heavy mutagenesis, in the paraquat resistance assay. Without rescue and/or generation of an independent CRISPR allele, this experiment should not be interpreted as proof that this miRNA is involved in stress. It is also unclear why the authors then switch again the stress context in which they study the effects of miR-85 on gene expression, but they do not show the effects of miR-85 mutation on their osmotic stress paradigm.
Finally, the authors try to connect miR-85 to one of its deregulated targets, hrg-1. The binding site mutation experiment is an elegant way to evaluate the possible direct effect of miR-85 on hrg-1. However, hrg-1 expression levels in the binding site mutant worms show enormous variation. Without the one higher datapoint, the other two datapoints would indicate that the binding site mutant looks just like wild type. The use of a non-parametric test with these three datapoints is not very meaningful. Moreover, there is no direct functional link provided here, as there is no phenotype evaluation for hrg-1 binding site mutants. For instance, if these mutants had any effect on L1 survival similar to Figure 7 could strengthen this argument. The evidence showing derepression of hrg-1 mRNA alone is insufficient to attribute resilience as being mediated by miR-85. A discussion in the text providing a model or speculation about the role of hrg-1 in stress responses should also be included.

Minor points:
Figure 4: in this experiment, the goal was to test whether embryos from mothers of different ages exhibit differential resilience to paraquat-induced stress, using L1 length as a readout of worm growth. However, there is no control condition without paraquat included. Extended Data Fig. 6e provides an incomplete control condition, but the measurement was performed at a different time point (44 hours instead of the 72 hours used in other assays) and includes only progeny from Day 1 mothers. As a result, it is challenging to establish a direct link using this readout. A similar control group setup, such as the one used in Extended Data Fig. 6a, would be helpful to demonstrate whether the effect is paraquat-dependent.

Page 28 Extended Data Fig. 1e:
The legend describes this as a western blot, but no antibody was specified for the blot. Based on the description in the method section (Page 46, Line 916 to Page 47, Line 920), it appears to be an anti-GFP blot. Labeling the band and specifying the antibody would better orient readers.

Minor Formatting:
1. Page 3 Line 80:
C. elegans should be italicized.

Reviewer #5 (Remarks to the Author):

In the revised version the authors have adequately addressed and clarified all my comments on the original manuscript. The inclusion of functional validation data and additional controls make this now a very convincing paper that clearly advances the field.

Minor suggestion:
The authors may want to add the age of TIR1-negative DP degron mothers in the legend of Extended Data Fig6(d) in the revised manuscript.

Björn Schumacher

** As a service to authors, Springer Nature Limited provides authors with the ability to transfer a manuscript that one journal cannot offer to publish to another journal, without the author having to upload the manuscript data again. To transfer your manuscript to another NPG journal using this service, please click on Link Redacted

** For Springer Nature Limited general information and news for authors, see http://npg.nature.com/authors.

Version 3:

Decision Letter:

Our ref: NSMB-A49499C-Z

5th Mar 2026

Dear Dr. Cecere,

Thank you for submitting your revised manuscript "Soma-to-Germline miRNA inheritance via Yolk Promotes Stress Resilience in Progeny" (NSMB-A49499C-Z). It has now been seen by the original referees and their comments are below. The reviewers find that the paper has improved in revision, and therefore we'll be happy in principle to publish it in Nature Structural & Molecular Biology, pending minor revisions to satisfy the referees' final requests and to comply with our editorial and formatting guidelines.

We are now performing detailed checks on your paper and will send you a checklist detailing our editorial and formatting requirements in about a week. Please do not upload the final materials and make any revisions until you receive this additional information from us.

To facilitate our work at this stage, it is important that we have a copy of the main text as a word file. If you could please send along a word version of this file as soon as possible, we would greatly appreciate it; please make sure to copy the NSMB account (cc'ed above).

Thank you again for your interest in Nature Structural & Molecular Biology Please do not hesitate to contact me if you have any questions.

Sincerely,

Carolina

Carolina Perdigoto, PhD
Chief Editor
Nature Structural & Molecular Biology
orcid.org/0000-0002-5783-7106

Reviewer #2 (Remarks to the Author):

In this revised manuscript, the authors have properly and thoughtfully addressed all of my previous comments. In my opinion, they have also properly addressed those insightful critiques raised by Reviewers 3 and 4. The revisions, including additional experiments and clarification of interpretations, have significantly strengthened the manuscript.

In particular, the authors have convincingly addressed concerns regarding the functional contribution of yolk-inherited miRNAs to stress resilience, the specificity of the miR-85 mutant phenotypes, and the robustness of hrg-1 regulation. The generation of an independent CRISPR–Cas9 miR-85 allele, along with RNA-seq analyses across multiple biological replicates, provides strong support that the observed gene expression changes are specifically attributable to miR-85 loss. Moreover, the additional functional assays under osmotic stress clarify that miR-85 contributes partially to inherited resilience, consistent with a model in which multiple yolk-associated miRNAs act together.

Overall, I continue to view this as a groundbreaking study. The authors provide compelling evidence that miRNAs are transmitted intergenerationally through yolk and that these inherited miRNAs have functional consequences for gene expression in progeny, which promote animal fitness and stress resilience. The use of miR-85 knockout and heterozygous mutants, combined with analysis of their progeny, offers particularly convincing support that inherited miRNAs exert biologically meaningful regulatory effects.

While I am somewhat surprised that regulation of hrg-1 by miR-85 appears to be mediated through a single non-canonical (seed pairing with mismatch/bulge) interaction, this represents an interesting mechanistic detail that can be further explored in future studies.

Taken together, the authors have assembled an extensive and impressive body of high-quality data using integrative approaches that strongly support the groundbreaking discovery of the manuscript. I fully support its publication.

Sincerely,
Heng-Chi Lee, Ph.D.

Reviewer #3 (Remarks to the Author):

In this version of manuscript, Auperin et al. include extra controls and clarify important points in their model for yolk miRNA transmission and its functional consequences. We commend the authors’s effort to address our previous concerns.

A minor point:
Pg 38. Ext fig 6e
Please check the label for the auxin groups, should it be -. -. +, +?

Reviewer #4 (Remarks to the Author):

I co-reviewed this manuscript with one of the reviewers who provided the listed reports. This is part of the Nature Structural & Molecular Biology initiative to facilitate training in peer review and to provide appropriate recognition for Early Career Researchers who co-review manuscripts.

Version 4:

Decision Letter:

29th Apr 2026

Dear Dr. Cecere,

We are now happy to accept your revised paper "Soma-to-Germline miRNA inheritance via Yolk Promotes Stress Resilience in Progeny" for publication as a Article in Nature Structural & Molecular Biology.

Acceptance is conditional on the manuscript's not being published elsewhere and on there being no announcement of this work to the newspapers, magazines, radio or television until the publication date in Nature Structural & Molecular Biology.

Over the next few weeks, your paper will be copyedited to ensure that it conforms to Nature Structural & Molecular Biology style. Once your paper is typeset, you will receive an email with a link to choose the appropriate publishing options for your paper and our Author Services team will be in touch regarding any additional information that may be required.

After the grant of rights is completed, you will receive a link to your electronic proof via email with a request to make any corrections within 48 hours. If, when you receive your proof, you cannot meet this deadline, please inform us at rjsproduction@springernature.com immediately.

You will not receive your proofs until the publishing agreement has been received through our system.

Due to the importance of these deadlines, we ask that you please let us know now whether you will be difficult to contact over the next month. If this is the case, we ask you provide us with the contact information (email, phone and fax) of someone who will be able to check the proofs on your behalf, and who will be available to address any last-minute problems.

To assist our authors in disseminating their research to the broader community, our SharedIt initiative provides all co-authors with the ability to generate a unique shareable link that will allow anyone (with or without a subscription) to read the published article. Recipients of the link with a subscription will also be able to download and print the PDF.

As soon as your article is published, you can generate your shareable link by entering the DOI of your article here: http://authors.springernature.com/share. Corresponding authors will also receive an automated email with the shareable link

Note the policy of the journal on data deposition: http://www.nature.com/authors/policies/availability.html.

Your paper will be published online soon after we receive proof corrections and will appear in print in the next available issue. You can find out your date of online publication by contacting the production team shortly after sending your proof corrections.

You may wish to make your media relations office aware of your accepted publication, in case they consider it appropriate to organize some internal or external publicity. Once your paper has been scheduled you will receive an email confirming the publication details. This is normally 3-4 working days in advance of publication. If you need additional notice of the date and time of publication, please let the production team know when you receive the proof of your article to ensure there is sufficient time to coordinate. Further information on our embargo policies can be found here:
https://www.nature.com/authors/policies/embargo.html

You can now use a single sign-on for all your accounts, view the status of all your manuscript submissions and reviews, access usage statistics for your published articles and download a record of your refereeing activity for the Nature journals.

If you have not already done so, we strongly recommend that you upload the step-by-step protocols used in this manuscript to the Protocol Exchange. Protocol Exchange is an open online resource that allows researchers to share their detailed experimental know-how. All uploaded protocols are made freely available, assigned DOIs for ease of citation and fully searchable through nature.com. Protocols can be linked to any publications in which they are used and will be linked to from your article. You can also establish a dedicated page to collect all your lab Protocols. By uploading your Protocols to Protocol Exchange, you are enabling researchers to more readily reproduce or adapt the methodology you use, as well as increasing the visibility of your protocols and papers. Upload your Protocols at www.nature.com/protocolexchange/. Further information can be found at www.nature.com/protocolexchange/about.

An online order form for reprints of your paper is available at https://www.nature.com/reprints/author-reprints.html. Please let your coauthors and your institutions' public affairs office know that they are also welcome to order reprints by this method.

Authors may need to take specific actions to achieve compliance with funder and institutional open access mandates. If your research is supported by a funder that requires immediate open access (e.g. according to Plan S principles or the NIH public access policy) then you should select the gold OA route, and we will direct you to the compliant route where possible. Because authors warrant under our subscription licensing terms that they haven't committed to licensing any version of their article under a licence inconsistent with the terms of our agreement – including the applicable embargo period – publication under the subscription model isn't suitable for authors whose funders require no embargo.

In approximately 10 business days you will receive an email with a link to choose the appropriate publishing options for your paper and our Author Services team will be in touch regarding any additional information that may be required.

You will not receive your proofs until the publishing agreement has been received through our system.

If you have any questions about our publishing options, costs, Open Access requirements, or our legal forms, please contact ASJournals@springernature.com

Sincerely,

Carolina Perdigoto, PhD
Chief Editor
Nature Structural & Molecular Biology
orcid.org/0000-0002-5783-7106

Click here if you would like to recommend Nature Structural & Molecular Biology to your librarian:
http://www.nature.com/subscriptions/recommend.html#forms

Open Access This Peer Review File is licensed under a Creative Commons Attribution 4.0 International License, which permits use, sharing, adaptation, distribution and reproduction in any medium or format, as long as you give appropriate credit to the original author(s) and the source, provide a link to the Creative Commons license, and indicate if changes were made.
In cases where reviewers are anonymous, credit should be given to 'Anonymous Referee' and the source.
The images or other third party material in this Peer Review File are included in the article’s Creative Commons license, unless indicated otherwise in a credit line to the material. If material is not included in the article’s Creative Commons license and your intended use is not permitted by statutory regulation or exceeds the permitted use, you will need to obtain permission directly from the copyright holder.
To view a copy of this license, visit https://creativecommons.org/licenses/by/4.0/

We would like to sincerely thank all the reviewers for their thoughtful and constructive comments, which were instrumental in improving the quality and clarity of our manuscript. In this document, we provide a detailed, point-by-point response to each reviewer comment.

Changes made to the manuscript in response to these comments are highlighted in purple in the revised text.

Reviewer #1 (Remarks to the Author):

The authors present the isolation of yolk from C. elegans and the analysis of the impact that RNAs in yolk can have on the development of progeny. They identify a set of miRNAs that are claimed to be transported along with yolk from the intestine to progeny. They use Auxin-induced degrons for conditional depletion of the yolk receptor in the germline and the miRNA processing enzymes in the intestine. Sequencing of 2-cell embryos is used to select against miRNAs that could be transcribed early in the zygote. They also present assays for stress tolerance in progeny correlated with parental experience.

A major strength of the paper is the biochemical isolation of yolk and its analysis, which is a technical challenge. However, there are weaknesses that make the central claims of the paper ultimately unsupported. The biggest concern is that there is no direct evidence that the micro RNAs from the parental intestine are imported into the cytosol in progeny and that they function specifically to cause the observed changes in gene expression. Experiments addressing what is required in addition to initial uptake via RME-2, particularly for crossing the endomembranes into the cytosol, will strengthen the claims in the paper. Additionally, making mutations in miRNAs in parents and compensatory mutations in targets that are introduced only in progeny by crossing could provide the compelling evidence needed for the claim of inherited miRNAs functioning in progeny (TargetScan predictions are inadequate for this key claim). Finally, in explaining changes in gene expression, alternative hypotheses are not adequately considered.

In short, there is evidence for presence of parental miRNAs in yolk, but not for their functioning in progeny. Therefore, the title of the paper is unsupported.

We would like to thank Reviewer #1 for the thorough and constructive evaluation of our manuscript. We appreciate the acknowledgment of the technical achievement in biochemically isolating yolk and analyzing its small RNA cargo. We also agree that elucidating the functional impact of yolk-transferred miRNAs in progeny is crucial to strengthen our central claim. We fully acknowledge the importance of providing direct evidence that miRNAs inherited via yolk are not only present in embryos but also functionally regulate gene expression. To address this concern, we performed new experiments focusing on *miR-85*. We previously found that *miR-85* is the most strongly enriched yolk miRNA in 2-cell embryos derived from mothers subjected to osmosis stress compared to control mothers. We now demonstrate that: 1) Mutation of *miR-85* abrogates the downregulation of *hrg-1* mRNA, specifically in embryos derived from stressed mothers (**Fig. 6a, c**). 2) Embryos derived from osmotically stressed mothers with mutations in the *miR-85* binding site of *hrg-1* mRNA fail to downregulate this transcript, phenocopying the loss of *miR-85* function (**Fig. 6b**). 3) Importantly, zygotic *miR-85* expression do not show target repression, indicating that inherited *miR-85*, rather than newly transcribed zygotic *miR-85*, is responsible for *hrg-1* regulation in early development (**Fig. 6a and Extended Data Fig. 9 b-d**). These experiments fulfill the critical requirement for demonstrating that inherited miR-85 is both present and mechanistically active in early embryos, directly repressing a specific mRNA target in a sequence-specific and inheritance-dependent manner.

We also agree with the reviewer that the mechanism by which yolk-inherited miRNAs escape endosomal compartments to enter the cytosol remains an open and important question. We have now expanded our Discussion (**p. 13–14, lines 388–425**) to address this point in depth, drawing on relevant literature about endosomal release and Argonaute loading of single-stranded miRNAs, particularly in the context of extracellular vesicles (EVs) and lipoprotein particles (see reply to major comment #1). Importantly, we now provide results from a proteomic analysis of purified yolk granules prior to their uptake into oocytes. Although we did not detect Argonaute proteins in these preparations—likely due to technical limitations or limited material—we identified several RNA-binding proteins and known miRNA-associated cofactors, including TSN-1, VGLN-1, CAR-1, VIG-1, PAB-1, CEY-2, and the HSP90–HSP70 chaperone complex (**Supplementary Table 1**). These proteins are implicated in miRNA stabilization, RNP assembly, and Argonaute loading, and many were previously identified in immunoprecipitates of the miRNA cofactor AIN-2 (*Dallaire et al., Dev Cell, 2018*). Their presence in yolk granules supports our model in which miRNAs are transported in a RNP-bound state and subsequently incorporated into silencing complexes upon cytosolic release in the embryo.

Finally, we have revised the Discussion to acknowledge better and address alternative explanations for the observed changes in gene expression in progeny. However, the specificity of the *miR-85–hrg-1* regulatory interaction, confirmed through genetic and molecular tools, supports a direct role for inherited *miR-85*.

Major comments:

1. It appears that the miRNAs are detected in their single-stranded form (e.g., abundances in Fig. 1e). If that is the case, how is their transport across membranes after endocytosis envisioned? Minimally, some discussion of this aspect will be useful.

We thank Reviewer #1 for raising this important mechanistic point. We agree that a discussion of how mature single-stranded miRNAs might become functionally active following endocytosis is essential, and we have now included a dedicated part in the revised Discussion (**pages 13–14, lines 388–425**). Below we summarize the key points now addressed in the manuscript.

"Upon endocytic uptake of yolk granules into late-stage oocytes, miRNAs are likely released into the cytosol during endosome maturation, where pH-dependent membrane remodeling facilitates the escape of RNA cargo. Single-stranded small RNAs can be efficiently loaded into Argonaute proteins and mediate target repression. Additionally, miRNA silencing complexes, including Argonautes, are enriched in late endosomes and multivesicular bodies, with miRNA loading shown to be pH-dependent during endosomal maturation.

Recent studies have also shown that Argonaute proteins are generally absent from exosomes, suggesting that miRNAs delivered via yolk are likely imported in a free or RNP-associated form and subsequently reloaded into endogenous Argonautes within the embryonic cytosol. This model aligns with our observation of sequence-specific gene regulation by inherited miRNAs in embryos, underscoring a broader role for endosomal trafficking in miRNA-mediated intergenerational communication.

Based on these findings, we propose a model whereby maternal somatic miRNAs, stabilized within yolk granules, are released into the embryonic cytoplasm, loaded into Argonaute complexes, and integrated into gene regulatory networks. This represents a novel soma-to-germline signaling pathway with important implications for heritable stress adaptation."

2. The changes in gene expression caused by the miRNA depletion is relatively modest <~4-fold in all cases for the 63 genes affected (Fig. 2f). Indirect effects that could result in these changes have not been excluded. For example, when miRNA production is disrupted in the parental intestine, what evidence supports the changes in these genes being the result of the import of miRNA into the progeny and not disruption of anything else that is also transported along with yolk. One hypothesis could be that the maternal deposition of heme is impaired, which could explain why heme responsive genes (hrg-1 and hrg-7 highlighted in Fig. 2f are altered). Note that Drosha and Pasha need heme for proper functon(Partin et al., Nature Communications, 2017), which is further suggestive of heme-related feedback.

We thank Reviewer #1 for this insightful comment. We agree that changes in gene expression resulting from parental miRNA depletion could reflect both direct and indirect effects. To specifically address this concern, we have conducted additional experiments using *miR-85* as a model.

In our revised manuscript, we present new evidence showing that *miR-85* directly regulates the *hrg-1* mRNA in progeny in a sequence-specific and inheritance-dependent manner, arguing against indirect heme-related effects as the primary driver. First, we previously identified *miR-85* as the most enriched yolk-associated miRNA in 2-cell embryos derived from osmotically stressed mothers compared to controls. We now demonstrate that *mir-85(n4117)* mutant embryos from stressed mothers exhibit increased levels of *hrg-1* mRNA, suggesting that maternal *miR-85* contributes to the repression of its target under stress. Moreover, when we mutated the predicted *miR-85* binding site in the 3′UTR of *hrg-1*, the stress-induced repression of *hrg-1* in progeny was compromised, providing functional evidence for a direct, sequence-specific interaction between inherited *miR-85* and its embryonic target. Importantly, we show that zygotic expression of *miR-85* is not responsible for the observed gene regulation, indicating that the miRNA-mediated repression occurs specifically through maternal transmission. These observations have now been added to **pages 10–11, lines 296–338** and **Fig. 6** and **Extended Data Fig. 9**, and have been included in the discussion on **page 13, lines 373–388**.

While we cannot completely rule out that miRNA depletion in the parental intestine may alter other yolk-associated factors, the combination of genetic deletion, target-site mutagenesis, and maternal vs. zygotic dissection provides compelling evidence that the observed regulatory effects are due to the inheritance and function of specific miRNAs, such as *miR-85*, rather than general yolk perturbations or systemic heme-related feedback.

3. The effect of paraquat on development appears modest (Fig. 4a). Given the spread of the data, what would an effect size calculation reveal? Given the possibility of alternative ways that the disruption of miRNA production in the intestine could impact the next generation, even observing a large effect size does not guarantee the effect is because of miRNAs that are transported from parent to progeny. A similar concern remains when considering the responses to osmotic stress. The <2-fold changes with large errors observed to be dependent on RME-2 and

Drosha/Pasha in the parental intestine could be indirect – not dependent on miRNA entry into the cytosol in progeny.

We thank the reviewer for bringing this point to our attention. In response, we have now calculated the effect size for all the paraquat experiments shown in **Fig. 4** and report the corresponding Cohen's d values in the updated figure. These values range from modest to large, indicating that even when fold changes are under two-fold, the effects are statistically and biologically meaningful. Importantly, our experimental design leverages thousands of animals per condition, ensuring high statistical power and reproducibility. This scale enables us to detect subtle yet robust changes in a complex, quantitative trait, such as stress resilience.

We fully agree that effect size alone does not establish causality. To directly address the possibility of indirect effects, we have now added new functional experiments using *miR-85*, the most osmotically stressed enriched yolk-associated miRNA (**Figs. 5, 6** and **Extended Data Fig. 9**). These experiments provide strong mechanistic evidence that inherited *miR-85* is both necessary and sufficient to repress the stress-responsive gene *hrg-1* in progeny in a sequence-specific and inheritance-dependent manner. Specifically, we show that both the loss of maternal *miR-85* and mutation of the *miR-85* binding site in the *hrg-1* 3′UTR prevent stress-induced repression of this target. In contrast, zygotic *miR-85* expression does not rescue the effect, demonstrating that gene regulation is mediated by the inherited pool of *miR-85*.

Thus, our revised manuscript combines quantitative effect size assessments with causal and genetic evidence linking yolk-inherited *miR-85* to gene regulation and contributes, along with other yolk-inherited miRNAs, to stress resilience in progeny. This directly addresses the concern that our observations could be indirect consequences of general yolk perturbation or maternal stress and instead support a specific functional role for inherited miRNAs.

Minor comments:

1. While it is noted that there were no 'global enrichments' of piRNAs or 22G RNAs in the sorted yolk fraction, were there gene-specific enrichments or depletions observed? Knowing this would be important for understanding the relative roles of miRNAs and other small RNAs, if any.

We appreciate the reviewer's point and agree that it is important to consider whether other small RNA classes might contribute to yolk-mediated inheritance. While approximately 5% of piRNAs show some enrichment in yolk, none of the 3933 piRNAs detected in 2-cell embryos were differentially expressed upon maternal rme-2 depletion (**Extended Data Fig. 2d**). Therefore, piRNAs are unlikely to play a functional role in this context. By contrast, miRNAs show much stronger enrichment and depletion patterns, justifying our focus on their role in intergenerational gene regulation. The 22G-RNAs were not cloned in our 2-cell embryo sequencing, given the scarcity of material; therefore, their potential heritability in embryos cannot be evaluated.

2. The cutoff for defining yolk-enriched miRNAs seems a little low (log2 fold change of 0.5 instead of the more common 1 or 2, as indeed used for RNA-seq later in the paper).

We thank the reviewer for raising this point. We deliberately chose a $\log_2$ fold change threshold of 0.5 for identifying yolk-enriched miRNAs because our sorting experiments assess enrichment, similar to immunoprecipitation-based approaches (e.g., AGO IP), rather than differential gene expression per se. In such biochemical enrichment studies, a lower threshold is commonly used to capture specific, biologically relevant associations, especially when combined with statistical significance across replicates. Notably, our enrichment analysis was performed across three independent experiments, and depletion experiments further validated candidate miRNAs to strengthen their identification as inherited miRNAs. This approach enables us to avoid overlooking relevant candidates while maintaining rigor.

We hope the additional data and clarifications address Reviewer #1's concerns satisfactorily. We believe that with these revisions, our manuscript will provide a compelling and robust contribution to the field.

Reviewer #2 (Remarks to the Author):

In this study, Auperin et al. investigate the roles of yolk-inherited miRNAs in the stress resilience of progeny in C. elegans. In my opinion, this is a high-impact paper that will open a new field for further investigation into the roles of yolk-carrying small RNA in intergenerational gene regulation. Many of the experiments, including small RNA/mRNA sequencing and the use of an auxin-induced system, are elegantly designed and carefully executed to demonstrate the roles of yolk-carrying miRNA in stress responses of the progeny. Assuming the authors are able to address some of the critiques listed below, I see this manuscript as one of the breakthrough papers in the field of small RNAs.

We sincerely thank Reviewer #2 for the very positive assessment of our work and for the thoughtful and constructive comments. Below, we address each of the reviewer's critiques in detail and outline the revisions we have made to the manuscript.

Main Critiques:
1. The authors use a combination of centrifugation and sorting to isolate GFP::VIT granules, which was considered as yolk granules. Nonetheless, as C. elegans gut granules are known to exhibit autofluorescent signals, such purification will likely contain contamination from gut granules, which should be acknowledged in the text. They also did not show Western blot data or other data, with both positive and negative controls, to demonstrate the effectiveness of GFP sorting (comparing the sorted vs. unsorted signals).

We appreciate the concern regarding potential contamination by autofluorescent gut granules during the isolation of GFP::VIT granules. We specifically designed our sorting strategy to avoid such contamination by including a gating window that excludes gut autofluorescence. As a negative control, we used wild-type N2 worms lacking GFP-tagged VIT-2 to set sorting thresholds. Additionally, we performed DiD staining, which specifically stains the lipid. As the yolk is a lipoprotein complex, we only sorted particles that were positive for both Vit-2::GFP and DiD to increase specificity. We have now included this validation in the revised manuscript (**Extended Data Fig. 1f, g**, and results **p. 4, lines 106–112**), where we detail the exclusion criteria and provide representative flow cytometry plots and controls. These data confirm the specificity of our sorting approach and the effectiveness of the DiD and GFP dual gating strategy.

2. Related to the issue above, there is little description regarding the background of yolk granules. For example, what is known about the stability and function of yolk granules, and how were they purified in previous studies? In addition, if the authors are purifying yolk granules, the description in the abstract should change from "purified the yolk" to "purified the yolk granules."

We agree with the reviewer that additional background on yolk granules is warranted. In the revised manuscript, we have expanded our description of the known properties of yolk granules, including their biosynthesis in the intestine, transport via the pseudocoelom, and stability in embryos (introduction **p. 3, lines 62–84**).
We also clarify that while previous studies have relied on fluorescent reporters or electron microscopy to study yolk biology, to our knowledge, our work represents the first biochemical purification of yolk granules. Accordingly, we have revised the abstract and main text to consistently use the term "purified yolk granules" rather than "purified yolk."

3. miRNA production is tightly regulated to precisely control the expression of its target gene during development, such as in the case of lin-4 miRNA. Therefore, it is important to describe the abundance, not just the enrichment, of yolk-enriched miRNAs in the embryos. For example, it would be valuable to compare the abundance of yolk-enriched miRNAs to other germline-enriched miRNAs (such as the mir-35 families) that are inherited through the cytoplasm of the oocyte/sperm.

We thank the reviewer for highlighting this important point. In addition to presenting the enrichment of specific miRNAs in yolk granules, we now provide quantitative comparisons of their abundance in embryos relative to well-characterized germline-inherited miRNAs, such as the *miR-35* family. These comparisons are illustrated in new MA plots included in the **Extended Data Figures 2c, 3c, 3f, 5b**, which highlight that some yolk-enriched miRNAs are nearly as abundant as the most highly expressed germline miRNAs (e.g., *miR-35* and *miR-51*) in 2-cell embryos. These data strengthen the relevance of yolk-derived miRNAs in early embryogenesis.

4. Are yolk-enriched miRNAs loaded with Argonautes? It would be beneficial to show if specific miRNA Argonautes, such as ALG-1, are enriched in yolk granules.

Given the limited material recovered from yolk granule purification, it was not feasible to perform Western blotting for Argonaute proteins (ALG-1/2). Moreover, mass spectrometry analysis of FACS-purified yolk granules did not detect Argonaute proteins. While this may reflect the sensitivity limitations of MS/MS—especially given the small amount of purified material—the absence of Argonautes aligns with emerging evidence from high-resolution

proteomic studies of extracellular vesicles, where AGO1–4 proteins are generally undetectable (*Jeppesen et al., Cell, 2019*). These findings challenge the long-held notion that miRNA–AGO complexes are co-transported in extracellular vesicles.

In contrast, our proteomic analysis identified several known miRNA silencing complex (miRISC) components that may stabilize and chaperone miRNAs in a pre-loading state, including TSN-1, VGLN-1, CAR-1, VIG-1, PAB-1, CEY-2, and the HSP90–HSP70 chaperone complex. Notably, many of these proteins were previously reported in immunoprecipitates of the miRISC cofactor AIN-2 (*Dallaire et al., Dev. Cell, 2018*), further supporting their functional relevance in miRNA regulation.

Together, these findings suggest that yolk-enriched miRNAs are transported in a single-stranded, RNP-bound form and are subsequently loaded onto Argonaute proteins within the embryonic cytosol. This model is consistent with prior studies showing that Argonaute loading occurs on late endosomes and multivesicular bodies and is regulated by endosomal pH (*Gibbings et al., Nat. Cell Biol., 2009; Ghoshal et al., J. Cell Sci., 2021*). We have incorporated this mechanistic model into the revised manuscript (Results, **p. 4–5, lines 112–117**; Discussion, **p. 13–14, lines 388–425** and **435–442**).

Minor Critiques:

1. Describe clearly the timing of auxin treatments in all experiments. Is RME-2 depletion initiated at L3, as the case of Drosha and Parsha depletion? Also, describe whether there is a noticeable difference in brood size following Drosha and Parsha depletion under unstressed conditions.

We have clarified the timing of all auxin treatments in the revised Methods and Results sections. RME-2 depletion was initiated at the L3 stage, consistent with the timing used for Drosha and Pasha depletion. We also now include brood size data for the Drosha/Pasha degron strain under unstressed conditions (**Extended Data Fig. 3e**), showing no drastic reduction in fertility in the absence of intestinal miRNA production. Additionally, we verify that there is no impact on germline-expressed miRNAs and piRNAs in DP depleted mothers as well as in 2-Cell embryos (**Extended Data Fig. 3c, f-h**).

2. What is DiD staining? The role of DiD staining in the purification of yolk granules should be better described.

We now provide additional details regarding DiD staining in the Methods and Results. DiD (1,1'-Dioctadecyl-3,3,3',3'-tetramethylindocarbocyanine) is a lipophilic dye used to label the lipid component of yolk granules. In our sorting protocol, we selected particles that were positive for both GFP::VIT-2 and DiD to enrich specifically for lipoprotein-rich yolk granules. DiD fluorescence (excitation at 640 nm) enabled us to exclude non-lipid autofluorescent particles and increase the specificity of yolk granule purification.

3. The authors may want to use a consistent approach to display statistical significance. Currently, some figures use FDR (Figure 1E, 3C), while others use P values (Figure 2C) to consider statistical significance.

We appreciate this observation. We have now standardized our statistical annotations throughout the manuscript and figure legends. All comparisons of the differential gene or miRNA expression are reported using false discovery rate (FDR)-adjusted p-values (q-values), as per standard practice for high-throughput data.

4. In Figure 3C, the data show that most (39/48) miRNAs enriched in old embryos are yolk-enriched miRNAs. As these data did not show the levels of all yolk-enriched miRNAs, it is difficult to evaluate if all or most of the yolk-enriched miRNAs are elevated, as described at line 177. Perhaps consider updating the description at line 177.

We have included an MA plot showing the levels of all yolk-enriched miRNAs in **Extended Data Fig. 5b**.

5. In my opinion, the authors have not yet provided convincing data to conclude that yolk-enriched miRNAs act in the intestine. Therefore, the description "Together, these data suggest that yolk-enriched miRNAs accumulate in the intestine of larvae along with yolk, where they regulate transcript levels." at line 170 is better suited for the Discussion section.

We agree with Reviewer #2 that the statement about yolk-enriched miRNAs acting in the intestine is more appropriate for the Discussion section. This has been revised accordingly.

6. In Figure 6, what is the definition of worms passing L1?

We have clarified the meaning of "worms passing L1" in the figure legend. Worms passing L1 are those that successfully develop beyond the L1 larval stage despite exposure to osmotic stress, as defined by *Burton et al., 2017*.

Reviewers #3-4 (Remarks to the Author):

In this work, the authors explore the transfer of miRNA from mother C. elegans to its progeny via the yolk. In oviparous animals, the mother deposits a lipoprotein-rich yolk in the egg. This is made by somatic tissues but is passed on to the next generation, providing a possible means for transferring “information”, in addition to the nutritional requirement, to the next generation. Here, the authors present work that supports the idea that miRNAs are transferred from somatic tissue to the next generation together with the yolk. In addition, the authors present some data that may be consistent with the transferred miRNAs playing a role in the physiology of the next generation.

The manuscript can be divided in two parts. In the first part, the authors build a case for miRNAs being present in large yolk “particles” that are made in the intestine and taken up by the developing oocytes. This is supported by four lines of evidence. Formally, none of these pieces of evidence by itself is sufficient to exclude the alternative possibility that miRNAs are made in the germline or in the somatic gonad, as each experiment has possible alternative interpretations/caveats as we detail below. However, we do think that all together they provide enough support to strongly suggest the idea that miRNAs are co-transferred with the yolk. We don’t, however, think that the authors demonstrate this unequivocally, and this should be reflected in the text.

We thank Reviewers #3–4 for summarizing our work and for carefully evaluating our experiments and potential caveats in the four lines of evidence supporting the idea that miRNAs produced in the intestine are transferred to embryos via yolk. We agree that while each experiment has limitations, together, they build a compelling narrative for the transport of miRNAs with yolk. We have addressed each of the reviewers’ points regarding Evidence 1–4 below.

Evidence 1: “Purification” of yolk particles and sequencing of associated small RNAs. The authors use a strain with GFP labeled yolk protein and a degron on the yolk receptor (RME-2) to prevent yolk uptake into the gonad, and then use fractionation via centrifugation and FACS to purify yolk particles. They then sequence small RNAs associated with this fraction as well as with a cruder, non-sorted fraction and ask which small RNAs are enriched. miRNAs are mostly enriched in the yolk fraction while other small RNAs are less so – although a fraction of piRNAs are also enriched.
This biochemical strategy has two caveats: 1) the use of the RME-2 degron could result in non-physiological interactions between VIT-2 and miRNAs within pseudocoelom, and 2) it is unclear how stable is the yolk aggregate during purification process and whether it’s not capturing random small RNA species from the lysate. The MA plot in Fic 1c shows that most “yolk-enriched miRNAs” are also the most abundant ones, could these miRNAs be pulled down after lysis through unspecific interaction? This could also explain why a fraction of piRNAs, which are produced in the germline, is enriched – not all piRNAs are equally abundant and it could be that only the abundant ones are sufficiently captured.

The reviewers point out two potential caveats:

1. The use of the RME-2 degron may lead to non-physiological interactions between VIT-2 and miRNAs in the pseudocoelom.
2. The stability of yolk aggregates during purification raises the possibility that abundant miRNAs or piRNAs are nonspecifically captured during the process.

Response to Caveat 1:
We performed small RNA sequencing of 2-cell embryos from RME-2-depleted mothers. We found that many of the miRNAs enriched in purified yolk granules are correspondingly depleted in the embryos (**Fig. 1e**), consistent with their physiological transfer via yolk. Notably, piRNAs enriched in yolk granules (~5%) are not depleted in 2-cell embryos (**Extended Data Fig. 2d**), arguing against a general non-specific transfer.

To further minimize potential artifacts from prolonged yolk accumulation, we collected animals within hours of egg laying to reduce pseudocoelomic yolk retention.

Additionally, at the beginning of the project, we also performed yolk purification after exposure to *Pseudomonas* infection and found an increase in yolk-associated miRNAs despite a decrease in total miRNA levels (***Reviewer Fig. 1***). This suggests active and selective miRNA loading into yolk particles. Due to more recent biosafety restrictions, we could not continue working with Pseudomonas and instead used other stressors in our manuscript (osmotic, oxidative, starvation). This is why we have not included these results in the manuscript. Because yolk purification is technically demanding and yields limited material, we present this initial result here in the rebuttal.

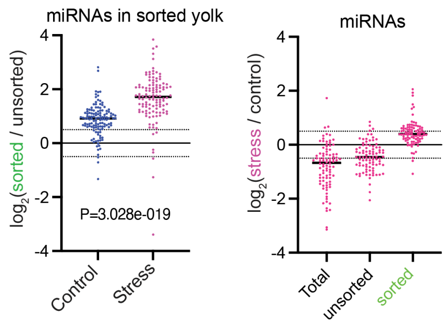

Reviewer Fig. 1: Left, Log$_2$fold change of miRNAs in sorted vs. unsorted yolk fraction from worms grown on Pseudomonas (PA14) or control (OP50) conditions. Right, log$_2$fold change in stress vs. control for total, unsorted, and sorted fractions. n = 3 biological replicates.

Response to Caveat 2:
To address this concern we have sequenced small RNAs from the total whole worm lysate fraction. We generated MA plots comparing miRNA levels in whole-worm lysates to those in our sorted and unsorted yolk fractions. This shows that the yolk-enriched miRNAs vary in abundance and are not only the most highly expressed ones (**Extended Data Fig. 2a**), supporting the idea of selective enrichment.

Evidence 2: Depletion of the yolk receptor RME-2 and sequencing of maternally deposited miRNAs in 2-cell embryos. This shows that many of the miRNAs enriched in yolk are depleted in embryos. However, practically all miRNAs are depleted in this scenario (as opposed to the experiment described below, compare Fig 2e to 3c), raising the concern that RME-2 depletion causes a generally “sick germline”. This is experiment is consistent with the RME-2 dependent uptake of miRNAs in the germline, but it is also consistent with a scenario in which a germline without RME-2 does not produce miRNAs.

We understand the reviewers’ concern that all miRNAs are depleted in 2-cell embryos from mothers depleted of RME-2, raising the possibility that this may reflect a general defect in germline function rather than a specific loss of miRNA uptake. To address this concern, we now provide MA plot analysis showing that germline-specific miRNAs (*miR-35* family) and piRNAs are unaffected in these embryos (**Extended Data Fig. 2c, d**). This indicates that the depletion of other miRNAs is not due to a “sick germline,” but instead reflects a selective failure of miRNA transfer via yolk.

Evidence 3: Depletion of miRNA biogenesis factors in the maternal intestine and sequencing of maternally deposited miRNAs in 2-cell embryos. This is a very nice experiment in which the authors use a tissue-specific degron approach to deplete drosha and pasha in the intestine of the mothers and then assess the miRNA content of the embryos. They see a specific subset of miRNAs going down in 2-cell embryos (not a complete depletion like they saw in the RME-2 degron). The strength of the argument that these are miRNAs that travel from the intestine to the oocytes relies heavily on the degron being extremely specific to intestine. Could the authors provide better images for Extended Fig 2a. to show the specificity of the outcome of the auxin treatment, and not only for germline but also for the somatic gonad? In addition, despite this being the most convincing experiment, the DP-degron experiment also has the possible alternative explanation that any changes could be due to a generally “sicker intestine” unable to support proper germline function, and not exclusively due to loss of miRNA transfer from the intestine to the germline. We are aware that the authors control for VIT-2::GFP transfer to embryo, yet there are likely other effects from having an intestine that is missing a whole class of post-transcriptional regulators. A brood size count would be a good additional control to have.

We thank the reviewers for recognizing the strength of this tissue-specific approach and for raising concerns about potential off-target effects and physiological consequences.
We now provide:
1) New confocal images showing the specificity of the DP degron upon auxin treatment (**Extended Data Fig. 3a**), confirming degradation occurs only in the intestine.
2) Brood size analysis showing no severe reduction in fertility upon DP depletion (**Extended Data Fig. 3e**), arguing against generalized intestinal dysfunction affecting reproduction.
3) Adult small RNA sequencing after DP depletion reveals depletion of intestine-specific miRNAs (as defined by *Brosnan et al., 2021*), while germline miRNAs and piRNAs remain unaffected (**Extended Data Fig. 3f, g**). This supports the specificity of the manipulation and the conclusion that depletion of miRNAs in 2-cell embryos arises from a loss of intestinal expression, not germline defects.

Evidence 4: Correlation of amount of maternally deposited miRNAs with the amount of yolk, which is known to vary with maternal age. The authors sequence miRNAs in 2-cell embryos derived from 1-day old or 3-day old mothers

and find more miRNAs in the 3-day old-derived embryos, correlating with the known increased yolk deposition. This does not exclude a yolk-unrelated effect of age on production of germline miRNAs.

The reviewers raise a valid concern that increased miRNA levels in embryos from older mothers could reflect age-related changes in germline transcription. We now show that most germline-expressed miRNAs (*mir-35* family) do not increase their expression with maternal age (**Extended Data Fig. 5b**), ruling out a global increase in germline miRNA production. We have also shown that environmental stresses, such as osmotic stress and starvation, similarly impact the inheritance of yolk-enriched miRNAs (**Fig. 5c, Extended Data Fig. 7d**), thereby reinforcing that this is not solely an age-related effect. Finally, we have also observed increased inheritance of intestinal miRNAs, such as *miR-77*, under both stress and aging conditions, suggesting that these effects are linked to yolk loading rather than germline miRNA transcription.

We think that further support for the idea that miRNAs are transferred with yolk and its possible consequences will eventually have to be grounded on a molecular mechanism that explains how miRNAs end up in yolk particles in the intestine, and only then there may be a more formal exclusion of the alternative hypothesis. While we realize that figuring out such a mechanism could take years and is not necessary for this paper, it would be important to at least provide some speculation for the yolk-miRNA co-transfer mechanism. How is the particle pre-assembled in intestine? How do the authors hypothesize the miRNA get loaded with the yolk protein from the secretory pathway? Are the miRNAs loaded into Argonaute?

We agree with the reviewer that uncovering the molecular mechanism by which miRNAs are packaged into yolk granules and later incorporated into silencing complexes is a central open question. While this process remains to be fully elucidated and may require substantial future work, we now provide new proteomic evidence and a conceptual model that lay a useful foundation for future investigation.

In the revised Discussion (**p. 13–14, lines 388–425**), we speculate on the possible pathways of miRNA–yolk association, drawing on parallels with extracellular vesicle biology and lipoprotein-based RNA trafficking. We propose that, in the maternal intestine, specific mature miRNAs—potentially in single-stranded form—are selectively sorted into yolk granules during their assembly or budding from the ER-Golgi secretory pathway. These miRNAs may be stabilized and transported in complex with specific RNA-binding proteins and molecular chaperones.

To support this model, we now include results from our mass spectrometry analysis of sorted yolk granules, which revealed the presence of several RNA-binding proteins and known miRNA co-factors, including TSN-1, VGLN-1, CAR-1, VIG-1, PAB-1, CEY-2, and the HSP90–HSP70 chaperone complex (**Supplementary Table 1**). These factors are implicated in miRNA processing, RNP assembly, and Argonaute loading. Their presence in yolk granules suggests that miRNAs are not passively incorporated, but rather co-transported as part of specialized ribonucleoprotein complexes assembled in the maternal intestine. A similar repertoire of RNA-binding proteins has also been reported in the proteome of secreted yolk (*Kern et al., Nat. Commun., 2021*) (**Supplementary Table 1**), indicating that these factors are consistently associated with yolk throughout its lifecycle.

Moreover, a prior proteomic analysis of AIN-2, a key Argonaute cofactor, identified VIT-6 together with TSN-1, CAR-1, and PAB-1 as interaction partners (*Dallaire et al., Dev. Cell, 2018*). The overlap between our yolk proteome and the AIN-2 interactome suggests potential continuity between the miRISC-associated environment in the soma and the yolk cargo, raising the possibility that partial silencing complexes may be pre-assembled in the intestine prior to yolk secretion and germline delivery.

Although Argonaute proteins (e.g., ALG-1/2) were not detected in our yolk granule proteome—likely due to technical limitations or the small amount of starting material—we cannot exclude their presence. Once yolk granules are internalized into oocytes, the acidic environment of maturing endosomes may promote cytosolic release of miRNA–RNP cargo and enable Argonaute loading, consistent with models of pH-dependent endosome-mediated RNA delivery (*Gibbings et al., Nat. Cell Biol., 2009; Ghoshal et al., J. Cell Sci., 2021*).

Together, these findings support a working model in which maternal intestinal miRNAs are stabilized and packaged into yolk granules as RNP complexes, trafficked into the germline, and subsequently incorporated into silencing machinery in the embryo. While speculative, this model is grounded in emerging biochemical and cell biological data and is now discussed more fully in the revised manuscript.

The individual lines of argumentation could be strengthened in the following ways:

1. The author's model implies only miRNAs expressed in intestine can be transferred to the oocytes/embryos. The authors could compare the list of "yolk-enriched" miRNA to previously characterized miRNA expression patterns to strengthen their argument.

We now include such comparisons in our plots, annotating intestine- and germline-expressed miRNAs (**Extended Data Fig. 2, 3, 5**).

2. A post-lysis mixing experiment would show how much association of miRNAs with yolk can happen during purification. The authors could take RNA from a different species and add it to their lysate and then subject it to purification and see how much exogenous miRNA can make it into these yolk particles.

Although we have not conducted a cross-species mixing experiment, our stress experiments (Pseudomonas infection) support the incorporation of specific miRNA into the yolk. These results are presented in ***Reviewer Fig. 1***.

3. Brood size analysis of the intestinal DP degron strain and better analysis that degradation only happens in the intestine and not in the germline or somatic gonad.

Now included (**Extended Data Fig. 3a, e**), showing no severe fertility defects and degradation confined to the intestine.

4. Some miRNAs are presumably transcribed in the germline. These should not be affected in any of the manipulations the authors do.

We confirm that germline miRNAs and piRNAs remain unaffected in DP- and RME-2–depleted samples, supporting the specificity of miRNA transfer via the yolk (**Extended Data Figs. 2 and 3**).

The second half of the paper focuses on the possible function of the transferred miRNAs from the intestine to the germline. To test this, the authors first look at changes in gene expression in progeny derived from mothers with or without intestinal miRNA biogenesis. There are no differences in the 2-cell embryos, but the authors see some differences in RNAseq at the L1 larval stage. The authors refer to these as "substantial differences" but there are 63 differentially expressed genes (out of thousands). The authors suggest that the upregulated transcripts in animals whose mothers did not produce intestinal miRNAs are increased because of the lack of inheritance of these intestinal, maternal miRNAs. This seems highly unlikely. The miRNAs that the authors propose may regulate these genes are among the most highly transcribed zygotic miRNAs. To make these claims, the authors should sequence miRNAs from L1s derived from the two conditions and assess whether the maternal contribution plays any role at that stage.

The experiments testing for stress resilience upon degradation of the maternal miRNA biogenesis in the intestine are interesting, but the authors cannot disentangle the possibility that all these effects are due to mothers with "sick intestines" producing worse quality embryos for reasons other than inherited miRNAs. Truly testing the functionality of the inherited miRNAs would require mutational analysis of individual miRNAs and their targets, in our opinion.

We fully agree with the reviewers that testing the functionality of inherited miRNAs through mutational analysis of individual miRNAs and their targets would significantly strengthen the conclusion that yolk-transferred miRNAs can have a functional impact on embryonic gene expression. As the reviewers correctly point out, many of the yolk-inherited miRNAs are also among the most abundantly expressed miRNAs zygotically, making it challenging to disentangle maternal from zygotic contributions at later developmental stages.

To address this, we focused our analysis on miRNAs that are expressed at very low levels in control mothers but show substantial maternal inheritance upon osmotic stress. Among these, *miR-85* exhibited the strongest increase in maternal deposition in response to stress (**Fig. 5c**) and has previously been implicated in targeting heat shock protein (HSP) mRNAs (*Pagliuso et al., PLOS Genetics, 2021*). We performed mRNA sequencing of hatched L1 larvae derived from osmotically stressed versus control *miR-85* mutant mothers and found that *hrg-1*—a predicted miR-85 target and one of the most upregulated transcripts in the DP-depletion experiment (**Fig. 2f and Extended Data Fig. 4a**)—was among the few significantly upregulated genes (**Fig. 6c and Extended Data Fig. 9c**). Additionally, we observed upregulation of several hsp genes, consistent with the previously described role of miR-85 in regulating stress response pathways (**Extended Data Fig. 9e**).

To directly test whether *hrg-1* is regulated by maternal *miR-85*, we mutated the predicted *miR-85* binding site in the *hrg-1* 3′UTR and measured mRNA levels by RT-qPCR. We observed a significant increase in *hrg-1* mRNA in L1 larvae derived from stressed mothers, confirming that the repression depends on the presence of this binding site (**Fig. 6b**).

To rule out zygotic contribution, we exposed *mir-85* heterozygous mothers to osmotic stress and compared hrg-1 expression in heterozygous and homozygous *mir-85* mutant L1 progeny. We observed no significant difference between these genotypes (**Fig. 6a and Extended Data Fig. 9c**), indicating that *hrg-1* repression is independent of zygotic *miR-85* and instead depends on maternally inherited miR-85. Supporting this, we found no detectable differential expression of *miR-85* between heterozygous and homozygous mutant L1s (**Extended Data Fig. 9d**),

consistent with an absence of zygotic expression at this stage. Interestingly, we observed a modest increase in *hrg-1* mRNA in heterozygous L1 progeny from heterozygous mothers compared to wild type, suggesting a dose-dependent effect of maternally inherited miR-85.

While we cannot entirely exclude global maternal effects, the specificity of the *hrg-1* response to deletion of the *miR-85* binding site—and its absence in zygotic *miR-85* mutants—strongly supports a direct, functional role for inherited *miR-85*. Importantly, *mir-85* mutant progeny exhibit minimal transcriptional differences compared to the wild type (only one gene upregulated under control conditions and ten under stress, **Fig. 6c**), in contrast to the hundreds of differentially expressed genes seen following intestinal DP depletion. This broader response may reflect the inheritance of multiple intestinal miRNAs or result from secondary physiological consequences of depleting miRNAs in the maternal intestine. Thus, while DP depletion supports the existence of widespread miRNA-dependent regulation, our targeted experiments with *miR-85* provide functional evidence for specific, inherited miRNA–mRNA interactions.

Altogether, while we think the idea of miRNA inheritance via yolk is sufficiently supported, whether this has any functional consequence is not sufficiently tested.

We thank the reviewers for acknowledging the strength of our evidence supporting miRNA inheritance via yolk. We hope that the new targeted functional experiments focusing on *miR-85* provide compelling evidence in support of the conclusion that maternally inherited miRNAs can indeed regulate embryonic gene expression in the next generation.

Reviewer #5 (Remarks to the Author):

Aupérin, Singh et al. report that provisioning yolk from the intestine to the germline regulates stress responses in progeny in C. elegans. This study characterized small RNAs in yolk originating from the intestine and identified miRNA as a key epigenetic source that impacts transcriptome, development, and survival of larvae under harsh environments. The authors also showed that maternal age and environmental stress affect the yolk-mediated inheritance of miRNA, thereby securing progeny protection.
Overall, the manuscript is clear to follow with the help of the diagrams. Adequate experimental approaches convincingly address yolk-enriched miRNA as a novel heritable epigenetic element. Although yolk inheritance was already known to have beneficial effects on offspring phenotypes, this study extends the field of small RNA-mediated regulation of soma to germline communications and inheritance.

We thank Reviewer #5 for appreciating our manuscript and for highlighting the novelty of yolk-mediated miRNA inheritance and the contribution of our study to the field of small RNA-mediated soma-to-germline communication. Below, we address each of the reviewer's comments.

Major concerns:

1. Links between yolk-enriched miRNA and stress resistance in progeny are vague. The role of lin-4 and miR-235 in oxidative stress responses has been tested only in adult animals in previous studies (ref. 37 and 38 of this paper). It remains determined whether lin-4 and miR-235 in yolk are responsible for oxidative stress resistance in progeny during development. This type of functional validation would further strengthen the major claim of the paper.

We agree that functional validation of specific yolk-inherited miRNAs would strengthen our central claim, and we thank the reviewer for highlighting this important point.

We considered testing *lin-4* and *miR-235* in this context. However, *lin-4* mutants exhibit severe developmental delay even under normal growth conditions, making it technically unfeasible to conduct reliable oxidative stress assays during development. We therefore focused on *miR-235*, but *mir-235* mutants did not show a measurable phenotype in oxidative stress survival assays (**Extended Data Fig. 9a**). This may reflect the relatively modest increase in *miR-235* inheritance observed upon maternal stress exposure (**Fig. 5c**), suggesting a limited functional contribution in this specific context.

Instead, we prioritized *miR-85*, which shows the most robust increase in inheritance following maternal osmotic stress and is predicted to target *hrg-1*, a key stress-responsive gene upregulated in L1 larvae derived from Drosha-Pasha (DP) intestinal depleted mothers. We now provide functional evidence that:

1) *mir-85* mutants display significant developmental delay under oxidative stress conditions, indicating a role in stress resilience (**Extended Data Fig. 9a**).
2) *miR-85* directly represses *hrg-1* expression in a sequence-specific manner through its predicted 3′UTR binding site (**Fig. 6b, c**).
3) This repression is inheritance-dependent, as zygotic expression of *miR-85* alone is insufficient to drive target repression (**Fig. 6a and Extended Data Fig. 9c**).

These new data provide direct functional validation of one yolk-inherited miRNA (*miR-85*) in conferring stress resilience during development, thus strengthening the mechanistic basis for our proposed model.

2. The authors did not fully rule out the potential effects of auxin/5-Ph-IAA treatment -independently of degron-mediated degradation of target proteins- on observations made with the degron strains, such as VIT-2::GFP accumulation in oocytes (Fig. 1d) as well as stress resilience phenotypes of progeny (Fig. 4 and Fig. 6). Inclusion of control strains preferably such that express degron-tagged RME-2 or DP but do not carry the TIR1 transgene would clarify this concern.

We appreciate the reviewer's suggestion and have now included experiments using RME-2 and DP degron strains lacking TIR1, treated with or without auxin. These controls confirm that auxin alone does not induce VIT-2::GFP accumulation in the pseudocoelom or impair yolk inheritance (**Extended Data Figs. 1b,h, 3b**), nor does it affect larval development under oxidative stress (**Extended Data Fig. 6d**). These results demonstrate that the phenotype we observe is due to targeted protein depletion and not off-target effects of auxin or 5-Ph-IAA.

3. The phenotypic variability without exposing to stressors that can also be affected by loss of yolk-enriched miRNA needs to be considered to assess stress resilience in Fig. 4 and Fig. 6. Additional controls without stress conditions should be included to address this concern.

We agree and have now included additional controls under non-stress conditions. Larvae derived from DP degron mothers develop normally when not exposed to stress (**Extended Data Fig. 6e**). This finding supports the

conclusion that the observed phenotypic variability is specific to stress conditions and related to miRNA inheritance rather than being a general developmental defect.

Additional concerns:

1. In Extended Data Fig. 1b, images of VIT-2::GFP with auxin treatment and VIT-2::GFP; RME-2 degron without auxin treatment should also be included.

We have now included these additional images in **Extended Data Fig. 1b** as requested, showing VIT-2::GFP; RME-2 degron without TIR-1 and with auxin treatment and VIT-2::GFP; RME-2 degron without auxin.

2. "Increasing paraquat concentrations were associated with compromised development and decreased larval lengths, as expected (Fig. 4a)"(lines 196-198 in the text). However, compromised development was not proved in Fig. 4a. For instance, a similar experiment but with paraquat as conducted in Fig 6b, d would help address this.

We have now quantified and included the percentage of L1 arrest under paraquat treatment to support the claim of compromised development with increasing concentrations (**Extended Data Fig. 6a**).

3. The authors should complement the comparison of yolk-enriched miRNA from old mothers and NaCl-treated mothers by adding that from mothers exposed to starvation (Extended Data Fig. 5c): are there any yolk-enriched miRNA inherited irrespective of maternal conditions tested?

We have added this comparison in **Extended Data Fig. 7f**. We found no miRNAs with consistently increased inheritance across all maternal conditions, suggesting that specific environmental cues shape the inherited miRNA repertoire.

4. For clarity of the control condition for osmotic stress, it would be better to explain that standard NGM plates contain 50 mM NaCl in legends and methods.

We have clarified in the figure legends and methods that standard NGM plates contain 50 mM NaCl, which serves as the baseline for osmotic stress experiments.

5. Providing genetic information on tissue-specific degron strains in the text or legend would help readers understand experiments without access to supplementary tables of a complete strain list.

We have now included relevant genetic details of the degron strains used in each figure legend and the main text for clarity.

We hope these revisions and new experiments satisfactorily address Reviewer #5's concerns and further strengthen our manuscript.

We are grateful for the careful evaluation of our work during the previous review process and for the thoughtful and constructive feedback provided by all reviewers. We carefully considered the remaining concerns regarding the functional consequences of yolk-inherited miRNAs and, in this revised submission, have strengthened the manuscript through the addition of new experiments and controls that directly address these points. Changes made to the manuscript in response to reviewer comments are highlighted in purple in the revised text.

Reviewer #1 (Remarks to the Author):

I thank the authors for addressing my concerns and for performing experiments on the regulation of hrg-1 by miR-85. The proteomic analysis presented also add to the evidence suggesting that miRNAs transported along with yolk could function in embryos. Together, these findings strengthen the paper and I congratulate the authors on this important contribution to our understanding.

We sincerely thank Reviewer #1 for their positive feedback and for recognizing the strength of the additional experiments, including the regulation of *hrg-1* by *miR-85* and the proteomic analysis. We are grateful for the reviewer's congratulations and for acknowledging the significance of our contribution to the understanding of this epigenetic paradigm.

Reviewer #2 (Remarks to the Author):

The authors have properly addressed my concerns in their revised manuscript. Importantly, the new results further strengthened their conclusion on the direct role of yolk-inherited miRNAs in the stress resilience of C. elegans progeny. Aside from a few very minor questions and suggestions that I believe the authors can clarify in the manuscript; I continue to view this as a breakthrough study that will open a new field for investigating the roles of yolk-carried small RNAs in intergenerational gene regulation. I fully recommend this manuscript for its publication.

Sincerely,
Heng-Chi Lee

We thank Reviewer #2 for their full support of our study and for recognizing the breakthrough nature of our work. We are pleased that the revised manuscript and new results have addressed the reviewer's concerns and reinforced our conclusions regarding the role of yolk-inherited miRNAs in promoting stress resilience in *C. elegans* progeny. Below, we respond to the reviewer's additional comments and suggestions.

Minor comments/suggestions:
1. While the authors show that purification of yolk granules appears to enrich for VIT-GFP in wild-type animals, the extent of this enrichment remains unclear. Additionally, the figures do not show any granularity of VIT-1::GFP. Could the authors clarify whether yolk granules were observed in the body cavity, embryos, or other tissues?

In the sorting experiment, the approach is qualitative rather than quantitative with respect to enrichment. Moreover, the granular ultrastructure of yolk has been resolved primarily by immuno-electron microscopy (Zhai et al., 2024) and cannot be visualized at comparable resolution by GFP fluorescence microscopy. We have clarified this limitation and explicitly state that yolk granules accumulate in the body cavity and are subsequently taken up by developing oocytes.

2. Given that many piRNAs and 22G-RNAs are germline-enriched, could the authors speculate why piRNAs and 22G-RNAs are not depleted in the yolk-enriched small RNA pool (Figure 1B)?

We speculate that piRNAs and 22G-RNAs, while abundant in the germline, are not selectively enriched in yolk because they are predominantly retained within germline-associated Argonaute complexes rather than packaged into yolk particles produced in the intestine. Low-level detection of these small RNAs in the yolk-enriched fraction may additionally reflect minor carryover during purification. We have clarified this interpretation in the manuscript text.

3. The nomenclature "DE miRNA" and "DP degron" may be difficult to interpret and potentially confusing to readers. Would the authors consider using more descriptive terms such as "RME-2–dependent miRNAs" or "intestinal miRNA depletion"?

We thank the reviewer for this helpful suggestion. We have revised the nomenclature in the manuscript to enhance clarity for the reader.

4. In Figure 4A, under non-paraquat conditions, do larvae from day 3 mothers grow faster than those from day 1 mothers at 72 hours? Currently, Extended Data Figure 6E shows results at 44 hours, but not at 72 hours as in the main figure. It would be helpful to include this comparison.

We appreciate this suggestion. Under non-paraquat conditions, larvae develop normally without noticeable delays and reach the L4 stage by 48 hours post-hatching (hph). In contrast, under paraquat stress, development is slowed, and animals typically reach L4 only around 72 hph, which is why that later time point is used in the main figure.

To compare animals at the same developmental stage across conditions, we therefore measure larval length at 48 hph in non-paraquat conditions and at 72 hph in paraquat-treated animals. By 72 hph under non-stress conditions, larvae have already reached adulthood, and the COPAS sorter cannot reliably distinguish their size due to morphological uniformity.

Regarding the effect of maternal age on larval development under non-stress conditions, Perez et al. (2017) have already shown that progeny of Day 3 mothers grow better than those of Day 1 mothers. Our findings are consistent with this observation (**Extended Data Fig. 6f**).

5. The nature of the mir-85 binding site mutation generated in the study should be clearly described in Extended Data Figure 4—whether it involves a specific nucleotide change in the seed sequence or a deletion of the entire site. Additionally, the predicted base-pairing between mir-85 and hrg-1 should be shown.

We thank the reviewer for this suggestion. We have clarified in the **Fig. 6b** legend that the mutation involves a precise nucleotide substitution in the predicted seed sequence of the *mir-85* binding site. We have also included a schematic in **Fig. 6b** showing the predicted base-pairing between *mir-85* and the *hrg-1 3' UTR* to illustrate the targeting interaction.

Reviewer #3-4 (Remarks to the Author):

In this version of the manuscript, Auperin et al. address multiple concerns from the previous round of review. We commend the authors' efforts and the carefully controlled experiments, such as showcasing the specificity of the tissue-specific degron system, the technical details of the sorting strategy, and highlighting the relatively invariant level of germline miRNAs. Taken together, the evidence convincingly demonstrates yolk-mediated miRNA transfer to the embryo.

However, the latter half of the manuscript, which focuses on the potential functional implications of this observed phenomenon, remains incomplete. To maintain the key claim in the title and the overall work, the authors actually need to demonstrate the functionality of these yolk-associated miRNAs.

We thank the reviewer(s) for their careful assessment and for recognizing the strength of the evidence supporting yolk-mediated miRNA transfer to embryos. We appreciate the emphasis placed on clarifying the functional consequences of this inheritance and have substantially revised the latter half of the manuscript to address these points. In particular, we have added new experiments, incorporated additional controls, and refined our interpretation to clearly distinguish between the sequence-specific regulatory activity of individual yolk-inherited miRNAs and the broader, pooled contribution of multiple miRNAs to progeny physiology.

We address each concern below.

The claim of functionality is first based on the experiments with the DP-degron and mothers of different ages, using paraquat resistance as a readout (Figure 4). In that experiment, it is clear that maternal age, which correlates with yolk deposition, correlates with paraquat resistance. However, in that context, removing intestinal miRNAs has a small contribution. The conclusion from this experiment should be that other components of yolk, or other aspects of maternal age contribute to that resistance.

We agree with this interpretation and have revised the Results and Discussion accordingly. We now explicitly state that maternal age–dependent resistance to oxidative stress reflects multiple yolk-associated components and that intestinal miRNAs contribute functionally but are not solely responsible for this phenotype. This revised interpretation is now clearly articulated in the Results section associated with **Fig. 4** and reiterated in the Discussion.

In the second set of experiments, the authors use the degron again but now in an osmotic stress paradigm (Figure 7). In that context, it looks like miRNAs play a more prominent role. This experiment is done with mothers of unspecified age. It would be good to know the precise ages and preferably compare Day 1 and Day 3. But most importantly, to interpret this and the previous experiment, we are missing the direct testing of yolk accumulation with maternal age in the DP genetic background under auxin treatment. A well-executed but incomplete control was performed in Extended Data Fig. 3, demonstrating that DP-degron mothers do not have gross yolk transfer defects; however, this analysis was limited to embryos from mothers of unspecified ages. It would be necessary to confirm that, at Day 3 with auxin treatment, yolk still accumulates more than in Day 1 mothers and is not disrupted by intestinal DP-degron treatment. This would allow the effect to be more confidently attributed to miRNAs rather than to other general components of the yolk.

To address this concern, we have performed direct measurements of yolk accumulation in embryos from Day 1 and Day 3 DP degron mothers grown with auxin. As shown in **Extended Data Fig. 6e**, age-dependent yolk accumulation (Day 3 > Day 1) is preserved under intestinal miRNA depletion, demonstrating that auxin-induced DP depletion does not disrupt yolk production or age-dependent yolk loading. Moreover, we have performed similar experiments for the osmotic stress paradigm, confirming that DP degron mothers, grown with auxin and exposed to osmotic stress, transfer more yolk to the progeny, similar to control mothers (**Extended Data Fig. 9c**). These results allow the observed progeny phenotypes to be attributed to loss of yolk-associated miRNAs rather than defective yolk transfer.

Regarding the maternal age of the osmotic stress experiment, we have used day 1 adult mothers, in line with the established protocols used in previous studies (Burton et al., 2017 and 2021). Since maternal age was not a variable in those paradigms, we maintained the same conditions to ensure comparability and to avoid introducing potential confounding effects related to maternal age. The maternal ages are now explicitly stated for the osmotic stress experiments in the revised manuscript.

The next experiment the authors use to support the role of intestinal miRNAs in the next generation is the characterization of the miR-85 mutants. The authors test one of the original deletion alleles, which was generated by heavy mutagenesis, in the paraquat resistance assay. Without rescue and/or generation of an independent CRISPR allele, this experiment should not be interpreted as proof that this miRNA is involved in stress.

To address this point, we generated an independent CRISPR–Cas9 *miR-85* mutant allele. This mutant recapitulates the derepression of *hrg-1* observed in the original *miR-85(n4117)* mutant in progeny derived from osmotically stressed mothers (**Extended Data Fig. 11c**), excluding linked background mutations as the cause of this effect. Moreover, RNA-seq analysis revealed that the CRISPR–Cas9 *miR-85* mutant exhibits the same stress-induced *hsp* gene expression signature observed in the original *miR-85(n4117)* allele (**Extended Data Fig. 11e**), further supporting a specific role for *miR-85* in regulating stress-responsive targets.

Consistent with this specificity, our RNA-seq dataset shows that the *miR-85(n4117)* mutant exhibits minimal global transcriptional changes under control conditions (50 mM NaCl), with only one gene significantly upregulated (**Fig. 6c**). Under osmotic stress (300 mM NaCl), only a limited set of genes is affected (10 upregulated genes, including *hrg-1* and *hsp-70*). This constrained, stress-dependent transcriptional signature further supports a targeted regulatory role for *miR-85*, in contrast to the broad transcriptional changes observed upon intestinal miRNA depletion (**Extended Data Fig. 10a**).

It is also unclear why the authors then switch again the stress context in which they study the effects of miR-85 on gene expression, but they do not show the effects of miR-85 mutation on their osmotic stress paradigm.

We have now addressed this experimentally. We tested the *miR-85* mutant in the inherited osmotic stress paradigm and showed that loss of *miR-85* partially attenuates stress resilience in progeny derived from stressed mothers (**Extended Data Fig. 12**). Importantly, this effect is weaker than that observed upon intestinal DP depletion, which abolishes the inherited stress adaptation (**Fig. 7b**). This difference is consistent with a model in which intestinal DP depletion disrupts the transfer of multiple yolk-inherited miRNAs, resulting in broad transcriptional changes and a stronger impairment of stress resilience than loss of a single miRNA. This distinction is now explicitly stated in the Results and Discussion.

Finally, the authors try to connect miR-85 to one of its deregulated targets, hrg-1. The binding site mutation experiment is an elegant way to evaluate the possible direct effect of miR-85 on hrg-1. However, hrg-1 expression levels in the binding site mutant worms show enormous variation. Without the one higher datapoint, the other two datapoints would indicate that the binding site mutant looks just like wild type. The use of a non-parametric test with these three datapoints is not very meaningful. Moreover, there is no direct functional link provided here, as

there is no phenotype evaluation for hrg-1 binding site mutants. For instance, if these mutants had any effect on L1 survival similar to Figure 7 could strengthen this argument. The evidence showing derepression of hrg-1 mRNA alone is insufficient to attribute resilience as being mediated by miR-85. A discussion in the text providing a model or speculation about the role of hrg-1 in stress responses should also be included.

To address concerns regarding variability, we quantified *hrg-1* expression using RNA-seq and replaced non-parametric testing with RNA-seq–based DESeq2 analysis rather than single-gene assays. Across several biological replicates (n = 5) for the *hrg-1* 3′UTR binding-site mutant and two independent *miR-85* mutants, *hrg-1* is consistently derepressed under osmotic stress (**Fig. 6b; Extended Data Fig. 11c**), supporting a robust, sequence-specific regulatory effect.

We agree that derepression of a single target is insufficient to account for the full stress resilience phenotype. We have therefore revised our interpretation and present *hrg-1* as a mechanistically tractable example of sequence-specific regulation by a yolk-inherited miRNA, rather than as the sole effector of stress resilience. We now include a model in the Discussion describing how modulation of heme-responsive genes such as *hrg-1* may contribute to tuning stress responses during early development, while emphasizing that stress resilience likely emerges from the combined action of multiple yolk-inherited miRNAs.

Taken together, these additional experiments demonstrate that yolk-inherited miRNAs are functionally active in progeny and can execute sequence-specific gene regulation in embryos, while clarifying the relationship between individual miRNAs and the broader phenotypes observed upon intestinal miRNA depletion. We therefore emphasize that our central claim is not that individual yolk-inherited miRNAs are sufficient to recapitulate complex stress phenotypes, but that maternally transferred somatic miRNAs are biologically active in embryos and contribute, in combination, to progeny stress resilience.

Minor points:

Figure 4: in this experiment, the goal was to test whether embryos from mothers of different ages exhibit differential resilience to paraquat-induced stress, using L1 length as a readout of worm growth. However, there is no control condition without paraquat included. Extended Data Fig. 6e provides an incomplete control condition, but the measurement was performed at a different time point (44 hours instead of the 72 hours used in other assays) and includes only progeny from Day 1 mothers. As a result, it is challenging to establish a direct link using this readout. A similar control group setup, such as the one used in Extended Data Fig. 6a, would be helpful to demonstrate whether the effect is paraquat-dependent.

We have clarified this point and added the relevant controls. Under non-stress conditions, larvae develop rapidly and reach adulthood by 48 hours post-hatching, whereas stress conditions delay development, requiring 72 hours to obtain developmentally comparable populations. Accordingly, growth was assessed at 48 hours in the absence of stress and at 72 hours under osmotic stress. These controls are now included in **Extended Data Fig. 6f,g**, and the rationale for the different time points is explicitly stated in the figure legend.

Page 28 Extended Data Fig. 1e:
The legend describes this as a western blot, but no antibody was specified for the blot. Based on the description in the method section (Page 46, Line 916 to Page 47, Line 920), it appears to be an anti-GFP blot. Labeling the band and specifying the antibody would better orient readers.

We thank the reviewer for this helpful suggestion.

Minor Formatting:
1. Page 3 Line 80:
C. elegans should be italicized.

We have corrected the text accordingly.

Reviewer #5 (Remarks to the Author):

In the revised version the authors have adequately addressed and clarified all my comments on the original manuscript. The inclusion of functional validation data and additional controls make this now a very convincing paper that clearly advances the field.

We thank Reviewer #5 for their supportive assessment and for acknowledging that the additional functional validation and controls have made the revised manuscript more convincing. We are pleased that the reviewer considers our study to clearly advance the field, and we are grateful for their constructive input throughout the review process.

Minor suggestion:

The authors may want to add the age of TIR1-negative DP degron mothers in the legend of Extended Data Fig6(d) in the revised manuscript.

We will add the age of TIR1-negative DP degron mothers in the legend of **Extended Data Fig. 6d**.

Björn Schumacher

Reviewer #3 (Remarks to the Author):

In this version of manuscript, Auperin et al. include extra controls and clarify important points in their model for yolk miRNA transmission and its functional consequences. We commend the authors's effort to address our previous concerns.

A minor point:
Pg 38. Ext fig 6e
Please check the label for the auxin groups, should it be -. -. +, +?

Reviewer #4 (Remarks to the Author):

I co-reviewed this manuscript with one of the reviewers who provided the listed reports. This is part of the Nature Structural & Molecular Biology initiative to facilitate training in peer review and to provide appropriate recognition for Early Career Researchers who co-review manuscripts.

We thank reviewers' #3 and #4 for aknowledge our effor to address all the previous remaining concerns. We also thank them to point out to a typo in Extended Fig 6e, which we have corrected.
